# A Systematic Review of Plants With Antibacterial Activities: A Taxonomic and Phylogenetic Perspective

**DOI:** 10.3389/fphar.2020.586548

**Published:** 2021-01-08

**Authors:** François Chassagne, Tharanga Samarakoon, Gina Porras, James T. Lyles, Micah Dettweiler, Lewis Marquez, Akram M. Salam, Sarah Shabih, Darya Raschid Farrokhi, Cassandra L. Quave

**Affiliations:** ^1^ Center for the Study of Human Health, Emory University, Atlanta, GA, United States; ^2^ Emory University Herbarium, Emory University, Atlanta, GA, United States; ^3^ Department of Dermatology, Emory University, Atlanta, GA, United States; ^4^ Molecular and Systems Pharmacology Program, Laney Graduate School, Emory University, Atlanta, GA, United States

**Keywords:** ethnopharmacology, antibacterial, medicinal plants, antimicrobial, minimum inhibitory concentration

## Abstract

**Background:** Antimicrobial resistance represents a serious threat to human health across the globe. The cost of bringing a new antibiotic from discovery to market is high and return on investment is low. Furthermore, the development of new antibiotics has slowed dramatically since the 1950s’ golden age of discovery. Plants produce a variety of bioactive secondary metabolites that could be used to fuel the future discovery pipeline. While many studies have focused on specific aspects of plants and plant natural products with antibacterial properties, a comprehensive review of the antibacterial potential of plants has never before been attempted.

**Objectives:** This systematic review aims to evaluate reports on plants with significant antibacterial activities.

**Methods:** Following the PRISMA model, we searched three electronic databases: Web of Science, PubMed and SciFinder by using specific keywords: “plant,” “antibacterial,” “inhibitory concentration.”

**Results:** We identified a total of 6,083 articles published between 1946 and 2019 and then reviewed 66% of these (4,024) focusing on articles published between 2012 and 2019. A rigorous selection process was implemented using clear inclusion and exclusion criteria, yielding data on 958 plant species derived from 483 scientific articles. Antibacterial activity is found in 51 of 79 vascular plant orders throughout the phylogenetic tree. Most are reported within eudicots, with the bulk of species being asterids. Antibacterial activity is not prominent in monocotyledons. Phylogenetic distribution strongly supports the concept of chemical evolution across plant clades, especially in more derived eudicot families. The Lamiaceae, Fabaceae and Asteraceae were the most represented plant families, while *Cinnamomum verum*, *Rosmarinus vulgaris* and *Thymus vulgaris* were the most studied species. South Africa was the most represented site of plant collection. Crude extraction in methanol was the most represented type of extraction and leaves were the main plant tissue investigated. Finally, *Staphylococcus aureus* was the most targeted pathogenic bacteria in these studies. We closely examine 70 prominent medicinal plant species from the 15 families most studied in the literature.

**Conclusion:** This review depicts the current state of knowledge regarding antibacterials from plants and provides powerful recommendations for future research directions.

## Introduction

Antimicrobial resistance (AMR) threatens the ability to successfully treat infectious diseases across the globe (McEwen and Collignon, 2018). While many factors such as the unnecessary prescription of antimicrobials and their use in agriculture contribute to the spread of AMR, a combination of scientific and economic challenges in antimicrobial development has caused the pipeline of new drugs to decline (WHO, 2019a; WHO, 2019b). Annual deaths worldwide due to AMR continue to climb to around 750,000 and are projected to reach as high as 10 million by the year 2050 (O’Neill, 2016). Whereas AMR refers to resistance in bacteria, fungi, viruses and protozoans, antibiotic resistance refers to resistance specifically in bacteria, which is the focus of this review.

To counteract the lack of new antibacterials and the rise of antibiotic resistance, plants could represent a potential solution. Indeed, plants are equipped with an array of effective defense mechanisms, such as the production of secondary metabolites, to combat pests and pathogens before they are able to cause serious damage. Plants and other organisms have co-evolved for more than 350 million years (Magallón and Hilu, 2009; Clarke et al., 2011) and have developed strategies to overcome each other’s defense systems. Plant secondary metabolites play a major role in how plants adapt to their environment; they also act as their surveillance system. They are byproducts of non-essential metabolic pathways and are responsible for the specific odors, tastes and colors of plant tissues. Plant secondary metabolites can also help the plant to cope with abiotic stresses (e.g., UV radiation) and to communicate with other organisms (e.g., herbivores, pathogens, neighboring plants, pollinators and fruit dispersers), so they are also important for growth and development (Kessler and Kalske, 2018; Zaynab et al., 2018; Wink, 2020). These compounds usually belong to one of three large chemical classes known for biological activity: terpenoids, phenolics and alkaloids. In particular, terpenoids represent one of most diverse secondary metabolite groups and include more than 50,000 known compounds (Chassagne et al., 2019; Belcher et al., 2020).

Plants are also highly biodiverse. The most recent estimates place the total number of plant species at approximately 374,000 (Christenhusz and Byng, 2016). This includes described and accepted vascular plant species, of which 295,383 are angiosperms (monocots: 74,273, eudicots: 210,008), 1,079 are gymnosperms, 10,560 are ferns, 1,290 are lycopods, and the rest are bryophytes and algae (Christenhusz and Byng, 2016). This represents ten times as many species as all terrestrial vertebrates combined. With regards to geographic location, the most species-rich areas of the world with an estimated 44% of all plant diversity are confined to 25 hotspots with 1.4% of the total land area on the planet (Myers et al., 2000). These areas remain richly biodiverse with approximately 290,000 unstudied plant species with countless secondary metabolites (RBG and Willis, 2017).

Throughout history, humans have relied on plants as a source of medicine. Evidence for the historical role of plants in treating human disease is documented by the long history of medical texts from civilizations across the globe. The oldest written record of medicinal plants, dated 2600 BCE, was written in cuneiform on clay tablets in Mesopotamia and recorded the use of oils from the Mediterranean cypress tree (*Cupressus sempervirens* L., Cupressaceae) and opium poppy (*Papaver somniferum* L., Papaveraceae) (Cragg and Newman, 2005). The next oldest written record was found in Egypt, the 3,500-year-old Ebers Papyrus (1553-1550 BCE) (Bryan, 1930). The Ebers papyrus is a well-preserved scroll detailing a variety of plants traditionally used for a wide range of diseases in ancient Egypt (Aboelsoud, 2010).

Today, in many parts of the developing world, between 70 and 95% of people continue to rely on plants as a primary form of medicine, and many countries have integrated traditional plant-based medicines through regulations into mainstream healthcare systems (RBG and Willis, 2017). Plant-based medicines also continue to make up a key component of intercultural healthcare, encompassing biomedical and traditional medical approaches, in minority and underserved communities (Vandebroek, 2013). According to the Medicinal Plant Names Services (MPNS), 28,187 species (∼7.5% of all plant species on Earth) are recorded as being used medicinally (MNPS, 2020), but only 4,478 are cited in medicinal regulatory publications (RBG and Willis, 2017).

Complex mixtures of compounds found in nature represent an interesting source of bioactive compounds, as they can act in synergy. Mechanisms by which plant compounds can synergize in this way include targeting multiple receptors, facilitating transport to a target, protection from degradation and modification of resistance (Gilbert and Alves, 2003). The widespread presence of synergistic interactions in plant extracts is evidenced by the frequent loss of activity upon fractionation (Inui et al., 2012; Abreu et al., 2017). There is a growing recognition in the field that the pursuit of single active compounds through bioassay-guided fractionation is not sufficient to capture the potential of plant compounds as antibacterials—synergy and other interactions must be studied (Caesar and Cech, 2019). In addition to the study of interactions within plant extracts, many recent innovations involve finding plant compounds that synergize with existing antibiotics, particularly as resistance-modifying agents for use against drug-resistant bacteria (Abreu et al., 2012; Abreu et al., 2017; Dettweiler et al., 2020).

In cases where synergy is found in plant extracts, options such as the FDA Botanical Drug Guidance exist for the development of whole extracts or refined fractions as drugs (FDA, 2016). Two botanical drugs have been approved by the FDA: Veregen (sinecatechins), a green tea extract (*Camellia sinensis* (L.) Kuntze, Theaceae) for the treatment of genital and perianal warts, and Fulyzaq or Mytesi (crofelemer) from the dragon’s blood tree (*Croton lechleri* Müll.Arg., Euphorbiaceae), used to treat diarrhea associated with HIV anti-retroviral therapy (Wu et al., 2020).

Thousands of laboratory studies have been conducted on the antibacterial potential of plants since the 1940s. However, a comprehensive review of this body of work, with rigorous inclusion and exclusion criteria based on the scientific rigor of studies, has never before been attempted. While it is well known that certain plants exhibit antibacterial properties in laboratory studies, little is still understood about which specific botanical taxa have exhibited the most promising activity against pathogenic bacteria to date. Here, we present a comprehensive analysis of the literature on plants used as antibacterials by focusing on their reported growth inhibitory activity.

## Methods

### Terminology

#### Botanical Terminology

All reported plant names were cross-checked for accuracy in accordance with The Plant List (http://www.theplantlist.org/), the International Plant Names Index (http://www.ipni.org/index.html), the World Checklist of Selected Plant Families (https://wcsp.science.kew.org/) and Tropicos (https://www.tropicos.org/home). Plant family assignments follow the Angiosperm Phylogeny Group IV guidance (APG et al., 2016). Any botanical synonyms or citations with unaccepted author epithets were updated to the current corrected nomenclature and are reported as such both in the supplementary material data tables and manuscript text.

#### Antibiotic Terminology

In this review, we define antimicrobials as agents that inhibit the growth of microbes (bacteria, fungi, viruses, and protozoans). We define antibiotics as agents that inhibit the growth of bacteria specifically, and we consider antibiotic effects to be synonymous with antibacterial effects. The minimum inhibitory concentration (MIC) is defined as the lowest concentration of an antimicrobial agent that inhibits visible growth of a microorganism *in vitro* (CLSI, 2012). This value is commonly used as an indicator of antimicrobial potency. In combination with pharmacokinetic/pharmacodynamic parameters, the MIC is also used to predict the antimicrobial efficacy *in vivo* (Drusano et al., 2004). We also define the MIC as the lowest concentration of an antimicrobial agent that inhibits 90% of bacterial growth as detected by optical density measurement of liquid culture medium. The IC_50_ refers to the concentration of agent inhibiting 50% of the bacterial growth as measured and reported by optical density. All reported bacterial names were cross-checked for accuracy and updated in accordance with the List of Prokaryotic names with Standing in Nomenclature (LPSN) at https://www.bacterio.net/.

### Literature Search

We followed the Prisma guidelines to perform our literature search (Moher et al., 2009). We systematically assessed the scientific literature collected from the Web of Science, PubMed and SciFinder scientific databases, considering all the articles published between 1946 and 2019 (September 3, 2019). The keywords “plant,” “inhibitory concentration” and “antibacterial” were used.

### Inclusion and Exclusion Criteria

Article titles and abstracts were manually screened to exclude studies not related to the topic. Only vascular plants (i.e., angiosperms, gymnosperms, ferns and lycopods) were included in the analysis. Relevant articles were examined to determine fit to the eligibility criteria of this review.

The specific inclusion criteria include the following:

(1) Validated source of material: plant materials were identified to the taxonomic level of genus and species with plant voucher specimens collected and deposited in a herbarium, university or research institute.
(2) Appropriate methodology: standard methods for antibacterial assays were employed using broth microtiter dilution methodologies following established criteria (CLSI, EUCAST, NCCLS) (Eloff, 1998; Andrews, 2001; Cos et al., 2006; Sarker et al., 2007), bacterial inoculum (1 × 10^5^ CFU/mL ≤ inoculum ≤ 1 × 10^7^ CFU/mL equivalent to 0.5 McFarland (1.5 × 10^8^) with dilution from 1:15 to 1:300) and time of incubation (≤24 h, except *Mycobacterium* sp. and *Helicobacter pylori*).
(3) Appropriate MIC or IC_50_ values (Gibbons, 2004; Cos et al., 2006; Aro et al., 2019): for plant extracts, only MICs ≤500 μg/mL or IC_50_ ≤ 100 μg/mL were included. Reported IC_90_ values were considered the MIC.
(4) Access to the full-text article in the English language. Articles published only in non-English languages were excluded.

### Data Extraction

Data are tabulated in Supplementary Material S1 and were also deposited in the Shared Platform for Antibiotic Research and Knowledge (SPARK) with Pew Charitable Trusts, accessible at https://app.collaborativedrug.com/vaults/4724/protocols. Registration for a SPARK account is free and available at http://www.pewtrusts.org/spark-antibiotic-discovery.

Whenever provided, information on the resistance background of strains tested or the ethnopharmacological uses of the plant species is included in Supplementary Material S1. Plant tissue extracted and extraction methodology was also noted when provided in the original publication. The units of MIC and IC_50_ were converted to either µg/mL or µL/mL for plant extracts and essential oils (EOs), respectively. Categories of ethnomedical use were assigned to the data set by organ system, divided across 13 categories: Cardiovascular, Dermatological, Endocrinological, Gastrointestinal, General Health, Genitourinary, Gynecology and Andrology, Musculoskeletal, Neurological and Mental Health, Ophthalmological, Oral Health, Otolaryngological and Respiratory and Not Specified. The International Statistical Classification of Diseases and Related Health Problems 10^th^ Revision (ICD-10) (WHO, 2016) was considered, but ultimately not used due to lack of specificity of disease origins in the reviewed literature. Information on the use of plant extracts in clinical trials was obtained from clinicaltrials.gov (NLM, 2020). Based on the same literature search, we examined the antibacterial activities of plant-derived compounds in another review (Porras et al., 2020).

### Data Analysis

Data obtained from the literature search was maintained and organized in Microsoft Excel, then analyzed and visualized using Graphpad Prism 8; analysis of MIC values was done by one-way ANOVA with Tukey’s multiple comparisons test. Statistical significance was defined as *p* value <0.05.

To generate the euphyllophyte tree, text file R20160,415_euphyllophyte.new (Gastauer and Meira Neto, 2017) in Newick format was used as the backbone. Plant families were graphically displayed using iTOL (Letunic and Bork, 2006). The resulting euphyllophyte family tree from R20160415.new has high resolution, containing only branches with confidence levels (Bootstrap values or posterior probabilities from Bayesian analysis) larger than 80%. It includes 13 gymnosperm, 37 monilophytes families and all the 64 orders and 416 Angiosperm families recognized by APG IV (APG et al., 2016; Gastauer and Meira Neto, 2017). Percentages of studied genera calculated using the total number of genera and species for each family are reported according to the Angiosperm working group version 14 (Stevens, 2001) (Supplementary Material S2.1).

## Results

### Literature Assessment

**FIGURE 1:**
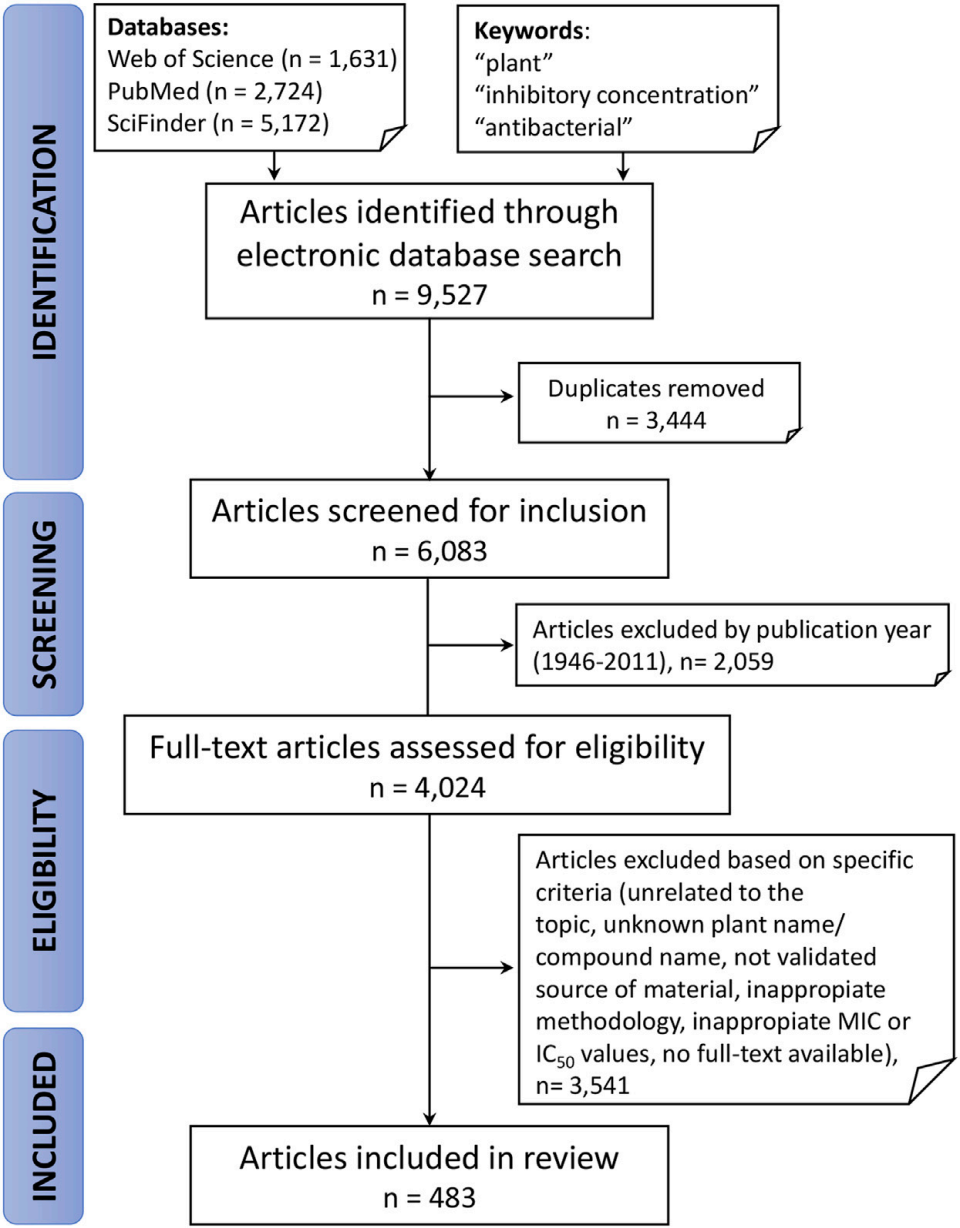
Representation of inclusion and exclusion criteria, with corresponding results from the literature search used to construct the databases in Supplementary Material S1.

**FIGURE 2:**
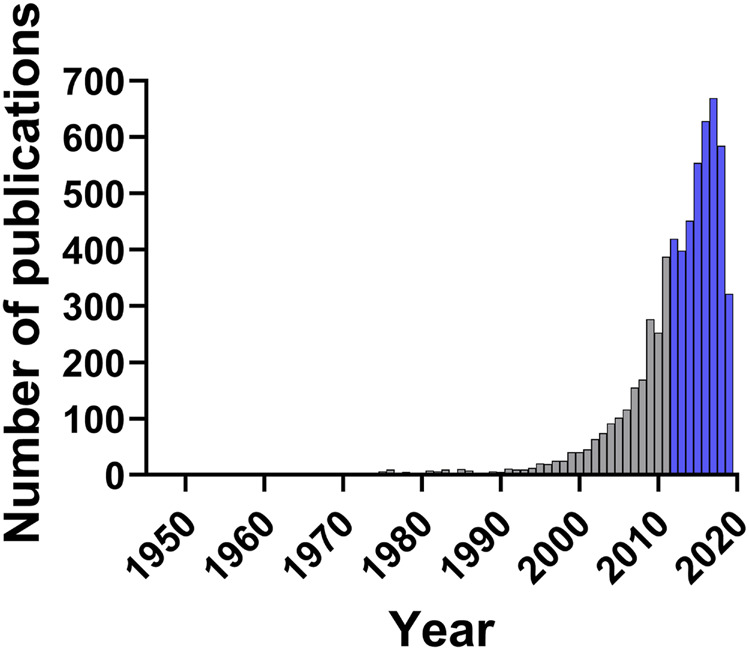
Number of publications per year in the search range from 1946 to September 3, 2019. Search keywords were “plant,” “inhibitory concentration” and “antibacterial.” Years discussed in this review (2012–2019) are highlighted in blue.

The process of identification and screening of articles for this review is represented in Figure 1. The literature search yielded 9,527 articles from which 3,444 duplicates were excluded. Of these 4,024 publications, only 483, or 12%, passed the rigorous criteria required for inclusion in this review. This highlights the need for broader implementation of robust standards for research in this space—from authentication of botanical starting materials to application of standardized laboratory approaches in the evaluation of plant extracts. Nevertheless, as shown in Figure 2, the number of publications related to plants with antibacterial activity has increased significantly in the last decade. In accordance with these trends and the broad application of standardized methods for the antibacterial assessment of extracts, the present review focuses on studies published from January 1, 2012 to September 3, 2019, representing 4,024 articles, or 66% of all papers published under these search criteria since 1946.

In the following sections, we provide an overview of the 958 plant species (representing 4,943 MIC values) with reported antibacterial activity recorded in our literature search (Supplementary Material S1). We discuss their diversity from a taxonomic and phylogenetic point of view, along with their geographic distribution. Other descriptive analyses focus on the extraction types, plant tissues and the most targeted bacteria. Species investigated in clinical trials and those tested against the greatest number of bacteria are also presented. A statistical analysis of the MIC values found for each plant extract was performed based on botanical families, extraction types, plant tissues and species (including fractions and EOs). Lastly, we take an in-depth look into the reported antibacterial activities of 70 plant species from the top 15 reported plant families.

### Phylogenetic, Taxonomic and Geographic Diversity Among Antibacterial Plants

**FIGURE 3:**
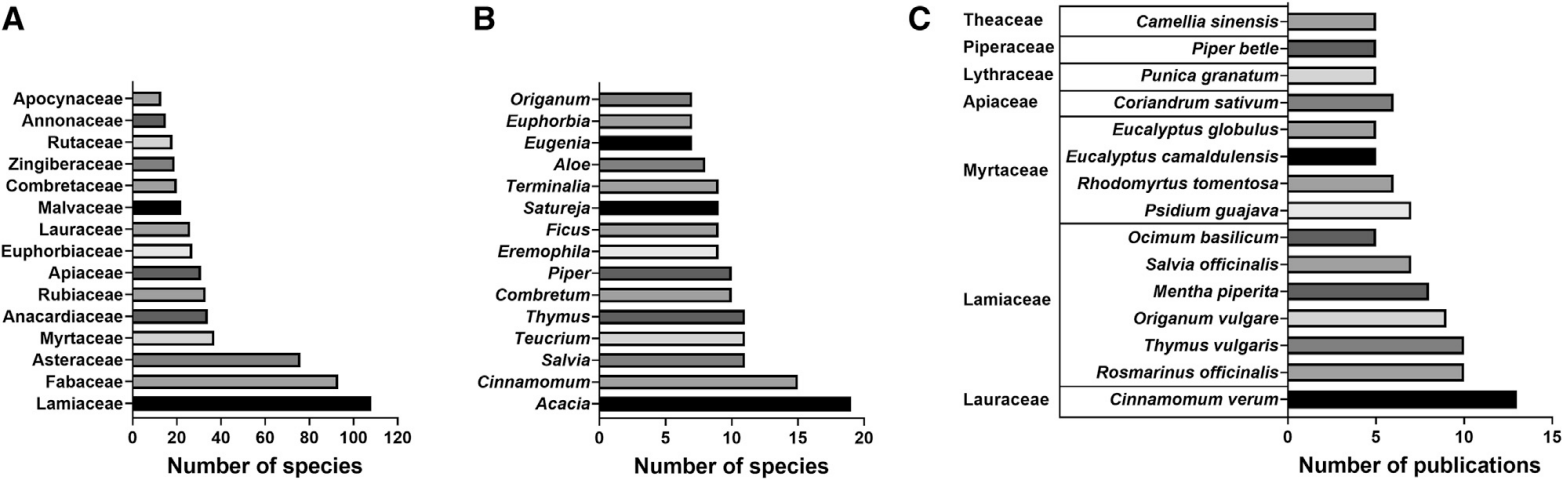
Most represented **(A)** families and **(B)** genera by number of species investigated for antibacterial activity in study range and **(C)** most studied plant species by number of publications, grouped by family.

A total of 958 plant species were investigated for antibacterial activity, representing approximately 0.3% of roughly 308,312 known vascular plant species (Christenhusz and Byng, 2016). Species studied came from 142 families and 562 genera, representing 30% of the 466 known vascular plant families. Lamiaceae (n = 108 species), Fabaceae (n = 93), Asteraceae (n = 76), Myrtaceae (n = 37) and Anacardiaceae (n = 33) were the top five families according to the number of species investigated (Figure 3A). All of these families, except for Anacardiaceae, belong to the top 10 largest botanical families worldwide with a range of 5,900 to 25,040 known species (Stevens, 2001). *Acacia* (n = 19 species), *Cinnamomum* (n = 15), *Salvia* (n = 11), *Teucrium* (n = 11) and *Thymus* (n = 11) were the five genera with the most species investigated for antibacterial activity (Figure 3B). Of the 958 plant species, 16 were studied in at least five different publications, among which *Cinnamomum verum* J. Presl, Lauraceae (n = 13 publications), *Rosmarinus officinalis* L., Lamiaceae (n = 10), *Thymus vulgaris* L., Lamiaceae (n = 10), *Origanum vulgare* L., Lamiaceae (n = 9) and *Mentha piperita* L., Lamiaceae (n = 8) were the five most studied (Figure 3C). The fact that all these five most studied species are common herbs or spices could indicate either the overlap of food and medicine in traditional medical systems or the easy accessibility of food plants for scientific study.

**FIGURE 4:**
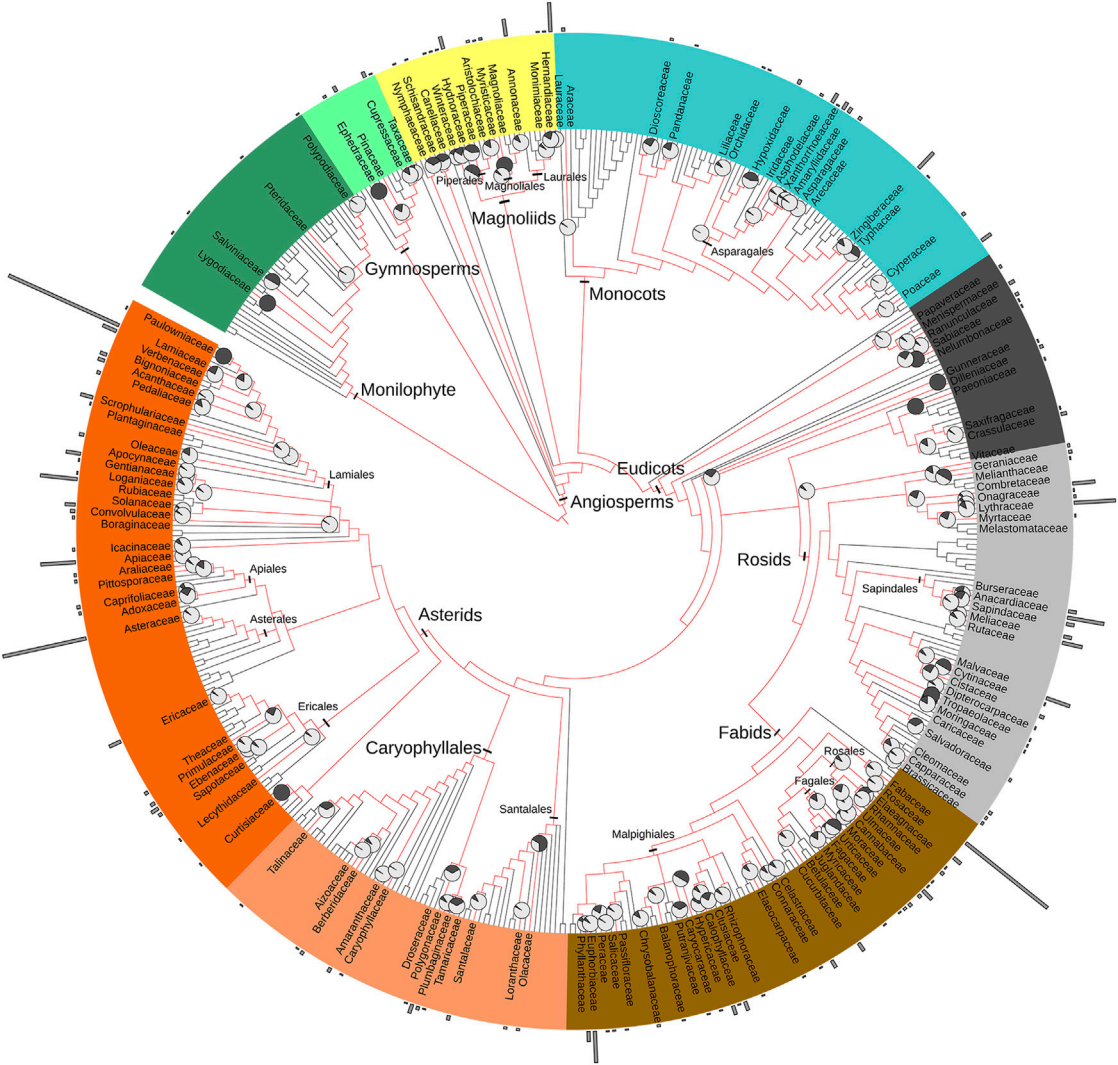
The phylogenetic distribution of plant species tested for antibacterial activity were mapped on the family-level maximally resolved complete euphyllophyte phylogenetic tree according to Angiosperm Phylogeny Group IV (Gastauer and Meira Neto, 2017). Family names are only given for those genera reported in this study; major clades are indicated in different colors. The length of the gray bars surrounding the phylogeny are according to the number of species studied in each family, and the proportion of genera studied in each family is displayed in pie charts on each family node. A high resolution version of this figure for enlarged viewing is available as Supplementary Material S3.

The 142 families tested for antibacterial activity are spread throughout the phylogenetic tree (Figure 4). Antibacterial activity has been reported in 1% of fern and fern allies (n = 4), 31% of gymnosperm families (n = 4) and 45% of early angiosperm and magnoliid families (n = 13). Only 0.2% of monocot families are reported with antibacterial activity (n = 16). Antibacterial activity is reported in 32% of eudicots families (n = 105), with over 21% of all species reported belonging to the asterid clade. Overall, the percentage of studied species was less than 2%, except in Combretaceae and Anacardiaceae (4%). Higher percentages of studied genera typically indicate smaller families with fewer genera. However, Anacardiaceae (25%), Lamiaceae (17%) and Myrtaceae (12%) show higher percentages of genera studied compared to the total number of genera per family (Figure 4). Widely distributed plant taxa are more represented throughout the tree than geographically restricted taxa, supporting the theory that widely distributed taxa have accumulated more genes for defensive secondary metabolites and are therefore good sources of antibacterial agents (Wink, 2003). However, overall some clades are well represented, while others are poorly investigated.

**FIGURE 5:**
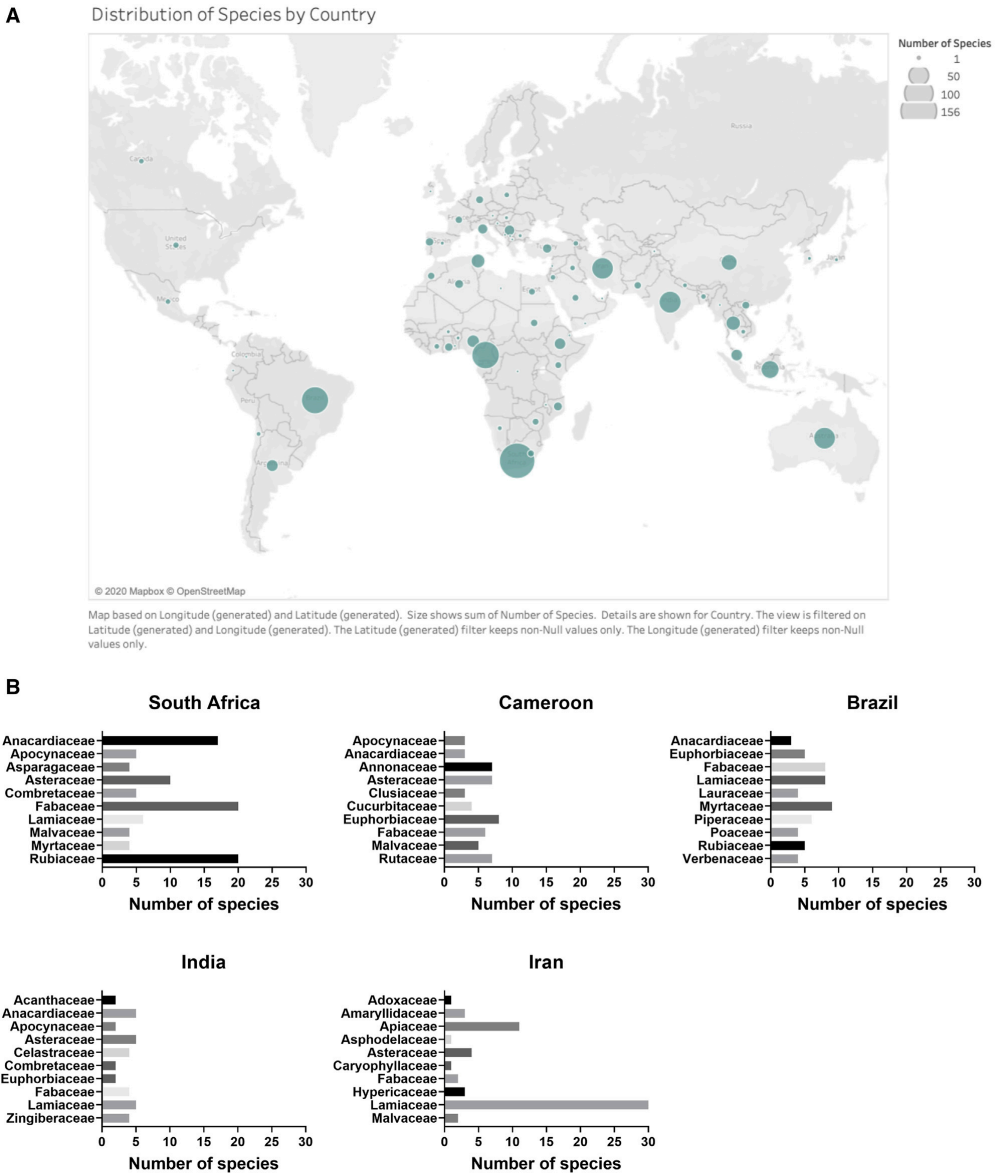
**(A)** Distribution of plant species by country. Country designation was determined by location of plant material collection. **(B)** Distribution of top 10 families within countries for the top five countries according to the number of species investigated.

The 958 plant species represented in the dataset were collected in seventy-three countries. South Africa, Cameroon, Brazil, India and Iran were the top five countries according to the number of species investigated (Figure 5).

### Other Descriptive Analyses

#### Ailment Categories and Plants in Clinical Trials

**FIGURE 6:**
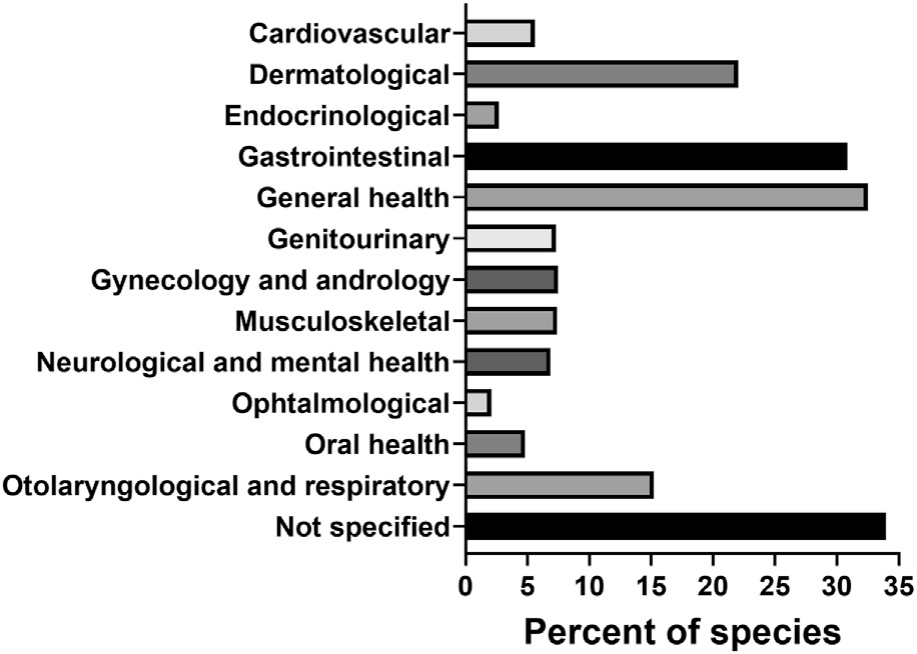
Categories of traditional medical use of antibacterial plant species.

The use of plants in traditional medicine was a guiding factor for the majority of the studies in this review. Out of 483 publications on antibacterial plant extracts, 349 (72.3%) discussed the ethnomedicinal uses of the plants being studied. Among categories of traditional medical use, these species were mostly applied in therapies supportive of general (32.5%), gastrointestinal (30.9%) and dermatological (22.0%) health (Figure 6).

Of the 958 plant species studied, 97 have been tested in clinical trials (Supplementary Material S2.2). This includes 72 species used in human food. Of the 258 clinical trials testing 59 plant species against infectious or inflammatory diseases, 78 trials (30%) were focused on oral health conditions such as periodontitis, gingivitis and dental caries, and 60 (23%) were focused on respiratory problems such as colds and rhinitis.

#### Plant Tissue and Extract Types

**FIGURE 7:**
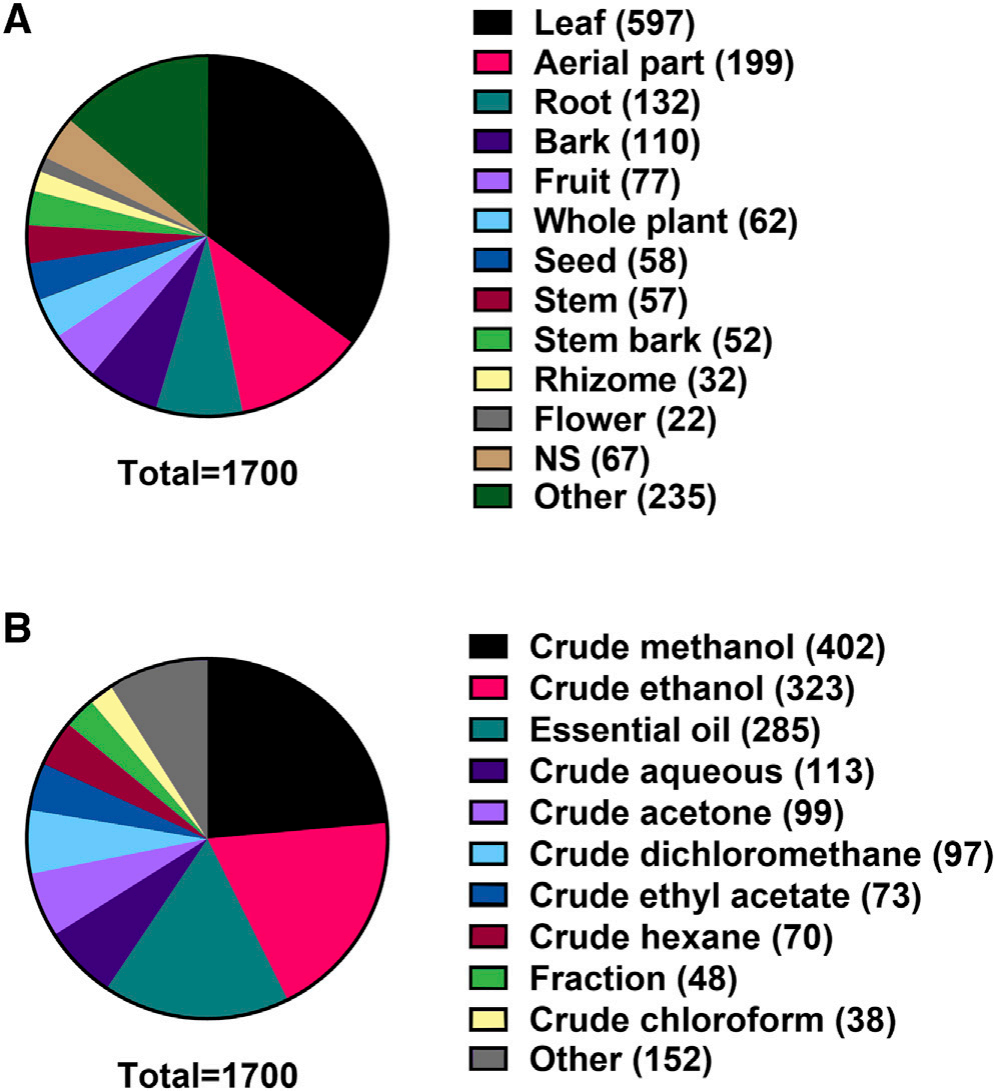
**(A)** Plant tissues extracted for investigation for antibacterial activity. **(B)** Method of extraction used to create extracts.

Of the 1,700 plant extracts investigated under our search parameters, 597 (35.1%) were from leaves and a further 199 (11.7%) were from aerial parts. The next most common plant tissues used were roots, bark and fruit (Figure 7A). Of the 1,700 extracts in the data set, crude methanolic extracts were the most represented with 402 extracts (23.7%), followed by crude ethanolic extracts with 323 extracts (19.0%) and EO with 285 extracts (16.8%) (Figure 7B).

#### Bacteria Targeted

**FIGURE 8:**
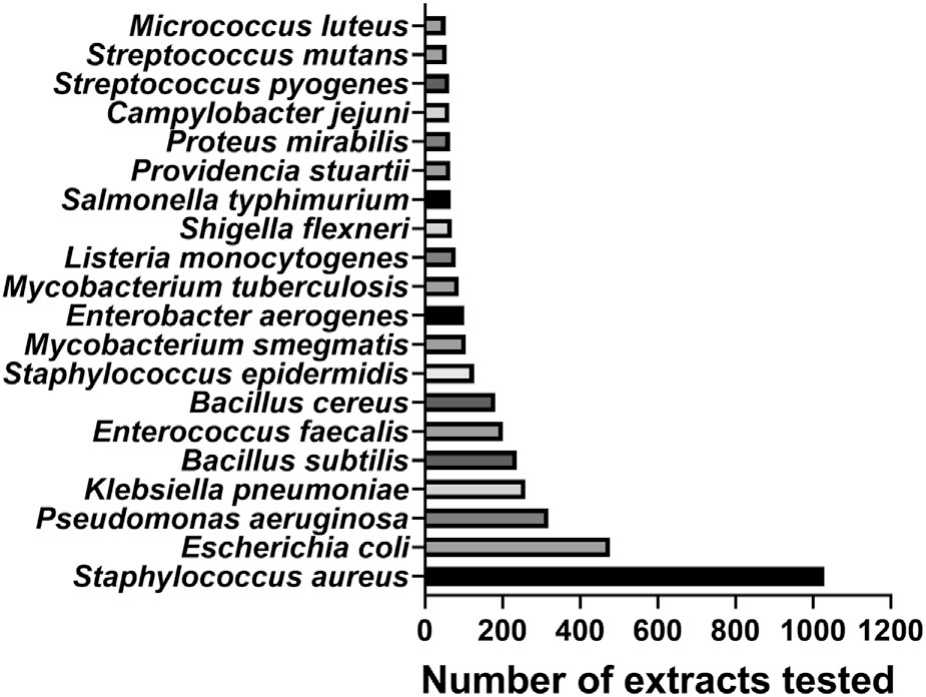
Top 20 bacterial species by number of extracts tested.

Of the 4,943 MIC values reported under our search criteria, the most targeted bacterial genus by far was *Staphylococcus* with 1,233 MIC values (25.0%), followed by *Bacillus* with 479 (9.7%) and *Escherichia* with 476 (9.6%). The most targeted species was *Staphylococcus aureus* (n = 1,028 extracts tested), followed by *Escherichia coli* (n = 476), *Pseudomonas aeruginosa* (n = 318) and *Klebsiella pneumoniae* (n = 259) (Figure 8). Four out of six “ESKAPE” pathogens—six pathogens with growing multidrug resistance and virulence: *Enterococcus faecium*, *Staphylococcus aureus*, *Klebsiella pneumoniae*, *Acinetobacter baumannii*, *Pseudomonas aeruginosa*, and *Enterobacter* spp. (Bassetti et al., 2013)—are represented in the 20 most targeted bacteria; less targeted in the study range are *A. baumannii* (n = 27) and *Enterococcus faecium* (n = 8). Of the urgent and serious bacterial threats listed in the U.S. Centers for Disease Control (CDC) 2019 Antibiotic Resistance Threats report (CDC, 2019), nine species are represented in the 20 most targeted bacteria (not necessarily resistant strains) and three species are less targeted: *Clostridioides difficile* (n = 2), *Neisseria gonorrhoeae* (n = 20) and *Streptococcus pneumoniae* (n = 27).

### Minimum Inhibitory Concentration Analyses

To determine quantitative differences in antibacterial activity associated with characteristics of the plant extracts in the study range, MIC values were grouped and analyzed by plant family, plant species, extraction type and plant tissue.

#### Minimum Inhibitory Concentration Analysis by Plant Families

**FIGURE 9:**
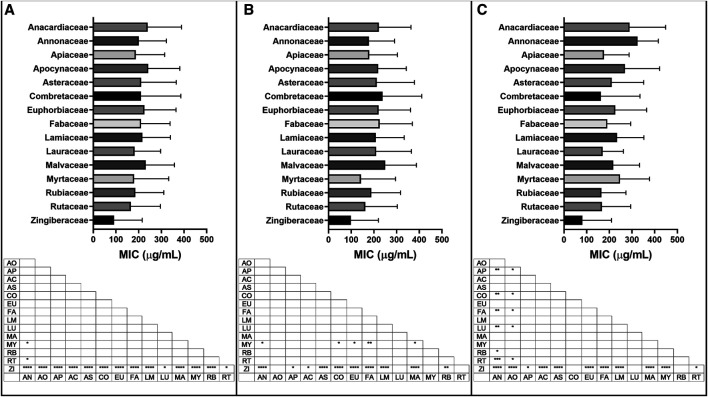
Mean and standard deviation of minimum inhibitory concentration (MIC) of extracts in the top fifteen families, with significant differences in MIC values (μg/mL) for **(A)** all bacteria; **(B)** Gram-positive bacteria and **(C)** Gram-negative bacteria reported. Botanical family code: AN: Anacardiaceae, AO: Annonaceae, AP: Apiaceae, AC: Apocynaceae, AS: Asteraceae, CO: Combretaceae, EU: Euphorbiaceae, FA: Fabaceae, LM: Lamiaceae, LU: Lauraceae, MA: Malvaceae, MY: Myrtaceae, RB: Rubiaceae, RT: Rutaceae, ZI: Zingiberaceae. *p* values: blank: not significant, *: *p* < 0.05, **: *p* < 0.01, ***p < 0.001, ****p < 0.0001.

The 15 most represented plant families by number of species in the study range (Anacardiaceae, Annonaceae, Apiaceae, Apocynaceae, Asteraceae, Combretaceae, Euphorbiaceae, Fabaceae, Lamiaceae, Lauraceae, Malvaceae, Myrtaceae, Rubiaceae, Rutaceae and Zingiberaceae) were further analyzed for their antibacterial activity. All data except those dealing with EOs were included in the initial analysis (3,498 MIC values), and a secondary analysis was carried out with Gram-positive bacteria only (1,944 MIC values) or Gram-negative bacteria only (1,541 MIC values) (Figure 9). EOs were analyzed separately due to the prevalence of a different unit (volume per volume μL/mL, instead of weight per volume μg/mL) in the reporting of their MIC values. Further descriptive statistics are available in Supplementary Material S2.3.

The Zingiberaceae, Rutaceae, Myrtaceae, Lauraceae and Rubiaceae families showed the lowest overall mean MICs of the top 15 families with values ranging from 92–185 μg/mL. Zingiberaceae extracts showed significantly better overall growth inhibitory activity than each of the other top 15 families (*p* < 0.05).

#### Minimum Inhibitory Concentration by Extraction Types

**FIGURE 10:**
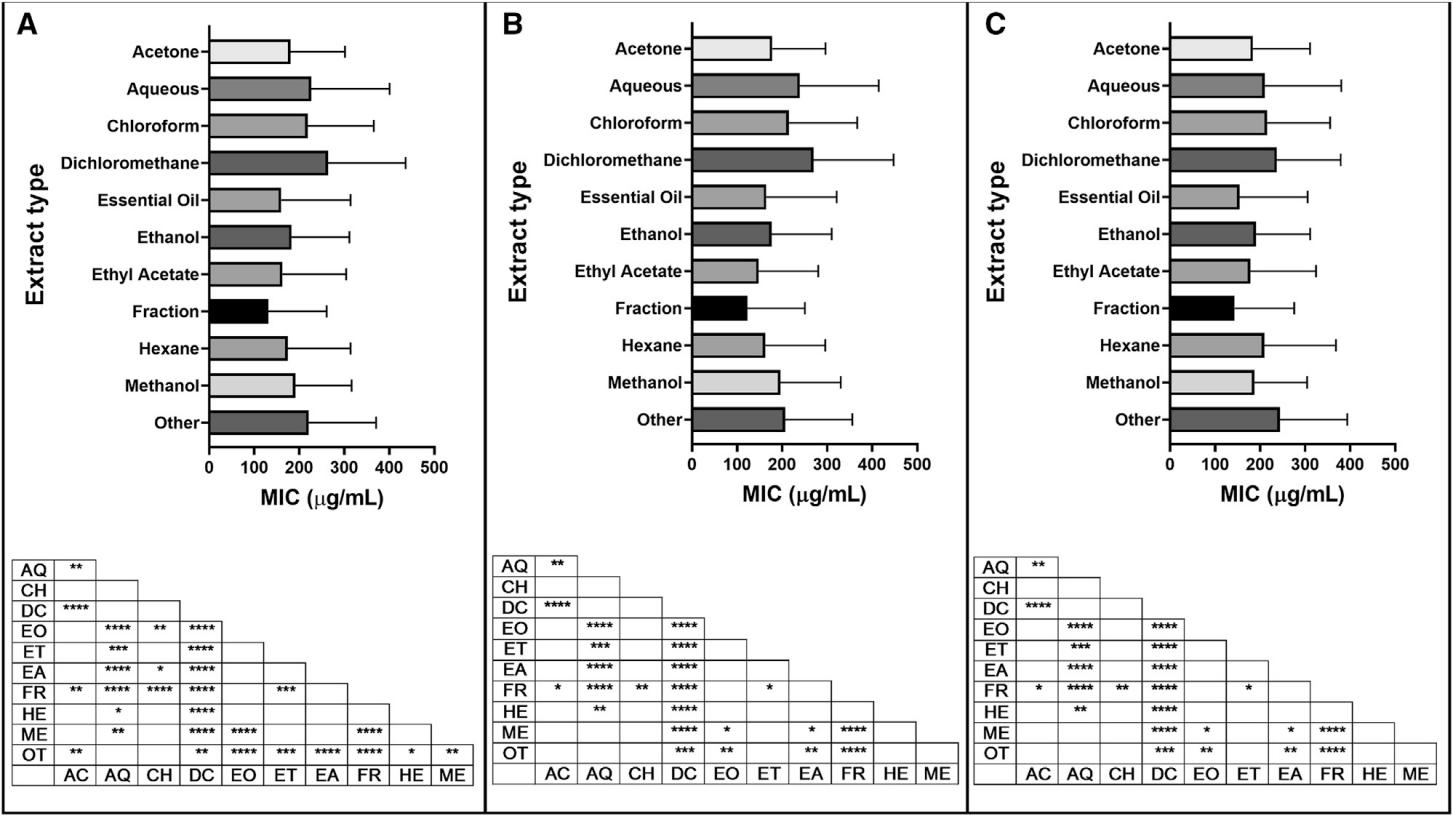
Minimum inhibitory concentration (MIC) of extracts reported by extraction type, with significant differences in MIC values for **(A)** all bacteria; **(B)** Gram-positive bacteria and **(C)** Gram-negative bacteria reported. Extraction code: AC: acetone, AQ: aqueous, CH: chloroform, DC: dichloromethane, EO: essential oil, ET: ethanol, EA: ethyl acetate, FR: fraction, HE: hexane, ME: methanol, NS: not specified, OT: other. *p* value: blank: not significant, *: *p* < 0.05, **: *p* < 0.01, ***p < 0.001, ****p < 0.0001.

From a total of 34 extraction types, the ten most represented types (methanol, ethanol, EO, aqueous, acetone, dichloromethane, ethyl acetate, hexane, fraction and chloroform) were further analyzed for their overall antibacterial activity. MIC data of plants extracted with different solvents were analyzed in three ways: looking at activities against all types of bacteria, Gram-positive bacteria or Gram-negative bacteria (Supplementary Material S2.4). Among solvents used for extraction, ethyl acetate showed the best (lowest) mean MICs, while dichloromethane extracts were the highest (Figure 10). Overall, fractions of plant extracts showed the best (lowest) mean MICs demonstrating the efficiency of the fractionation processes and its importance toward the discovery of new antibacterial agents.

#### Minimum Inhibitory Concentration by Plant Tissue

**FIGURE 11:**
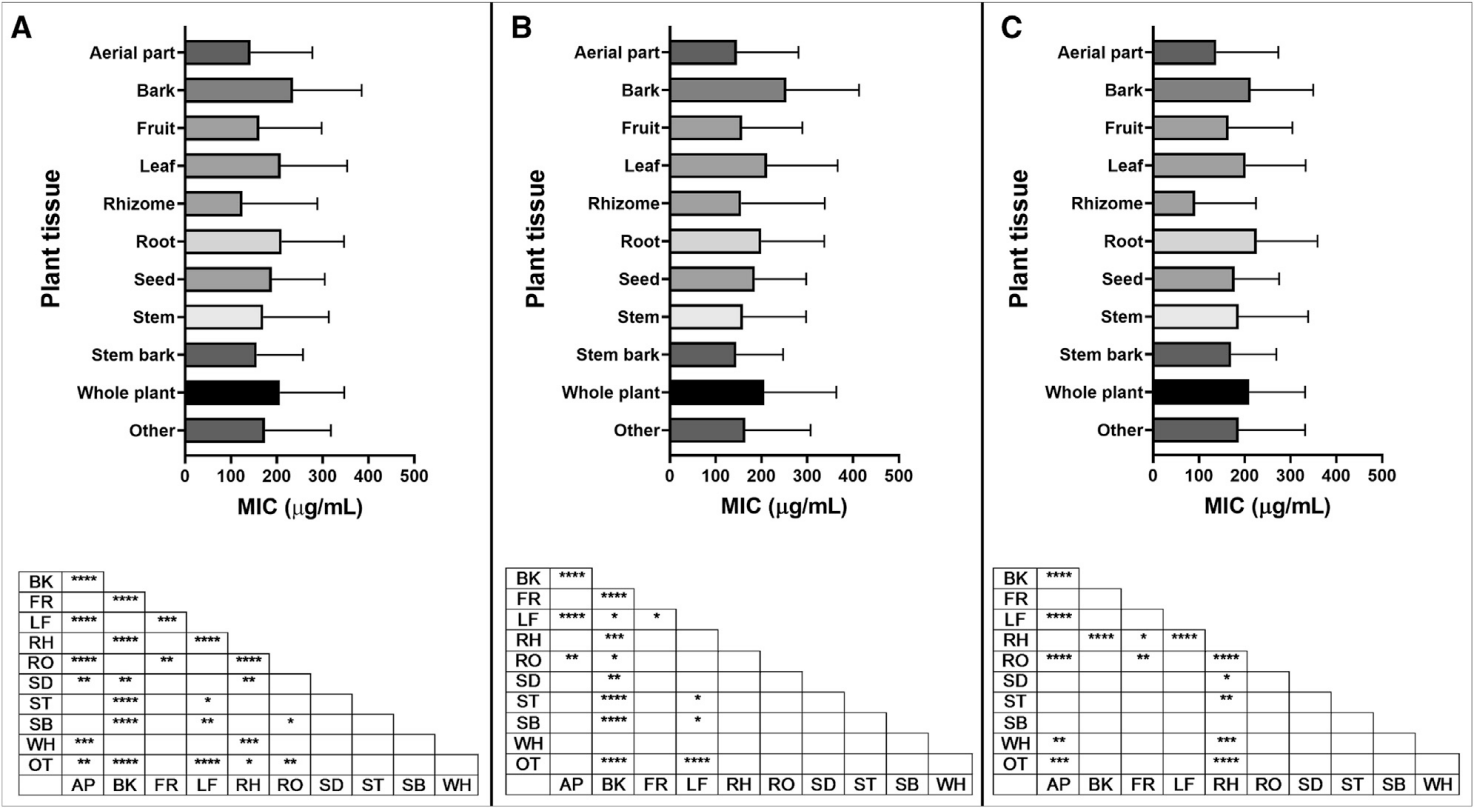
Minimum inhibitory concentration (MIC) of extracts reported in the top ten plant tissues, with significant differences in MIC values for **(A)** all bacteria; **(B)** Gram-positive bacteria and **(C)** Gram-negative bacteria reported. Plant tissue code: AP: aerial part, BK: bark, FR: fruit, LF: leaf, RH: rhizome, RO: root, SD: seed, ST: stem, SB: stem bark, WH: whole plant, OT: other. *p* value: blank: not significant, *: *p* < 0.05, **: *p* < 0.01, ***: *p* < 0.001, ****: *p* < 0.0001.

Of the 88 different plant tissues or groups of tissues studied, the ten most represented (leaf, aerial part, root, bark, fruit, whole plant, seed, stem, stem bark, rhizome) were selected for further analysis. MIC data of plant extracts from different plant tissues were analyzed in three ways: looking at all types of bacteria, Gram-positive bacteria, or Gram-negative bacteria (Figure 11A; Supplementary Material S2.5). For Gram-positive bacteria, mean MIC values from bark extracts were statistically higher than those from aerial part, fruit, leaf, rhizome, root, seed, stem and stem bark extracts (Figure 11B). For Gram-negative bacteria, rhizome extracts showed significantly lower mean MIC values than bark, fruit, leaf, root, seed, stem and whole plant extracts (Figure 11C). This can be explained by the low mean MIC found for species belonging to the Zingiberaceae family, which accounts for 91% (50/55) of the MIC values for rhizome extracts.

#### Minimum Inhibitory Concentration by Species (Crude Extracts)

**FIGURE 12:**
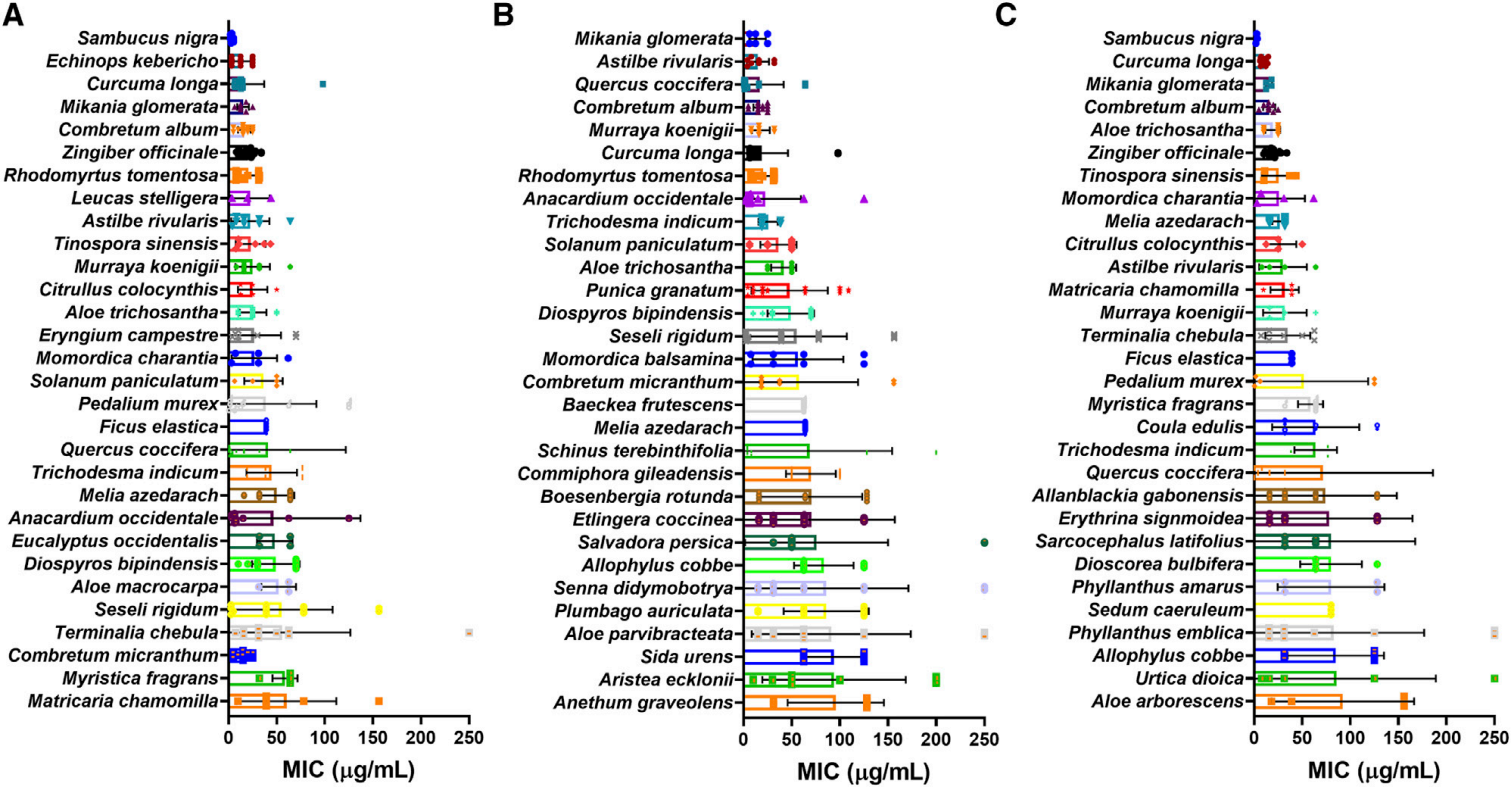
MIC values of plant crude extracts with the lowest mean MIC values for **(A)** all bacteria; **(B)** Gram-positive bacteria and **(C)** Gram-negative bacteria reported.

Of the 739 plant species with extract MIC values measured in weight by volume (µg/mL) and not extracted as EOs, 331 were tested against at least three different bacteria strains from all types, 158 were tested against at least three different Gram-positive bacteria and 155 were tested against at least three different Gram-negative bacteria. *Sambucus nigra* L. (Adoxaceae), *Echinops kebericho* Mesfin (Asteraceae), *Mikania glomerata* Spreng. (Asteraceae), *Curcuma longa* L. (Zingiberaceae) and *Combretum album* Pers., (Combretaceae) extracts showed the best overall mean MIC values with values ranging from 3.5–16 μg/mL (Figure 12).

#### Minimum Inhibitory Concentration by Species (Essential Oils)

**FIGURE 13:**
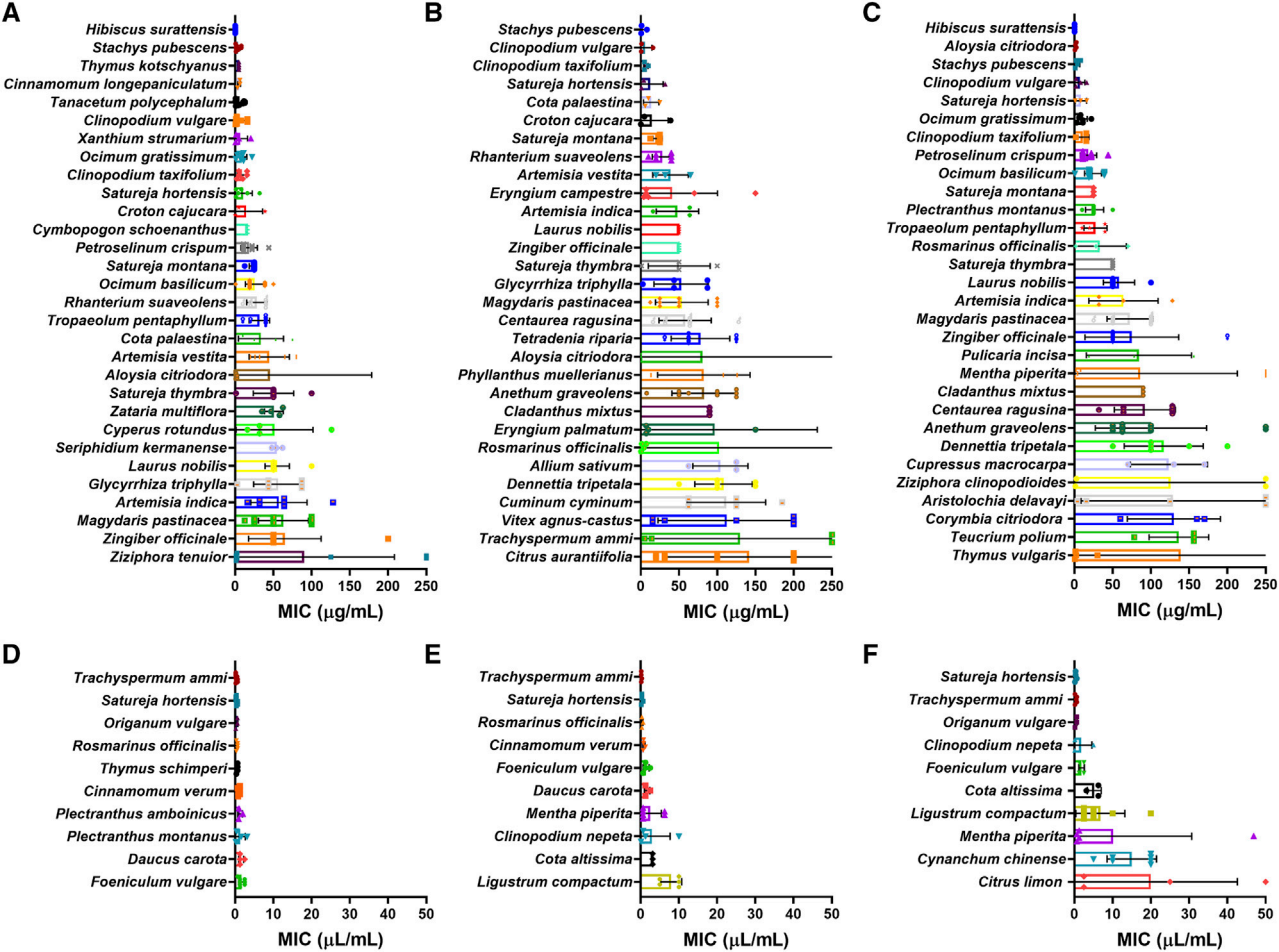
MIC of plant essential oils with lowest mean MIC (µg/mL) for **(A)** all bacteria; **(B)** Gram-positive bacteria and **(C)** Gram-negative bacteria and lowest mean MIC (µL/mL) for **(D)** all bacteria; **(E)** Gram-positive bacteria and **(F)** Gram-negative bacteria. *: *p* < 0.05.

MIC values for species extracted as EOs and tested against more than three species of bacteria were selected for analysis, with values in weight per volume (µg/mL) and volume per volume (µL/mL) being treated separately. Of the 117 plant species extracted as EOs with weight per volume MICs (Figures 13A–C), *Hibiscus surattensis* L. (Malvaceae) showed the lowest MIC overall and for Gram-negative bacteria (0.09 μg/mL in both cases). *Stachys pubescens* Ten. (Lamiaceae) had the lowest MIC for Gram-positive bacteria (3.17 μg/mL). Of the 22 plant species extracted as EOs with volume per volume MICs (Figures 13D–F), *Trachyspermum ammi* (L.) Sprague showed the lowest MIC overall and for Gram-positive bacteria (0.32 and 0.20 μL/mL respectively). *Satureja hortensis* L. had the lowest MIC for Gram-negative bacteria (0.35 μL/mL).

## Discussion

### Phylogenetic Distribution of Antibacterial Plants

During the process of evolution, plants coevolved with other organisms to attract pollinators and seed dispersers, or to defend themselves from microbes and herbivores. Chemical diversification of secondary metabolites in different plant lineages is not random, but rather is precisely correlated to this adaptive radiation. The correlation between phylogeny and plant chemistry can offer a predictive approach to drug discovery, enabling more efficient selection of plants for chemical mining. As such, we have examined major clades and address the extent to which they were covered by studies on antibacterial activity.

Examination of the major clades in the phylogenetic tree reveals that the magnoliid clade has been reasonably well studied. Fifty percent of the well-known magnoliid families were studied and 62 species were reported to have antibacterial activity (Figure 4). Magnoliids are early angiosperms, comprising four orders, 18 families, and approximately 10,000 species that face relatively high pressure from herbivory (Turcotte et al., 2014; Mawalagedera et al., 2019); thus this group is characterized by unique aromatic compounds such as sesquiterpenes, isoflavonoids, and benzylisoquinoline alkaloids (e.g., aporphines, aristolochic acids, and bis-benzylisoquinolines) (Aminimoghadamfarouj et al., 2011; Courtois et al., 2016). This suggests that other members in the magnoliids clade, including Piperales, Magnoliales and Laurales could be good candidates for chemical studies. However, less than 1% of species have been examined in each order. Lauraceae, Myristicaceae and Annonaceae are large families in the magnoliid clade; nevertheless, only 42 species (Lauraceae = 26; Annonaceae = 15; Myristicaceae = 1) were reported for antibacterial activity studies under our search criteria, representing ∼0.7% of known species in this clade.

Monocots are the least studied group among angiosperms for antibacterial activity, with only 16 out of 80 monocot families evaluated. There are approximately 60,100 monocot species and only 69 (∼0.1%) were reported to have antibacterial activity. This is not a surprising result because monocots have crystalline calcium oxalate raphides (needle-shaped crystals) (Franceschi and Nakata, 2005) and other physical defensive mechanisms against herbivory that they depend upon rather than chemical defensive mechanisms.

Antibacterial activities were recorded in few of the families in the Basal Eudicots group—11 out of 32 families. Four out of seven families in the Ranunculales were reported to have antibacterial activity (Figure 4). Berberine and a great variety of benzylisoquinolines alkaloids are common in this Ranunculales clade. In total, there are approximately 4,500 species in the Papaveraceae, Menispermaceae, Berberidaceae and Ranunculaceae families, but only 13 species (∼0.3%) were recorded in our search, representing a large source of untapped potential chemical novelty in this group.

In the rosid clade, Sapindales and Myrtales have been studied for antibacterial activity. Sapindales is characterized by gums and resins. Five out of nine families in this group are reported to have high antibacterial activity (Figure 4). The remaining four families are taxonomically recently separated, lesser known small families that are restricted to certain geographic regions. However, these should also be considered for future evaluation. For example, Simaroubaceae is a lesser studied small family (∼110 species) mainly restricted to the tropics, yet it is chemically unique, having distinctive secondary metabolites like quassinoids (a group of natural products classified as triterpenoids) that are not found elsewhere in the order (Waterman, 1993; Curcino Vieira and Braz-Filho, 2006). Out of 6,570 known species in the rosid clade, only 75 (∼1.1%) have been reported for their antibacterial activity.

Asterid is the youngest and largest clade of all the angiosperm phylogeny (Wikström et al., 2015), with Lamiales, Apiales, Asterales and Ericales as the prominent plant orders. Among those, Asterales is the largest order of all, containing about 13.6% eudicot diversity (Magallon et al., 1999). However, plant species in Asterales were not well represented in antibacterial activity studies. Out of 11 Asterales plant families, Asteraceae is the only family recorded to have antibacterial activity. It is the largest angiosperm plant family with about 25,040 known species, but only 76 species (0.3%) were recorded to have activity under our search parameters. Asteraceae has terpenoids, coumarins, polyacetylenes, and flavonoids with potential therapeutic agents (Panda et al., 2019). Despite the discovery of several important secondary metabolites such as artemisinin and silibinin, little attention has been paid to the other active compounds present in Asteraceae.

Other families in the Asterales are smaller, lesser known and restricted to certain geographical areas. However, Campanulaceae is another family in the Asterales that is distributed worldwide and yet no species in that family were reported in our data set. This may be due to the chemical evolution of the clade: iridoids, characteristic compounds in the asterids clade, have a bitter taste and are emetics for vertebrates. Iridoids play a major role in herbivore preference in asterids to attract or mostly to deter as well as to protect the plant against bacterial and fungal pathogens (Dobler et al., 2011). Among asterids, Asterales is one of the orders that does not have iridoids. Ericales and Cornales were the earliest to diversify and are known to have both ellagic acid and iridoid type compounds. Both orders have a number of species with higher antibacterial activity.

Lamiales is the next largest order in the asterids clade, and it contains about 12% eudicot diversity. Out of 25 families in the Lamiales order, all the well-known families are recorded to have antibacterial activity. The remaining 16 families that were not studied are small families, collectively containing fewer than 100 species and mostly restricted to certain geographic areas. Lamiales have about 1,050 genera and approximately 23,700 known species; of these, 0.5% were reported for antibacterial activity in our search. The remaining species within this order have a high potential to exhibit antibacterial activity and should be considered in future studies.

Caryophyllales is a sister to asterids and is characterized by having succulents and carnivorous plants. Out of 38 families, nine were studied for antibacterial activity (Figure 4). Various isoflavonoids and other unique compounds are scattered in this group: flavonol sulfates occur in Plumbaginaceae, Polygonaceae and Amaranthaceae, and sulfated betalains in Phytolaccaceae (Mackova et al., 2006). Naphthoquinones, which are considered to be potential antifungal drugs, are also produced by many plants that belong to the Caryophyllales families, including Plumbaginaceae, Droseraceae, Nepenthaceae, Dioncophyllaceae and Polygonaceae (Culham and Gornall, 1994; Rischer et al., 2002; Kovácik and Repcák, 2006). Other than Amaranthaceae, Caryophyllaceae and Cactaceae, most families of Caryophyllales are monotypic and restricted to tropical and subtropical regions. However, those families also contain various unique compounds. For example, the Dioncophyllaceae and Ancistrocladaceae families, which consist of lianas found in the tropical rainforests of West Africa and Southeastern Asia, contain naphthylisoquinoline alkaloids, which are exclusively found in these two families and are worth considering in future antibiotic research (Ibrahim and Mohamed, 2015).

The Santalales is also a small sister group to asterids. Loranthaceae and Santalaceae are the best known families in this clade, and they are distributed worldwide. Other families are very small with few genera and species. Loranthaceae and Santalaceae are both reported for antibiotic activities (Kurekci et al., 2012; van Vuuren et al., 2015; Faboro et al., 2016; Voukeng et al., 2017; Thielmann et al., 2019). However, other families in this clade are not as well studied for unique chemistry and bioactivity (Kubitzki, 2013).

The Fabales, Fagales, Rosales and Malpighiales are the major orders in the fabid clade. Many families in this clade are characterized by cyanogenic glycosides and ellagic acids. Fabales is one of the biggest orders of all eudicots, containing about 9.6% of eudicot species (Magallon et al., 1999). Fabaceae is the largest family with about 19,580 species. While 93 individual species of Fabaceae were reported for antibacterial activities under our study criteria, this represents a relatively small percentage (0.5%) of the total diversity in this family.

Gymnosperms and ferns were poorly investigated. Out of 37 fern families, only four were recorded with antibacterial activity; in each of these four families, a single species was studied. Out of 13 gymnosperm families, four were reported to have antibacterial activity and fewer than five species were studied in richly biodiverse families such as Cupressaceae, Ephedraceae and Pinaceae. Interestingly, some gymnosperm families with known use in traditional medicines such as the resin-rich Araucariaceae and Podocarpaceae were not reported in this data set for antibacterial activity; this may indicate a need for the application of standardized methods for the future antimicrobial assessment of these species.

### Geographic Distribution of Antibacterial Plants

South Africa, Cameroon, Brazil, India, and Iran were the top five countries by source of plants examined according to the number of species investigated. There are several factors that may explain the large number of plant species in high-quality antibacterial studies in these top five countries; a strong combination of plant diversity, ethnobotanical tradition and scientific equipment and training is needed to study plant antibacterials on this scale. Additionally, the dataset is small enough that the contributions of individual research groups can be clearly seen.

### Influence of Plant Tissue and Extract Types on Minimum Inhibitory Concentration Values

The prevalence of leaves and aerial parts in investigations of plant antibacterials could be due to the ease of collecting these tissues without killing the source plant, allowing for sustainable use. Plants use secondary metabolites to interact with bacteria to various degrees in all of their tissues, promoting beneficial organisms and suppressing pathogens, particularly in the rhizosphere (Berendsen et al., 2012) but also in aerial tissues such as leaves (Karamanoli et al., 2005). However, the profile of secondary metabolites varies between plant tissues, and certain metabolites are concentrated in specific tissues (Kim et al., 2002). In general, annual plants contain high levels of secondary metabolites in their reproductive parts and perennial plants contain high levels of secondary metabolites in their roots, rhizomes and bark (Wink, 2010). The low mean MIC of aerial part extracts—typically from herbaceous species—may therefore be due to the richness of secondary metabolites in the reproductive parts (Jones and Kinghorn, 2005).

Regarding the choice of solvents for extractions, the prevalence of alcohol parallels its use in many traditional medicine systems. Solvent choice is based on its ability to diffuse into the plant cells, dissolve the secondary compounds and diffuse back out of the cell. For example, water swells the plant cells, allowing for a more complete penetration by the solvent. The wide availability of methanol and ethanol may also account for their prevalent use as extraction solvents.

Water and organic solvents such as methanol, ethanol, acetonitrile, acetone, hexane and dichloromethane are commonly employed in the extraction of bioactive compounds from plants in laboratory studies. Choice of extraction solvent is important because extract yields and resulting biological activities of the plant materials are strongly dependent on the nature of extracting solvent; this is due to the presence of different bioactive compounds of varied chemical characteristics and polarities that may or may not be soluble in a particular solvent (Sultana et al., 2009; Do et al., 2014; Metrouh-Amir et al., 2015). For example, we noted that dichloromethane exhibited the worst (highest) MICs of any solvents evaluated; hence, while it is not the most suitable for the extraction of antibacterials from plants, it is still very useful for prefractionation.

On the other hand, significant MIC values observed in essential oils (EOs) resemble the data reported in several studies (Kalemba and Kunicka, 2003; Burt, 2004; Bakkali et al., 2008), where the ability of these substances to hamper the growth of a diverse range of human pathogens is described. EOs are concentrated hydrophobic liquids extracted from plants. They contain volatile compounds and are “essential” inasmuch as they contain the “essence” of the plant’s fragrance. In general, the chemical composition of essential oils is relatively complex, with about 20–60 different bioactive components and only two to three major components at a fairly high concentrations (20–70%) compared to other components present in trace amounts. Their most common constituents are terpenes, aromatic and aliphatic compounds (especially alcohols, esters, ethers, aldehydes, ketones, lactones, phenols and phenol ethers) (Burt, 2004; Bakkali et al., 2008). The phenolic components are chiefly responsible for the antibacterial properties of EOs (Cosentino et al., 1999); however, there is some evidence that minor components have a critical part to play in antibacterial activity, possibly by producing a synergistic effect between other components (Paster et al., 1995; Marino et al., 2001), while some exert no activity on their own (Burt, 2004; Tariq et al., 2019).

### Taxonomic Distribution of Antibacterial Plants

**FIGURE 14:**
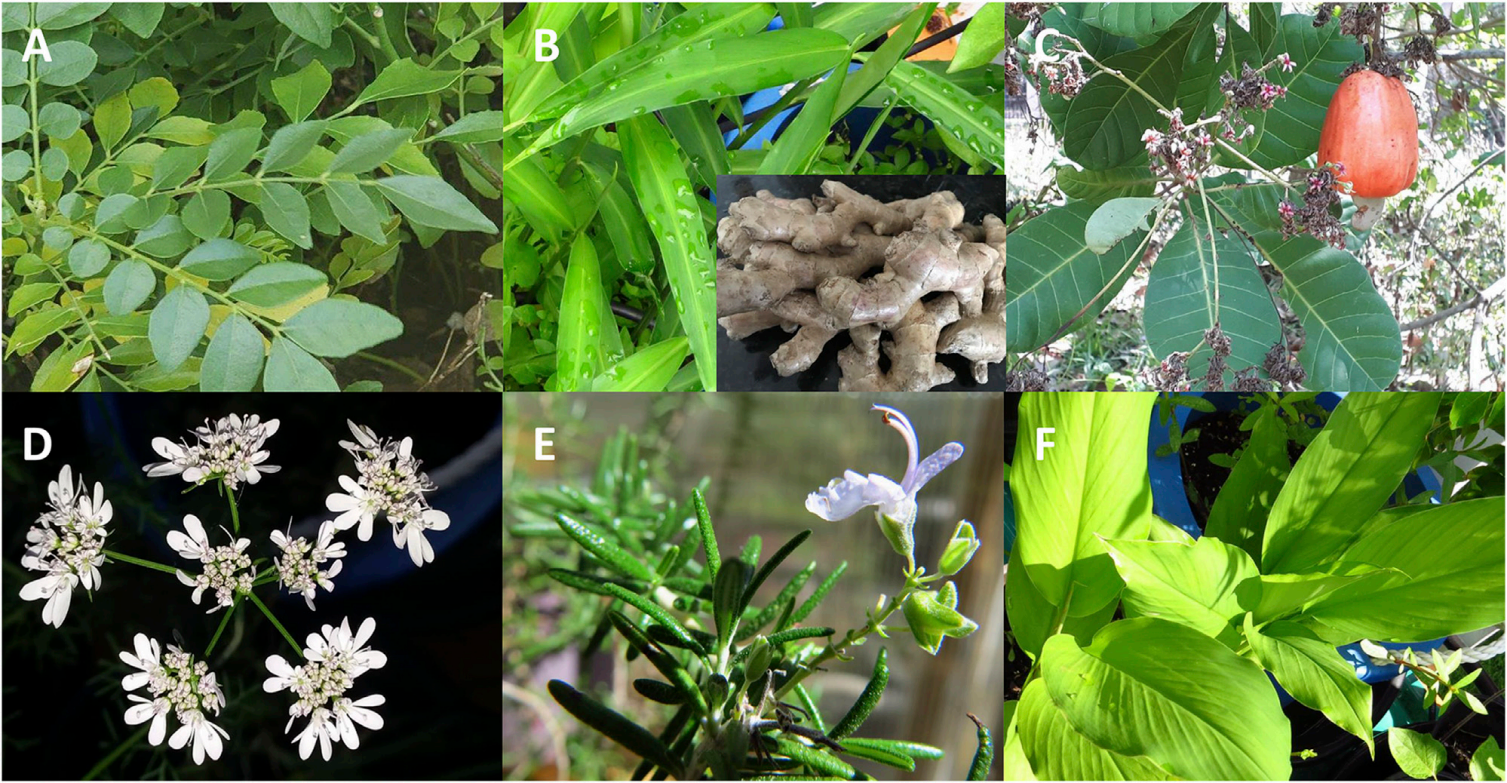
Select examples of plant species with antibacterial activity: **(A)** Curry leaf plant (*Murraya koenigii*, Rutaceae) is a tree native to the Indian subcontinent which showed one of the best mean MICs against Gram-positive bacteria, **(B)** Ginger (*Zingiber officinale*, Zingiberaceae) is an herb native to southeastern Asia and is one of the most active species. **(C)** Cashew (*Anacardium occidentale*, Anacardiaceae) is a tropical evergreen tree. It shows one of the best MIC values as a plant extract, and the Anacardiaceae family has the highest percentage of plants species studied for antibacterial activity, **(D)** Coriander (*Coriandrum sativum*, Apiaceae) is an herbaceous plant native to western Asia and southern Europe. Coriander seed EO shows one of the highest activities in the Apiaceae family, and this species is one the most investigated for antibacterial activity, **(E)** Rosemary (*Rosmarinus officinalis*, Lamiaceae) is a perennial herb native to the Mediterranean region. It is one of the five genera with the most species investigated for antibacterial activity. **(F)** Turmeric (*Curcuma longa*, Zingiberaceae) is an herbaceous plant native to south-east Asia. It is one of the five species that showed the best overall mean MIC values for Gram-negative bacteria.

**FIGURE 15:**
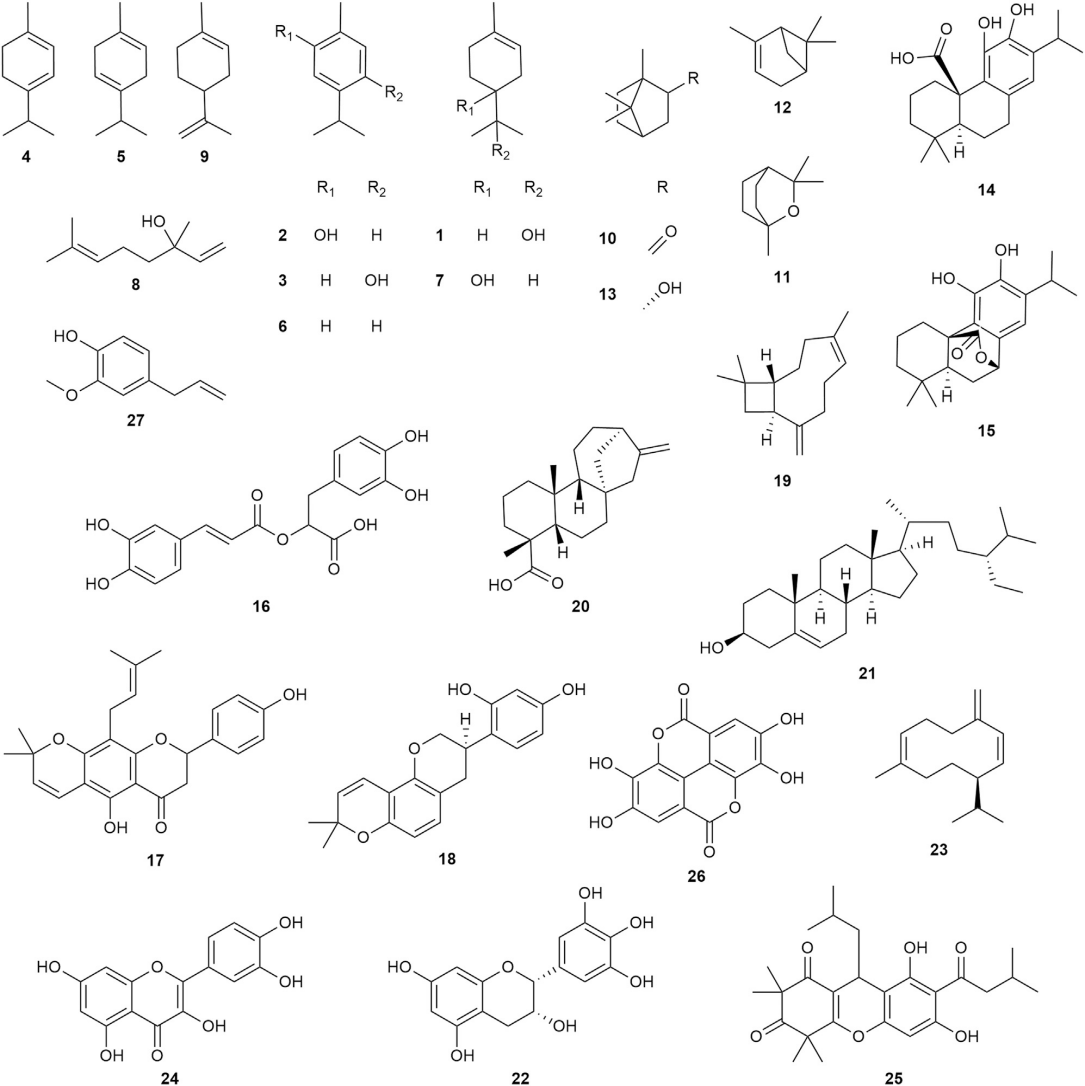
Representative chemical structures of compounds discussed in this review; α-terpineol (**1**), carvacrol (**2**), thymol (**3**), α-terpinene (**4**), γ-terpinene (**5**), *p*-cymene (**6**), terpinen-4-ol (**7**), linalool (**8**), limonene (**9**), camphor (**10**), eucalyptol (**11**), α-pinene (**12**), borneol (**13**), carnosic acid (**14**), carnosol (**15**), rosmarinic acid (**16**), lupinifolin (**17**), glabridin (**18**), β-caryophyllene (**19**), *ent*-kaurenoic acid (**20**), β-sitosterol (**21**), epigallocatechin (**22**), germacrene D (**23**), quercetin (**24**), rhodomyrtone (**25**), ellagic acid (**26**), and eugenol (**27**).

In the following section, we highlight 44 plant species notable for their prominence in traditional medicine or for potent antibacterial activity, selecting representatives from each of the top seven families by number of species studied and ordered as such below. We have elaborated on an additional 26 species from the next nine top families in Supplementary Material S2.6. Refer to Supplementary Material S1 to examine the full dataset with species, extraction tissue and type, as well as specific MICs for reported pathogens. Select examples of bioactive species are depicted in Figure 14 and antibacterial compounds in Figure 15. In most cases, additional *in vitro*, *in vivo* and clinical research is still needed to confirm efficacy and safety, as well as to identify the responsible compounds and their respective mechanisms of action.

#### Lamiaceae

Of the 108 species from the Lamiaceae family that exhibited antibacterial activity, the ten most active species were *Origanum vulgare* L., *Origanum majorana* L., *Thymus zygis* L., *Rosmarinus officinalis* L., *Thymus vulgaris* L., *Clinopodium taxifolium* (Kunth) Govaerts, *Clinopodium vulgare* L., *Mentha* x *piperita* L., *Stachys pubescens* Ten. and *Ocimum basilicum* L. All were extracted as EOs and seven are native to the Mediterranean. *Thymus*, *Origanum* and *Clinopodium* species contain similar compounds and are especially rich in monoterpenes such as α-terpineol (**1**), carvacrol (**2**), thymol (**3**), α-terpinene (**4**), γ-terpinene (**5**) and *p*-cymene (**6**), which synergize with each other and enhance the antimicrobial efficacy of the complex extract mixture compared to single compounds alone (Ahmad et al., 2014a).

*Origanum vulgare* (oregano) is an aromatic herb found in Asia, Europe and northern Africa. It is used to treat respiratory problems, digestive disorders, dermatological conditions and various other inflammatory and infectious disorders (Polat and Satıl, 2012; Ahmad et al., 2014b). EOs exhibited MICs ranging from 0.03–100 μg/mL against *Listeria monocytogenes*, *Pseudomonas aeruginosa*, *Staphylococcus aureus*, *Streptococcus pyogenes*, *Escherichia coli* and *Acinetobacter baumannii* (Becerril et al., 2012; Santos et al., 2017; Helal et al., 2019; Thielmann et al., 2019). Compounds **2** and **3** are the two main constituents responsible for the antibacterial activity of oregano EO (Bozin et al., 2006). *In vivo* studies using mouse burn wounds infected with methicillin-resistant *Staphylococcus aureus* (MRSA) showed a significant reduction of bacterial load (Lu et al., 2018) and an oregano-based ointment has been developed to target bacterial species including MRSA (Eng and Norman, 2010).

*Origanum majorana* (sweet marjoram) is a perennial herb native to the Mediterranean countries. It is traditionally used to treat asthma, indigestion, headache, insomnia and rheumatism in traditional medicine (Della et al., 2006). EOs of the flowering aerial parts inhibit *E. coli* (MIC = 0.19 μg/mL) and *S. aureus* (MIC = 6.25 μL/mL) (Vaillancourt et al., 2018; Lagha et al., 2019). The antibacterial activity of marjoram EO has been associated with the high proportion of monoterpenes such as **1**, **4**, **5**, **6**, and terpinen-4-ol (**7**) and in a lesser manner with the presence of **2** and **3** (Hajlaoui et al., 2016).

*Thymus vulgaris* (thyme) is an aromatic shrub from the Mediterranean. The aerial parts are used in the traditional treatment of respiratory problems such as cough, bronchitis, laryngitis and sore throat (Fani and Kohanteb, 2017). EOs exhibited MICs ranging from 0.3–30 μg/mL against *Bacillus cereus, E. coli, L. monocytogenes, Salmonella typhimurium* and *Legionella pneumophila*, and 0.78 μL/mL against *S. aureus* (Aliakbarlu and Shameli, 2013; Chaftar et al., 2015; Ghrairi and Hani, 2015; Vaillancourt et al., 2018). The main constituents of thyme EO are **2** and **3**, as well as the active monoterpene compounds **5** and **6** (Fournomiti et al., 2015).

*Thymus zygis* (red thyme) is an endemic species in the Iberian Peninsula. An EO of the flowering herb exhibited MICs of 100 μg/mL against *S. aureus* and a range of 0.19–400 μg/mL against three strains of *E. coli* (Lagha et al., 2019; Thielmann et al., 2019). There are different chemotypes rich in **3** and linalool (**8**) (Rota et al., 2008). The variation of the antibacterial activity within the plant species could be explained by the presence of different chemotypes.

*Rosmarinus officinalis* (rosemary, Figure 14E) is a shrub native to the Mediterranean traditionally used as a pulmonary antiseptic, choleretic, stomachic and antispasmodic (Nejad et al., 2013). It was also reported to treat urinary tract infections, leishmaniasis, other microbial infections and inflammation (Jamila and Mostafa, 2014). EOs of the aerial parts exhibited MICs ranging from 0.3–1.6 μg/mL in *Staphylococcus epidermidis*, *S. aureus* and *E. coli*, and a range of 7–70 μg/mL in *L. monocytogenes*, *P. aeruginosa* and *Legionella pneumophila* (Chaftar et al., 2015; Jardak et al., 2017; Santos et al., 2017; Lagha et al., 2019). Limonene (**9**), camphor (**10**), eucalyptol (**11**), α-pinene (**12**), *Z*-linalool oxide and borneol (**13**) are among the major EO constituents which are responsible for antibacterial activity (Bozin et al., 2007; Celiktas et al., 2007). Ethanol extracts also demonstrated MICs ranging from 70–350 μg/mL against *Staphylococcus saprophyticus*, *S. epidermidis*, *Enterococcus faecalis* and *P. aeruginosa* (Petrolini et al., 2013). The presence of diterpenes such as carnosic acid (**14**) and carnosol (**15**) as well as the phenolic rosmarinic acid (**16**) have been reported to be directly involved in the antibacterial activity (Jordán et al., 2012). A phytotherapeutic drug, Canephron, which contains *R. officinalis*, *Levisticum officinale* W.D.J.Koch (Apiaceae) and *Centaurium erythraea* Rafn (Gentianaceae), demonstrated safety and efficacy in the treatment of urinary tract infections in a meta-analysis of 17 clinical studies (Naber, 2013). Another clinical trial evaluating the efficacy of a mouthwash containing hydroalcoholic extracts of *Zingiber officinale* Roscoe (Zingiberaceae), *R. officinalis* and *Calendula officinalis* L. (Asteraceae) was effective and safe in patients with gingivitis, and its efficacy was comparable to that of chlorhexidine mouthwash (Mahyari et al., 2016).

*Mentha* x *piperita* (peppermint) is a hybrid of *Mentha spicata* (spearmint) and *Mentha aquatica* L. (watermint). *M.* x *piperita* is a small herb native to the Mediterranean and cultivated all over the world. It is traditionally used to treat a wide range of disorders including skin irritation, sun burns, sore throat, fever, muscle aches, nasal congestion, indigestion and infectious diseases (Camejo-Rodrigues et al., 2003; Zpetkeviciute et al., 2010; Ouelbani et al., 2016). EOs exhibited MICs ranging from 0.5–8 μg/mL in *Staphylococcus aureus, Streptococcus pneumoniae, P. aeruginosa, E. coli, Salmonella typhi* and *Klebsiella pneumoniae* (Abolfazl et al., 2014). Monoterpenes such as menthol and menthone have been reported as responsible for the antibacterial activity (Tyagi and Malik, 2011). An *in vivo* study showed that ointments from EO improved the healing process in a wound-infected model with *S. aureus* and *P. aeruginosa* (Modarresi et al., 2019). Several clinical trials have investigated the efficacy and safety of *M.* x *piperita*, but most of these focused on non-infectious disorders such as irritable bowel syndrome and non-ulcer dyspepsia (McKay and Blumberg, 2006).

*Salvia officinalis* L. (sage) is an aromatic herb native to the Mediterranean used traditionally to treat ulcers, gout, rheumatism, diarrhea, dyspepsia and inflammation in the skin and throat (Vokou et al., 1993; Ghorbani and Esmaeilizadeh, 2017). EOs exhibited MICs ranging from 12.5–225 μg/mL against *S. aureus*, *L. monocytogenes*, *E. coli* and *P. aeruginosa* (Golestani et al., 2015; Santos et al., 2017; Vaillancourt et al., 2018). Ethanol extracts exhibited MICs of 62.5 and 300 μg/mL against *Streptococcus pyogenes* and *Staphylococcus aureus*, respectively (Silva et al., 2019; Wijesundara and Rupasinghe, 2019). *Z*-linalool oxide, **9**, **10**, **12**, and **13** are the main constituents responsible for the EO antibacterial activity (Bozin et al., 2007), while **16**, **24**, **26**, ursolic acid, epigallocatechin gallate, and chlorogenic acid could be involved in the antibacterial activity of the alcoholic extract (Ghorbani and Esmaeilizadeh, 2017).

*Ocimum basilicum* (basil) is traditionally used to treat headaches, coughs, diarrhea, warts and digestive disorders (Frei et al., 1998; Loi et al., 2005; Dolatkhahi et al., 2014). EOs of the aerial parts exhibited MICs ranging from 0.6–50 μg/mL against *Salmonella typhimurium*, *Vibrio cholerae* and *Streptococcus pyogenes*, while the methanol extract of its seeds exhibited an MIC of 25 μg/mL against *Mycobacterium tuberculosis* (Gemechu et al., 2013; Ozdikmenli and Demirel Zorba, 2015; Snoussi et al., 2016; Helal et al., 2019). The main constituent in *O. basilicum* EO is **8**, it is thought to be largely responsible for its antibacterial activity, but seasonal variations of its concentration could lead to reduced antibacterial effects in summer (Hussain et al., 2008). Bioactive phenolics such as **16**, chicoric acid and caftaric acid might be responsible for the antibacterial activity in the methanol extract (Hossain et al., 2010).

#### Fabaceae

Of the 93 Fabaceae species exhibiting antibacterial activity, the ten most active were *Dichrostachys cinerea* (L.) Wight & Arm., *Albizia myriophylla* Benth., *Glycyrrhiza triphylla* Fisch. & C.A.Mey., *Copaifera reticulata* Ducke, *Acacia karroo* Hayne, *Albizia gummifera* (J.F.Gmel.) C.A.Sm., *Glycyrrhiza glabra* L., *Calpurnia aurea* (Aiton) Benth., *Copaifera paupera* (Herzog) Dwyer and *Copaifera publifora* Benth.

*Dichrostachys cinerea* is a shrub native to Africa. Its twigs are used in traditional treatments for acne vulgaris, while leaves and fruits are used for the treatment of diarrhea, fever and headache (Shandukani et al., 2018). Twigs of *D. cinerea* extracted with dichloromethane and methanol (1:1) had an MIC against *Staphylococcus epidermidis* of 0.19 μg/mL (Nciki et al., 2016); however, in the same study, no activity was found on eight other bacterial pathogens tested.

*Albizia myriophylla* is a liana which is mainly found in Southeast Asia. Its leaves, roots, flowers and wood are traditionally used in the treatment of fever, wounds, constipation, earache, digestive disorders, cough and oral diseases (Saralamp, 1996; Chotchoungchatchai et al., 2012). The wood ethanol extract had an MIC of 3.9 μg/mL against the oral pathogen *Streptococcus mutans*. Lupinifolin (**17**) was found to be responsible for this activity by damaging cell membranes, resulting in cell leakage (Limsuwan et al., 2018).

*Glycyrrhiza glabra* (licorice) is a widely used medicinal plant native to southern Europe and some parts of Asia. In traditional Chinese medicine (TCM), it is used for arthritis, bronchitis, cough, fatigue, spasms and pain (Jiang et al., 2020). It is also considered an “assistant drug” in TCM as it enhances the effectiveness of other ingredients in herbal formulations (Wang et al., 2013). In Ayurveda, it is used to relieve inflammations, eye diseases, throat infections, peptic ulcers, arthritis and liver conditions (Gupta et al., 2008; Jaiswal et al., 2016). The stem methanol extract exhibited an MIC of 10 μg/mL against *P. aeruginosa* (Chakotiya et al., 2016) while the root ethanol extract demonstrated an MIC of 62.5 μg/mL against *Streptococcus pyogenes* (Wijesundara and Rupasinghe, 2019). Its antibacterial activity is mainly attributed to flavonoids such as glabridin (**18**), glabrol, glabrene, hispaglabridin A, hispaglabridin B, 4-*O*-methylglabridin and 3-hydroxyglabrol (Pastorino et al., 2018). An *in vivo* pharmacodynamic evaluation of a liposomal dry powder for inhalation containing licorice extract showed significant reduction in bacterial counts in the lungs and spleen of *Mycobacterium tuberculosis*-infected mice (Viswanathan et al., 2019).

*Glycyrrhiza triphylla* is a plant growing in Afghanistan, Iran, Pakistan and Turkmenistan. The aerial part EO exhibit MICs ranging from 2.7–44 μg/mL for *Micrococcus luteus*, *Listeria monocytogenes*, *Pseudomonas aeruginosa* and *S. aureus* and 87 μg/mL for *Bacillus cereus* and *Salmonella typhi* (Shakeri et al., 2017). The main constituents, **9**, β-caryophyllene (**19**), β-myrcene and α-humulene, have been reported to have antibacterial activity. In contrast with *G. glabra*, *G. triphylla* is a poorly studied plant species, needing further investigation.

Trees from the genus *Copaifera* (copaiba trees) are widely distributed in the northern part of South America. Oleoresins from copaiba are known for their bactericidal, anti-helminthic, anti-inflammatory and analgesic activities (Plowden, 2004; Da Trindade et al., 2018). *C. reticulata* oleoresin exhibited MICs ranging from 6–25 μg/mL against the oral pathogens *Porphyromonas gingivalis*, *Streptococcus mitis*, *S. salivarius* and *S. sanguinis* and a range of 25–100 μg/mL for other pathogens: *L. monocytogenes*, *Staphylococcus aureus* and *Enterococcus faecalis* (Bardají et al., 2016; Fernández et al., 2018; Vieira et al., 2018). Diterpenes such as *ent*-kaurenoic acid (**20**), kolavenic acid (13*E*)-*ent*-labda-7,13-dien-15-oic acid and *ent*-polyalthic acid have been reported to be responsible for the antibacterial activity of the oleoresin (Pfeifer Barbosa et al., 2019). *C. paupera* is another tree from the “copaiba” group. Its oleoresin inhibited growth of *L. monocytogenes, Bacillus cereus* and *S. aureus* at MICs ranging from 12.5–100 μg/mL (Fernández et al., 2018). Copalic acid, *ent*-polyalthic acid, and **20** may be responsible for the activity observed *in vitro* (Tincusi et al., 2002). Likewise, *C. publifora* oleoresin showed antibacterial activity against a number of pathogens (Fernández et al., 2018).

*Acacia karroo* is a tree distributed throughout southern Africa. The gum is used to treat abscesses, oral disorders and osteomyelitis, while the bark is employed for the treatment of colds, diarrhea, dysentery, flu, hemorrhage, ringworm and stomachache and the root is used for genitourinary disorders such as gonorrhea, syphilis, urinary schistosomiasis and venereal diseases (Maroyi, 2017). A methanol extract of aerial parts showed growth inhibitory activity against *S. aureus*, *Micrococcus luteus* and *Pseudomonas aeruginosa*, with MICs ranging from 7.5–125 μg/mL. Methanol extracts of the stems exhibited MICs of 78–156 μg/mL against ampicillin-resistant *Klebsiella pneumoniae*, MRSA and beta-lactamase producing *E. coli* (Madureira et al., 2012; Nielsen et al., 2012). Activity has been attributed to β-sitosterol (**21**) and epigallocatechin (**22**) (Nyila et al., 2012).

#### Asteraceae

Of the 76 species from the Asteraceae family with antibacterial activity, the ten most active species were *Tanacetum polycephalum* Sch.Bip., *Xanthium strumarium* L., *Echinops kebericho* Mesfin, *Cota palaestina* Reut. ex Unger & Kotschy, *Mikania glomerata* Spreng., *Artemisia abyssinica* Sch.Bip. ex A.Rich., *Matricaria chamomilla* L., *Rhanterium suaveolens* Desf., *Litogyne gariepina* (DC.) Anderb. and *Achyrocline satureioides* (Lam.) DC.

*Tanacetum polycephalum* is an aromatic plant mainly distributed across Iraq, Iran and Turkey, where it is traditionally used to treat respiratory tract infections, arthritis, psoriasis, headache, migraines and diabetes (Abad et al., 1995). The aerial part EO exhibited MICs of 0.36–10 μg/mL against *Staphylococcus aureus*, *Bacillus subtilis*, *Escherichia coli* and *Salmonella typhi* (Rezazadeh et al., 2014). Compounds **10**, **11**, **13**, and isomenthol are the four main compounds likely responsible for the antibacterial activity.

*Xanthium strumarium* (cocklebur) is an herbaceous plant widely distributed across the world. It is used to treat bacterial and fungal infections, diabetes, skin pruritus, allergic rhinitis and rheumatoid arthritis (Fan et al., 2019). Leaf EO demonstrated MICs ranging from 0.5–20.5 μg/mL against *S. aureus, B. subtilis, Klebsiella pneumoniae* and *Pseudomonas aeruginosa* (Sharifi-Rad et al., 2015). Two of the EO main constituents, **9** and **13**, may be responsible for the antibacterial activity.

*Echinops kebericho* is a plant endemic to Ethiopia. The root is traditionally used to treat fever, headache, stomachache, malaria and cough (Ameya et al., 2016). Ethanol and methanol extracts of the root exhibited MICs of 3–25 μg/mL against *S. aureus*, *Enterococcus faecalis* and *E. coli*. A major constituent, dehydrocostus lactone, is a sesquiterpene lactone that has demonstrated antibacterial activity (Lee et al., 2014).

*Cota palaestina* is an herbaceous plant distributed in different regions of Jordan used traditionally as a diuretic and antiedemic (Güzel et al., 2015). The EO of the flowers showed MICs of 6–25 μg/mL against *Staphylococcus epidermidis*, *S. aureus* and *B. subtilis* and 53 and 75 μg/mL for *E. coli* and *P. aeruginosa*, respectively (Bardaweel et al., 2014). Sesquiterpenes such as spathulenol, germacrene D (**23**) and caryophyllene oxide may be involved in the antibacterial activity.

*Mikania glomerata* is a vine native to Southeastern Brazil and cultivated in several South American countries. The leaves are used to treat snake bites, fevers, stomach discomfort, rheumatism and respiratory problems (Napimoga and Yatsuda, 2010). The methanol extract of the plant exhibited MICs against *Cutibacterium acnes* (6.25 μg/mL) and *E. faecalis* (25 μg/mL) as well as against oral pathogens at a range of 12.5–18 μg/mL: *Actinomyces naeslundii*, *Porphyromonas gingivalis* and *Prevotella nigrescens* (Moreti et al., 2017). Its major constituent **20**, a diterpene, has been reported to be responsible for the antibacterial activity (Moreira et al., 2016). A clinical trial evaluating the efficacy of a mixture of *M. glomerata* and *Mikania laevigata* Sch.Bip. ex Baker in the disinfection of toothbrushes used by preschool children showed a reduction of *Streptococcus mutans* similar to that with chlorhexidine (Lessa et al., 2012).

*Artemisia* is well known due to the discovery of new antimalarial agents (artemisinin and its derivatives) in the 1970s in the species *Artemisia annua* (Tu, 2016). Since then, *Artemisia* species have been widely investigated as a source of antimicrobial compounds. Five different *Artemisia* species have been reported to exhibit antibacterial activity including *A. abyssinica*, *A. indica* Willd., *A. vestita* Wall. ex Besser, *A. herba-alba* Asso and *A. fragrans* Willd. The two most active species are discussed here. *A. abyssinica* is an herbaceous plant distributed throughout Africa and the Middle East. It is used to treat cough and respiratory disorders as well as constipation and rheumatism (Bekalo et al., 2009). A leaf methanol extract exhibited MICs of 6.25 and 12.5 μg/mL against *Mycobacterium tuberculosis* and *M. bovis*, respectively (Gemechu et al., 2013). An EO of *A. indica*, a species native to Asia, exhibited MICs of 32–128 μg/mL against Gram-negative bacteria such as *K. pneumoniae*, *P. aeruginosa*, *Salmonella typhi* and *Shigella dysenteriae* and activity was attributed in part to a major constituent, artemisia ketone (Rashid et al., 2013). More generally, the presence of **10**, **11**, and artemisia ketone in *Artemisia* species might explain their antibacterial activity (Donato et al., 2015).

*Matricaria chamomilla* (chamomile) is an herb native to southern and eastern Europe. Traditionally, it is used to treat coughs, menstrual and gastrointestinal pains, rheumatism, eczema, skin irritations, gingivitis, and eye inflammations (Vitalini et al., 2015). Its EO showed MICs of 10–156 μg/mL against *E. coli*, *K. pneumoniae*, *Proteus mirabilis*, *P. vulgaris*, MRSA and *B. subtilis* (Cvetanović et al., 2019). α-bisabolol could be involved in the antibacterial activity observed (Romero et al., 2012). A topical application of Ad-Muc^®^, a *M. chamomilla* formulation, demonstrated faster wound healing than corticosteroids on tongue ulcers in a mouse model (Martins et al., 2009). In clinical studies, a wild chamomile mouthwash infusion administered to a patient with methotrexate-induced oral mucositis successfully treated the patient within 4 weeks (Mazokopakis et al., 2005), and the efficacy of *M. chamomilla* on oral mucositis was confirmed in a clinical trial evaluating 98 head and neck cancers treated with a chamomile oral rinse (Petronilho et al., 2012).

#### Myrtaceae

Of the 37 species belonging to the Myrtaceae family with reported antibacterial activity, the ten most active were *Rhodomyrtus tomentosa* (Aiton) Hassk., *Eucalyptus camaldulensis* Dehnh., *Syncarpia glomulifera* (Sm.) Nied., *Corymbia torelliana* (F.Muell.) K.D.Hill & L.A.S.Johnson, *Myrtus communis* L., *Syzygium cordatum* Hochst. ex Krauss, *Melaleuca armillaris* (Sol. ex Gaertn.) Sm., *Eugenia brevistyla* D.Legrand, *Eugenia catharinae* O. Berg., *Eucalyptus globulus* Labill. and *Psidium guajava* L. Half of these plant species are native to Australia (i.e., *E. camaldulensis*, *S. glomulifera*, *C. torelliana*, *M. armillaris* and *E. globulus*). Here, we focus on the five most represented species by number of publications: *Psidium guajava*, *Rhodomyrtus tomentosa*, *Eucalytpus camaldulensis*, *Eucalytpus globulus* and *Syzygium aromaticum* (L.) Merr. & L.M.Perry.

*Psidium guajava* (guava) is a pantropical tree native to Central and South America. One of the main traditional uses of *P. guajava* is as an antidiarrheal (Hirudkar et al., 2020). Other medicinal uses include the treatment of bacterial infections, coughs, diabetes, dysentery, fevers, leucorrhea, rheumatism, toothaches and wounds (Gutiérrez et al., 2008). Methanol extracts of leaves showed MICs of 31–128 μg/mL against *Staphylococcus aureus*, *Pseudomonas aeruginosa* and *Enterobacter aerogenes*. Acetone extracts of the leaves exhibited MICs of 78 μg/mL on *Salmonella typhi*, *Enterococcus faecalis* and *Shigella flexneri* (Bai et al., 2015; Bisi-Johnson et al., 2017; Dzotam and Kuete, 2017). A mixture of flavonoids (including quercetin (**24**), quercetin derivatives, kaempferol, avicularin, guaijaverin and morin glycosides) is likely to be responsible for the antibacterial activity (Arima and Danno, 2002; Sanches et al., 2005). Various *in vivo* models were used to evaluate the effect of guava on infectious diarrhea. This includes *Citrobacter rodentium*-infected mice, which showed quicker clearance of infection after 19 days with an hydroalcoholic extract of *P. guajava* given at 300 mg/kg/day (Gupta and Birdi, 2015), and *V. cholerae*-infected mice which demonstrated intestinal ameliorative effects after 4 h infection when treated with an ethanol extract of *P. guajava* leaves at 250 mg/kg (Shittu et al., 2016). A randomized double-blind clinical trial was performed to evaluate the efficacy of the phytodrug QG5^®^, containing *P. guajava* leaves with a standardized concentration of flavonoids, on patients with infectious gastroenteritis. QG5^®^ significantly reduced the duration of abdominal pain in these patients with no serious adverse effects (Lozoya et al., 2002).

*Rhodomyrtus tomentosa* is an evergreen shrub native to Southeast Asia. The berries, leaves and stems of *R. tomentosa* are traditionally used to treat diarrhea, wound infections and traumatic hemorrhage (Ong and Nordiana, 1999; Li and Xing, 2016). The leaf ethanol extract exhibited MICs of 7.8–32 μg/mL against Gram-positive bacteria such as *Bacillus cereus, S. aureus* (including MRSA), *Streptococcus mutans*, *S. agalactiae* and *Listeria monocytogenes* (Phoem and Voravuthikunchai, 2012; Limsuwan and Voravuthikunchai, 2013; Odedina et al., 2015; Na-Phatthalung et al., 2017; Zhao et al., 2019). Rhodomyrtone (**25**), an acylphloroglucinol, is responsible for the antibacterial (bactericidal) activity against Gram-positive bacteria but is not active against Gram-negative bacteria (Limsuwan et al., 2011). Tomentosone C, another acylphloroglucinol, could also be involved in the antibacterial activity (Liu et al., 2016). The ethanol extract has demonstrated activity in *Staphylococcus aureus* infections in *in vivo* models of bovine mastitis, and it also reduced the mortality rate of *Streptococcus agalactiae*-infected Nile tilapia (Na-Phatthalung et al., 2017; Mordmuang et al., 2019).

Trees from the *Eucalyptus* genus are native to Australia and are widely distributed throughout the world. The red gum from *E. camaldulensis* was directly applied to abrasions and cuts by Australian Aboriginal peoples (Locher and Currie, 2010; Knezevic et al., 2016), and in Africa, the gum is used to treat diarrhea and sore throat, while the leaves are used to treat respiratory problems (Knezevic et al., 2016). A stem bark hexane extract and leaf extract exhibited an MIC of 4 and 6.25 μg/mL against *Mycobacterium tuberculosis*, respectively (Lawal et al., 2012). The leaf EO exhibited MICs at 5 μL/mL against *Escherichia coli*, *Shigella* spp. and *Bacillus* spp. (Nasir et al., 2015). Quercitrin, naringenin, kaempferol, **24**, and ellagic acid (**26**) might play a role in the antibacterial activity for non-EO extracts, while **1**, **2**, **3**, **5**, **6**, **7**, **11**, **12** are likely to play a role in leaf hydrodistillates (Aleksic Sabo and Knezevic, 2019). In a study of 100 individuals, toothpaste with *E. camaldulensis* EO (0.8 mg/mL) exhibited a significant reduction of dental biofilm after 4 weeks over that of the positive control formulation containing 0.2% chlorhexidine (2 mg/mL) (Rasooli et al., 2009).

*Eucalyptus globulus* leaves are widely used to treat respiratory problems (Andrade-Cetto, 2009). A leaf ethanol extract exhibited MICs of 125 and 250 μg/mL against *Mycobacterium smegmatis* and *M. ulcerans*, respectively (Tsouh Fokou et al., 2016). Its EO inhibited growth of *E. coli*, *Streptococcus iniae* and *Staphylococcus aureus* (Roomiani et al., 2013; Golestani et al., 2015; Thielmann et al., 2019). The main constituents of *E. globulus* EO, **11** and **12**, are likely to be responsible for the EO activity (Luís et al., 2016), while phenolic compounds might be involved in the activity of ethanol extracts (Boulekbache-Makhlouf et al., 2013). *Eucalyptus* oil has been approved for use by the US Food and Drug Administration and many over-the-counter (OTC) products include it in their formulations (Ghasemian et al., 2019). It is also recommended as a steam inhalation for treating chronic sinusitis (Ivker, 2018). The European Medicines Agency noted a paucity in clinical data on its efficacy, but the presence of **11** supports action on upper respiratory diseases. However, precautions should be taken as side effects can occur (e.g., allergic reactions, nausea and vomiting) and the inhalation and cutaneous use of eucalyptus EOs among young children (<30 months) can lead to laryngospasm (EMA, 2013).

*Syzygium aromaticum* (clove) is a tree native to Indonesia. The EO of its buds is widely used in dental care as an antiseptic and analgesic (Rosas-Piñón et al., 2012). Other traditional uses include the treatment of cuts and wounds. The bud EO exhibited MICs of 50, 100 and 400 μg/mL against *Haemophilus ducreyi*, *S. aureus* and *E. coli*, respectively. Leaf EO demonstrated MICs of 250–500 μg/mL against dental pathogens such as *Fusobacterium nucleatum, Porphyromonas gingivalis* and *Streptococcus mitis* (Bersan et al., 2014; Lindeman et al., 2014; Thielmann et al., 2019). Eugenol (**27**), a phenolic compound, is the main EO constituent with a concentration ranging from 47.64 to 88.58% (Chaieb et al., 2007). It has demonstrated antibacterial activity through membrane cell lysis and leakage of proteins and lipid contents (Kamatou et al., 2012). Eugenyl acetate, benzyl alcohol and **19** are other compounds present in the plant with antibacterial activity.

#### Anacardiaceae

A total of 33 species of Anacardiaceae were investigated for their antibacterial properties. Four percent of the species present in Anacardiaceae were investigated, the highest percentage of any family. The five species with the lowest MICs were *Anacardium occidentale* L. (Figure 14C), *Schinus terebinthifolia* Raddi, *Pistacia terebinthus* L., *Mangifera indica* L. and *Pistacia lentiscus* L.

*Anacardium occidentale* (cashew) is a large tropical evergreen tree native to Central America, South America and the Caribbean Islands, but it has now spread throughout Southeast Asia and Africa. The cashew apple is a fleshy fruit, the pulp of which can be processed into drinks or distilled into liquor. In India, cashew fruits are used to treat asthma and headache, while cashew seeds are used for burns and male fertility (Morvin Yabesh et al., 2014). The leaves and bark of *A. occidentale* are used for malaria in Nigeria (Dike et al., 2012) and for diarrhea and fever in Peru (Odonne et al., 2013). The aqueous bark extract exhibited MICs of 3–6 μg/mL against oral pathogens such as *Streptococcus mitis*, *S. mutans* and *S. salivarius* (de Araújo et al., 2018). The leaf methanol extract exhibited MICs of 7.5–15 μg/mL against *Enterococcus faecalis*, *Staphylococcus aureus* and *Micrococcus luteus* (Madureira et al., 2012). Phenolic compounds such as anacardic acids, cardols and quercetin glycosides are suspected to be responsible for the activity (Tan and Chan, 2014; Ajileye et al., 2015). In a randomized clinical trial, a mouthwash containing stem bark EO diluted in ethanol (10%) showed similar results on the reduction of plaque formation and gingival bleeding after 30 days as a mouthwash with chlorhexidine (0.12%) (Gomes et al., 2016).

*Schinus terebinthifolia* (Brazilian peppertree) is a spreading shrub or small tree native to subtropical and tropical South America; it is an invasive weed in Florida (Williams et al., 2005). It is used to treat inflammatory, dermatological and hemostatic diseases (Salem et al., 2018). An acetone extract of the fruits exhibited MICs ranging from 8–16 μg/mL for *Acinetobacter baumannii*, *S. aureus* and *Escherichia coli*. The fruit EO exhibited an MIC of 32 and 128 μg/mL against *Pseudomonas aeruginosa* and *Micrococcus flavus,* respectively (Salem et al., 2018). A fruit extract significantly reduced dermonecrosis following skin challenge in mice infected with a virulent MRSA strain, though this activity was credited to anti-virulence rather than growth inhibitory effects (Muhs et al., 2017), with triterpenoid acids being responsible for the activity (Tang et al., 2020).

*Pistacia terebinthus* is a tree widely distributed in the Middle East and the Mediterranean region. It is used to treat asthma, bronchitis, burns, cold, leprosy and urinary afflictions (Bozorgi et al., 2013). The fruit, galls and leaves extracted as EOs exhibited MICs against MRSA strains at 0.32, 0.64 and 1.28 μL/mL, respectively. The main compounds in these EOs are **9**, **12**, and β-pinene (Pulaj et al., 2016).

*Pistacia lentiscus* (mastic) is an evergreen shrub or small tree that grows up to 4 m tall and is native throughout the Mediterranean region and the Canary Islands; it is cultivated elsewhere for its aromatic resin. It is used to treat stomachache and urinary afflictions in Turkey (Cakilcioglu and Turkoglu, 2010) and as an antiprostatitic, antiseptic and hypotensive in the Iberian Peninsula (Agelet and Vallès, 2003). Mastic gum is a natural resin derived from the stem and leaves, and it is traditionally used to treat gastrointestinal ailments (Ali-Shtayeh et al., 2000). The EO of the aerial parts was active against MRSA (MIC = 120 μg/mL) (Lahmar et al., 2017); **9**, **12**, myrcene, and β-pinene might be involved in the activity (Hayder et al., 2005). An *in vivo* study showed that mastic gum from *P. lentiscus* var. *chia* reduced *Helicobacter pylori* colonization in mice over a period of 3 months and that triterpenoid acids may be responsible for the activity (Paraschos et al., 2007).

*Mangifera indica* (mango) is a tree native to tropical Asia that now grows in most tropical countries. Mango bark is used for the treatment of cough, diarrhea, gastric disorders, syphilis and urinary tract disorders. The leaves are used to treat bronchitis, colds, diarrhea, fever, malaria and wounds (Ediriweera et al., 2017). A bark methanol extract was active against *P. aeruginosa* (MIC = 32 μg/mL), while a methanol leaf extract exhibited an MIC of 125 μg/mL against *P. aeruginosa* and 250 μg/mL against *S. aureus*; a leaf hexane extract had an MIC of 250 μg/mL against *Mycobacterium smegmatis* (Bai et al., 2015; Tsouh Fokou et al., 2016; Dzotam and Kuete, 2017). Mangiferin, a xanthone, as well as gallotanins, kaempferol and **24**, might be responsible for the antibacterial activity (Engels et al., 2011; Ediriweera et al., 2017). A mango leaf extract carbopol hydrogel formulation showed *in vitro* and *ex vivo* antibacterial activity against *S. aureus* (Chirayath et al., 2019).

#### Rubiaceae

Rubiaceae is well-known as the family of *Cinchona* spp., the source of quinine and other anti-malarial alkaloids (Gurung and De, 2017). A total of 33 species from the Rubiaceae family were studied for antibacterial activity, mainly as antimycobacterials: 54.4% MIC values reported for extracts from Rubiaceae were against *Mycobacterium* spp. The species with the best activity from this family were *Pavetta lanceolata* Eckl. and *Cephalanthus natalensis* Oliv. *Sarcocephalus latifolius* (Sm.) E.A.Bruce exhibited the best activity against non-mycobacterial species.

*Pavetta lanceolata* (weeping bride’s bush) is a tree or shrub native to southern Africa. Its leaves are traditionally used as an anti-emetic and the root as a tonic (Arnold and Gulumian, 1984). A leaf acetone extract inhibited growth of *Mycobacterium smegmatis*, *M. aurum* and *M. tuberculosis* with MICs ranging from 12–120 μg/mL (Aro et al., 2016). However, *in vitro* cytotoxicity tests with human liver and kidney cells yielded LD_50_ values of 277 μg/mL and 97 μg/mL, respectively, indicating that toxicity may be a barrier to developing antimycobacterials from *P. lanceolata* (Aro et al., 2015).

*Cephalanthus natalensis* (bush berry) is a shrub native to southern Africa. The fruit is eaten and the bark is used to treat bleeding (Dlamini and Solomon, 2019). The acetone extract of *C. natalensis* leaves exhibited MICs ranging from 17–170 μg/mL against *Micrococcus flavus*, *Mycobacterium tuberculosis*, *M. aurum* and *M. smegmatis* (Aro et al., 2015; Aro et al., 2016). Similar to *P. lanceolata*, the *C. natalensis* extract exhibits cytotoxicity in human liver and kidney cells with LD_50_ values of 138 μg/mL and 76 μg/mL, respectively (Aro et al., 2015). However, a combination of *C. natalensis* extracts interacted synergistically with the established antimycobacterial rifampicin, achieving a *M. tuberculosis* MIC with 2.5 μg/mL extract and 2.25 μg/mL rifampicin (Fractional Inhibitory Concentration Index, FICI = 0.31), suggesting that exploiting drug interactions may be a pathway to avoid toxicity (Aro et al., 2016).

*Sarcocephalus latifolius* is native to West Africa and is traditionally used to treat diarrhea, dysentery and fever (including malaria); it is also used as a chewing stick for oral conditions such as dental caries (Tekwu et al., 2012). A methanol extract of the stem bark was found to have good activity against Gram-negative bacteria, inhibiting growth of *Escherichia coli*, *Salmonella typhi* and *Shigella flexneri* (MIC: 32 μg/mL); the extract was also active against *Staphylococcus aureus* and *Bacillus cereus* MICs ranging 64–128 μg/mL (Tekwu et al., 2012). A separate study of *S. latifolius* stem and bark methanol extract identified six bioactive alkaloids: latifoliamides A-E and angustoline (Agomuoh et al., 2013).

#### Apiaceae

The Apiaceae family is known to contain aromatic herbs and many poisonous species. Of the 31 species from the Apiaceae family studied as antibacterials, *Trachyspermum ammi* (L.) Sprague, *Coriandrum sativum* L. (Figure 14D) and *Eryngium* spp. exhibited the most activity.

*Trachyspermum ammi* (ajowan) is an herb native to Iran and India (Moosavi-Nasab et al., 2016). Its fruit is used as a spice and to preserve meat (Mahmoudzadeh et al., 2016); an aqueous extract of *T. ammi* is also used to treat flu in children (Moosavi-Nasab et al., 2016). The fruit EO was found to inhibit growth of *Bacillus cereus*, *Listeria monocytogenes*, *Staphylococcus aureus*, *Enterobacter aerogenes*, *Pseudomonas putida* and *Escherichia coli* with an MIC of 0.4 μL/mL. The major constituents of this EO were **3**, **5**, and **6** (Moosavi-Nasab et al., 2016).

*Coriandrum sativum* (coriander) is an annual herb which grows wild mostly in western Asia and southern Europe. *C. sativum* seeds and leaves are well known for their utility as a seasoning in cooking. In traditional medicine, coriander is used as a stimulant, stomachic, carminative, antispasmodic, diuretic and anthelminthic (Sivasankari et al., 2014) and to treat asthma, cough and bronchitis (Kayani et al., 2014). *C. sativum* EO showed MICs ranging from 0.4–62 μg/mL against *E. coli*, *Proteus mirabilis*, *Fusobacterium nucleatum* and *Staphylococcus aureus*, *S. hyicus* and *Streptococcus mitis* (Bersan et al., 2014; Bogavac et al., 2015; Vaillancourt et al., 2018). The constituents of *C. sativum* EO are primarily monoterpenes, with **8**, **9**, and linalyl acetate being the most prominent (Bogavac et al., 2015). The proposed antibacterial mechanism of action (MOA) of *C. sativum* EO is disruption of the cell membrane (Silva et al., 2011). In its wide use as an herb and spice, *C. sativum* has not exhibited significant toxicity (Bogavac et al., 2015).

*Eryngium* is the largest genus in Apiaceae and is found on every continent except Antarctica. Several *Eryngium* species are used in various traditional medicine practices to treat conditions including hypertension, gastrointestinal distress, diarrhea, burns and fevers (Landoulsi et al., 2016). Five Mediterranean *Eryngium* species exhibited antibacterial activity including *E. amethystinum* L., *E. campestre* L., *E. glomeratum* Lam., *E. palmatum* Pančić & Vis. and *E. pusillum* L. with MICs of 2–500 μg/mL (Landoulsi et al., 2016; Matejić et al., 2018). The EO of *E. amethystinum* aerial parts exhibited MICs of 2 μg/mL against *Staphylococcus aureus* and contained **22**, α-gurjunene and γ-muurolene. The acetone extract of *E. campestre* showed MICs of 4–10 μg/mL against *Proteus mirabilis*, *Escherichia coli* and *S. aureus* (Matejić et al., 2018). A 2015 review of the phytochemistry and antimicrobial activity of *Eryngium* called for toxicity and MOA experiments for the further development of *Eryngium* extracts as antibacterials (Erdem et al., 2015).

### Conclusion and Future Directions

We have reported the antibacterial activities of 958 plants by reviewing the literature published from 2012 to 2019, which represents 66% of the total literature on this subject since 1946. Our review was focused to include the literature that followed established guidelines for botanical authentication and biological screening. These numbers of plants and plant natural products, while large, are miniscule in comparison to the 374,000 (Christenhusz and Byng, 2016) estimated total plants, or even the 28,187 medicinal species used by humans (MNPS, 2020). Medicinal plants and their natural products thus remain largely untapped as sources of antibacterial compounds.

We are highly encouraged by the number of highly bioactive extracts identified in this review. Eloff defined an extract or fraction as having significant antibacterial activity if the MIC against the given organism is equal to or less than 100 μg/mL (Eloff, 2004), and Kuete defined a compound as having significant antibacterial activity if the MIC is equal to or less than 10 μg/mL (Kuete, 2010). To this extent, we report 358 plant extracts that fall under Eloff’s cutoff for at least one bacterial species. Gibbons defined essential oils as having significant activity if the MIC is equal to or less than 5 μL/mL (Gibbons, 2004). Since the density of essential oils is inferior but close to 1 g/mL, we also considered essential oils with MICs reported in µg/mL as significant if the MIC is equal to or less than 5 μg/mL. To this extent, we report 50 essential oils as having high antibacterial activity against at least one bacterial species. Such observations confirm that plants and their natural products represent promising sources of antibacterials and that their continued exploration represents a productive trajectory.

There are a number of research errors that we commonly encountered in the literature which precluded paper inclusion into our data analysis. Regarding botanical authentication, many publications reported collecting plant material from markets without proper identification, or they did not mention depositing a corresponding herbarium voucher specimen. As stated by previous authors, rigorous assessment of the taxonomic nomenclature of plants should be addressed at an early stage in ethnobotanical surveys (Rivera et al., 2014). From a chemical perspective, researchers should be aware of the information gained from and resolution limitations of thin layer chromatography methods, which can lead to oversimplified conclusions regarding chemistry. Also, the lack of standard methods to evaluate the antibacterial activity of plants is a major concern. In particular, the agar diffusion assay (also known as the disc diffusion method) is not appropriate for the quantitative analysis of plant extracts as non-polar compounds can fail to diffuse and thus lead to false results (Tan and Lim, 2015). Instead, broth microdilution or agar dilution assays should be used for quantifying the antibacterial activity of plant extracts. The CLSI (CLSI, 2012) and other protocols (Eloff, 1998; Andrews, 2001; Sarker et al., 2007; Wiegand et al., 2008) contain precise information on the materials and equipment needed as well as the inoculum density, time of incubation and positive controls to be used. Furthermore, we propose specific cutoffs for therapeutically relevant MIC values be implemented in future work (Gibbons, 2004; Cos et al., 2006; Aro et al., 2019). Researchers should also take into consideration the possible toxicity of drugs, employing systematic counterscreening against human cell lines whenever possible (Cos et al., 2006).

Beyond expanding and refining bioprospecting and the *in vitro* studies addressed above, ethnobotanical antibiotic drug discovery endeavors would benefit greatly from further preclinical studies. Such studies include compound isolation from bioactive extracts, mechanism of action studies, *in vivo* testing in animal models of infection, structural modification of compounds to improve pharmacodynamics and pharmacokinetics, structure-activity relationship (SAR) analyses, and incorporating emerging trends into the traditional workflow. Also, synergistic interactions within plant extracts and between plant compounds and antibiotics should be further studied to unveil the mechanism beyond the antibacterial activity of these compounds and then discover multiple pathways to be targeted. As an example, Berberis species producing the antibacterial alkaloid berberine are also able to synthesize a compound (5′-methoxyhydnocarpin) responsible for the inactivation of efflux pumps in *P. aeruginosa*, thus potentiating the antibacterial effect of berberine (Stermitz et al., 2000). Many research groups may build some of these capabilities themselves or collaborate with other groups to share expertise. In particular, international collaborations between resource-rich institutions with scientific partners in biodiversity rich areas of the world can lead to mutual benefits in equitable access and benefit sharing for research teams, government bodies and community partners. Such collaborations allow for biodiversity to be probed more deeply than otherwise possible and result in expertise sharing, research training opportunities for students and faculty and joint publications and patents under the principles of the United Nations Convention on Biological Diversity and the Nagoya Protocol (UN, 2011). Addressing the threat of antibiotic resistance will take a variety of strategies working together, and the ethnobotanical approach offers the tools to unlock and apply the useful chemistry of plants to antibiotic compound discovery.

## Author Contributions

FC and TS have equal contribution as co-first author, CLQ, FC and GP designed the study, FC led the literature search, FC, TS, GP, JTL, MD, LM, AMS, SS, and DRF performed the literature search, TS performed the phylogenetic assessment. All authors completed the data analysis, wrote the manuscript, and approved the final version of the manuscript.

## Funding

This work was supported by the National Institute of Allergy and Infectious Disease (R21 AI136563 to CLQ), Emory University development funds to CLQ, and a graduate student fellowship from The Jones Center at Ichuaway to LM.

## Conflict of Interest

The authors declare that the research was conducted in the absence of any commercial or financial relationships that could be construed as a potential conflict of interest.
