# Supplementary material for: A Systematic Review of Plants With Antibacterial Activities: A Taxonomic and Phylogenetic Perspective
Source: Front Pharmacol. 2021 Jan 8;11:586548. doi: 10.3389/fphar.2020.586548 (PMC7821031; doi:10.3389/fphar.2020.586548)
Supplement: Supplementary file 1 [file table1.docx]

Supplementary Material S2

**A Systematic Review of Plants with Antibacterial Activities: A Taxonomic and Phylogenetic Perspective**

François Chassagne,^1, %^ Tharanga Samarakoon,^2 %^ Gina Porras,^1^ James T. Lyles,^1^ Micah Dettweiler,^3^ Lewis Marquez,^+^ Akram M. Salam,^4^ Sarah Shabih,^1^ Darya Raschid Farrokhi,^1^ and Cassandra L. Quave**^*^**^,1,2,3,4^

^1^Center for the Study of Human Health, Emory University, 1557 Dickey Dr., Atlanta, Georgia 30322

^2^Emory University Herbarium, Emory University, 1462 Clifton Rd NE, Room 102, Atlanta, Georgia 30322

^3^Department of Dermatology, Emory University, 615 Michael St., Whitehead 105L, Atlanta, Georgia 30322

^4^Molecular and Systems Pharmacology Program, Laney Graduate School, Emory University, 615 Michael St., Whitehead 115, Atlanta, Georgia 30322

^%^Equal Contributions, Co-first authors

**^*^Correspondence:**

Cassandra L. Quave

[cassandra.leah.quave@emory.edu](mailto:cassandra.leah.quave@emory.edu)

**Supplementary Material S2.1**  Percent of species and genera studied for antibacterial properties by family. Total number of genera and species for each family are reported according to the Angiosperm working group version 14 (Stevens, 2001 Onwards).

| Order | Family | Nº Genera Studied | Total Nº Genera | % Genera Studied | Nº Species Studied | Total Nº Species | % Species Studied |
| --- | --- | --- | --- | --- | --- | --- | --- |
| Alismatales | Araceae | 3 | 144 | 2 | 3 | 3645 | 0 |
| Apiales | Apiaceae | 23 | 434 | 5 | 31 | 3780 | 1 |
| Apiales | Araliaceae | 2 | 43 | 5 | 2 | 1450 | 0 |
| Apiales | Pittosporaceae | 1 | 6 | 17 | 2 | 200 | 1 |
| Arecales | Arecaceae | 3 | 188 | 2 | 3 | 2585 | 0 |
| Asparagales | Amaryllidaceae | 3 | 73 | 4 | 7 | 1605 | 0 |
| Asparagales | Asparagaceae | 4 | 153 | 3 | 5 | 2595 | 0 |
| Asparagales | Asphodelaceae | 2 | 41 | 5 | 9 | 900 | 1 |
| Asparagales | Hypoxidaceae | 3 | 7 | 43 | 4 | 100 | 4 |
| Asparagales | Iridaceae | 2 | 66 | 3 | 2 | 2120 | 0 |
| Asparagales | Orchidaceae | 1 | 880 | 0 | 1 | 26000 | 0 |
| Asparagales | Xanthorrhoeaceae | 1 | 1 | 100 | 1 | 30 | 3 |
| Asterales | Asteraceae | 58 | 1620 | 4 | 76 | 25040 | 0 |
| Austrobaileyales | Schisandraceae | 1 | 3 | 33 | 1 | 92 | 1 |
| Boraginales | Boraginaceae | 2 | 94 | 2 | 2 | 1793 | 0 |
| Brassicales | Brassicaceae | 2 | 341 | 1 | 2 | 3973 | 0 |
| Brassicales | Capparaceae | 2 | 16 | 13 | 3 | 480 | 1 |
| Brassicales | Caricaceae | 1 | 6 | 17 | 1 | 34 | 3 |
| Brassicales | Cleomaceae | 1 | 25 | 4 | 2 | 270 | 1 |
| Brassicales | Moringaceae | 1 | 1 | 100 | 1 | 12 | 8 |
| Brassicales | Salvadoraceae | 1 | 3 | 33 | 2 | 11 | 18 |
| Brassicales | Tropaeolaceae | 1 | 1 | 100 | 1 | 105 | 1 |
| Canellales | Canellaceae | 1 | 5 | 20 | 1 | 13 | 8 |
| Canellales | Winteraceae | 1 | 5 | 20 | 1 | 105 | 1 |
| Caryophyllales | Aizoaceae | 1 | 124 | 1 | 1 | 1180 | 0 |
| Caryophyllales | Amaranthaceae | 3 | 180 | 2 | 4 | 2050 | 0 |
| Caryophyllales | Caryophyllaceae | 1 | 101 | 1 | 1 | 2200 | 0 |
| Caryophyllales | Droseraceae | 1 | 3 | 33 | 1 | 205 | 0 |
| Caryophyllales | Plumbaginaceae | 2 | 29 | 7 | 3 | 836 | 0 |
| Caryophyllales | Polygonaceae | 4 | 55 | 7 | 8 | 1110 | 1 |
| Caryophyllales | Talinaceae | 1 | 3 | 33 | 1 | 27 | 4 |
| Caryophyllales | Tamaricaceae | 1 | 3 | 33 | 1 | 90 | 1 |
| Celastrales | Celastraceae | 7 | 94 | 7 | 7 | 1410 | 0 |
| Cornales | Curtisiaceae | 1 | 1 | 100 | 1 | 1 | 100 |
| Cucurbitales | Cucurbitaceae | 6 | 98 | 6 | 8 | 1000 | 1 |
| Cupressales | Cupressaceae | 4 | 30 | 13 | 5 | 133 | 4 |
| Cupressales | Taxaceae | 1 | 6 | 17 | 1 | 30 | 3 |
| Dilleniales | Dilleniaceae | 3 | 11 | 27 | 3 | 300 | 1 |
| Dioscoreales | Dioscoreaceae | 1 | 4 | 25 | 2 | 870 | 0 |
| Dipsacales | Adoxaceae | 1 | 4 | 25 | 2 | 23 | 9 |
| Dipsacales | Caprifoliaceae | 3 | 31 | 10 | 3 | 890 | 0 |
| Ericales | Ebenaceae | 2 | 4 | 50 | 7 | 855 | 1 |
| Ericales | Ericaceae | 4 | 126 | 3 | 11 | 4215 | 0 |
| Ericales | Lecythidaceae | 1 | 25 | 4 | 1 | 353 | 0 |
| Ericales | Primulaceae | 3 | 58 | 5 | 4 | 2590 | 0 |
| Ericales | Sapotaceae | 3 | 53 | 6 | 3 | 1100 | 0 |
| Fabales | Fabaceae | 43 | 766 | 6 | 93 | 19580 | 0 |
| Fagales | Betulaceae | 1 | 6 | 17 | 1 | 145 | 1 |
| Fagales | Fagaceae | 1 | 7 | 14 | 3 | 730 | 0 |
| Fagales | Juglandaceae | 1 | 9 | 11 | 1 | 51 | 2 |
| Fagales | Myricaceae | 2 | 4 | 50 | 2 | 57 | 4 |
| Gentianales | Apocynaceae | 11 | 400 | 3 | 13 | 4555 | 0 |
| Gentianales | Gentianaceae | 1 | 102 | 1 | 1 | 1750 | 0 |
| Gentianales | Loganiaceae | 1 | 13 | 8 | 1 | 420 | 0 |
| Gentianales | Rubiaceae | 24 | 614 | 4 | 33 | 13465 | 0 |
| Geraniales | Geraniaceae | 1 | 7 | 14 | 3 | 866 | 0 |
| Geraniales | Melianthaceae | 1 | 2 | 50 | 1 | 8 | 13 |
| Gnetales | Ephedraceae | 1 | 1 | 100 | 2 | 54 | 4 |
| Gunnerales | Gunneraceae | 1 | 1 | 100 | 1 | 60 | 2 |
| Icacinales | Icacinaceae | 2 | 23 | 9 | 2 | 200 | 1 |
| Lamiales | Acanthaceae | 5 | 220 | 2 | 6 | 4000 | 0 |
| Lamiales | Bignoniaceae | 6 | 110 | 5 | 6 | 790 | 1 |
| Lamiales | Lamiaceae | 40 | 236 | 17 | 108 | 7280 | 1 |
| Lamiales | Oleaceae | 4 | 24 | 17 | 4 | 615 | 1 |
| Lamiales | Paulowniaceae | 1 | 1 | 100 | 1 | 6 | 17 |
| Lamiales | Pedaliaceae | 2 | 15 | 13 | 3 | 70 | 4 |
| Lamiales | Plantaginaceae | 1 | 90 | 1 | 1 | 1900 | 0 |
| Lamiales | Scrophulariaceae | 3 | 59 | 5 | 11 | 1880 | 1 |
| Lamiales | Verbenaceae | 7 | 31 | 23 | 13 | 918 | 1 |
| Laurales | Hernandiaceae | 1 | 5 | 20 | 1 | 55 | 2 |
| Laurales | Lauraceae | 8 | 50 | 16 | 26 | 2500 | 1 |
| Laurales | Monimiaceae | 1 | 22 | 5 | 1 | 200 | 1 |
| Liliales | Liliaceae | 1 | 15 | 7 | 1 | 610 | 0 |
| Magnoliales | Annonaceae | 11 | 110 | 10 | 15 | 2430 | 1 |
| Magnoliales | Magnoliaceae | 2 | 2 | 100 | 2 | 227 | 1 |
| Magnoliales | Myristicaceae | 1 | 20 | 5 | 1 | 475 | 0 |
| Malpighiales | Calophyllaceae | 1 | 13 | 8 | 1 | 460 | 0 |
| Malpighiales | Caryocaraceae | 1 | 2 | 50 | 1 | 27 | 4 |
| Malpighiales | Chrysobalanaceae | 2 | 18 | 11 | 2 | 530 | 0 |
| Malpighiales | Clusiaceae | 3 | 14 | 21 | 8 | 800 | 1 |
| Malpighiales | Euphorbiaceae | 12 | 218 | 6 | 28 | 6745 | 0 |
| Malpighiales | Hypericaceae | 3 | 9 | 33 | 8 | 477 | 2 |
| Malpighiales | Passifloraceae | 1 | 27 | 4 | 1 | 1035 | 0 |
| Malpighiales | Peraceae | 1 | 5 | 20 | 1 | 135 | 1 |
| Malpighiales | Phyllanthaceae | 5 | 59 | 8 | 9 | 2330 | 0 |
| Malpighiales | Putranjivaceae | 1 | 3 | 33 | 1 | 210 | 0 |
| Malpighiales | Rhizophoraceae | 1 | 16 | 6 | 1 | 149 | 1 |
| Malpighiales | Salicaceae | 1 | 54 | 2 | 1 | 1200 | 0 |
| Malvales | Cistaceae | 2 | 8 | 25 | 4 | 270 | 1 |
| Malvales | Cytinaceae | 1 | 2 | 50 | 1 | 10 | 10 |
| Malvales | Dipterocarpaceae | 1 | 17 | 6 | 1 | 680 | 0 |
| Malvales | Malvaceae | 17 | 243 | 7 | 22 | 4225 | 1 |
| Marsileales | Salviniaceae | 1 | 2 | 50 | 1 | 21 | 5 |
| Myrtales | Combretaceae | 3 | 14 | 21 | 20 | 500 | 4 |
| Myrtales | Lythraceae | 2 | 31 | 6 | 2 | 650 | 0 |
| Myrtales | Melastomataceae | 1 | 188 | 1 | 1 | 4960 | 0 |
| Myrtales | Myrtaceae | 16 | 131 | 12 | 37 | 5900 | 1 |
| Myrtales | Onagraceae | 1 | 22 | 5 | 1 | 656 | 0 |
| Nymphaeales | Nymphaeaceae | 1 | 3 | 33 | 1 | 58 | 2 |
| Oxalidales | Connaraceae | 1 | 12 | 8 | 1 | 180 | 1 |
| Oxalidales | Elaeocarpaceae | 1 | 12 | 8 | 1 | 635 | 0 |
| Pandanales | Pandanaceae | 1 | 5 | 20 | 1 | 885 | 0 |
| Pinales | Pinaceae | 2 | 11 | 18 | 5 | 231 | 2 |
| Piperales | Aristolochiaceae | 1 | 7 | 14 | 2 | 587 | 0 |
| Piperales | Hydnoraceae | 1 | 2 | 50 | 1 | 15 | 7 |
| Piperales | Piperaceae | 2 | 5 | 40 | 11 | 3615 | 0 |
| Poales | Cyperaceae | 1 | 98 | 1 | 3 | 5695 | 0 |
| Poales | Poaceae | 6 | 707 | 1 | 10 | 11337 | 0 |
| Poales | Typhaceae | 1 | 2 | 50 | 1 | 25 | 4 |
| Polypodiales | Pteridaceae | 1 | 52 | 2 | 1 | 1210 | 0 |
| Polypodiales | Polypodiaceae | 1 | 63 | 2 | 1 | 1650 | 0 |
| Proteales | Nelumbonaceae | 1 | 1 | 100 | 1 | 1 | 100 |
| Proteales | Sabiaceae | 1 | 4 | 25 | 1 | 199 | 1 |
| Ranunculales | Berberidaceae | 1 | 14 | 7 | 3 | 701 | 0 |
| Ranunculales | Menispermaceae | 3 | 71 | 4 | 5 | 442 | 1 |
| Ranunculales | Papaveraceae | 1 | 44 | 2 | 2 | 825 | 0 |
| Ranunculales | Ranunculaceae | 3 | 62 | 5 | 3 | 2525 | 0 |
| Rosales | Cannabaceae | 1 | 10 | 10 | 1 | 117 | 1 |
| Rosales | Elaeagnaceae | 1 | 3 | 33 | 1 | 45 | 2 |
| Rosales | Moraceae | 3 | 39 | 8 | 11 | 1137 | 1 |
| Rosales | Rhamnaceae | 1 | 52 | 2 | 3 | 1055 | 0 |
| Rosales | Rosaceae | 7 | 92 | 8 | 8 | 2805 | 0 |
| Rosales | Ulmaceae | 1 | 5 | 20 | 1 | 35 | 3 |
| Rosales | Urticaceae | 3 | 54 | 6 | 3 | 2625 | 0 |
| Santalales | Balanophoraceae | 1 | 17 | 6 | 1 | 44 | 2 |
| Santalales | Loranthaceae | 2 | 77 | 3 | 2 | 950 | 0 |
| Santalales | Olacaceae | 2 | 3 | 67 | 2 | 57 | 4 |
| Santalales | Santalaceae | 4 | 44 | 9 | 4 | 990 | 0 |
| Sapindales | Anacardiaceae | 20 | 80 | 25 | 33 | 873 | 4 |
| Sapindales | Burseraceae | 4 | 19 | 21 | 7 | 775 | 1 |
| Sapindales | Meliaceae | 6 | 50 | 12 | 7 | 641 | 1 |
| Sapindales | Rutaceae | 12 | 161 | 7 | 18 | 2085 | 1 |
| Sapindales | Sapindaceae | 5 | 144 | 3 | 7 | 1925 | 0 |
| Saxifragales | Crassulaceae | 5 | 34 | 15 | 5 | 1400 | 0 |
| Saxifragales | Paeoniaceae | 1 | 1 | 100 | 1 | 33 | 3 |
| Saxifragales | Saxifragaceae | 1 | 33 | 3 | 1 | 695 | 0 |
| Schizaeales | Lygodiaceae | 1 | 1 | 100 | 1 | 25 | 4 |
| Solanales | Convolvulaceae | 2 | 59 | 3 | 4 | 1880 | 0 |
| Solanales | Solanaceae | 3 | 102 | 3 | 9 | 2480 | 0 |
| Theaceae | Theaceae | 2 | 9 | 22 | 3 | 195 | 2 |
| Vitales | Vitaceae | 1 | 17 | 6 | 2 | 955 | 0 |
| Zingiberales | Zingiberaceae | 7 | 56 | 13 | 19 | 1600 | 1 |

**Supplementary Material S2.2** List of plant species (from **Supplementary Material S1**) tested in clinical trials for infectious and inflammatory disorders (NLM, 2020).

| Botanical Taxa | Nº Clinical Trials | Indications |
| --- | --- | --- |
| **Acanthaceae** |  |  |
| *Andrographis paniculata* (Burm.f.) Nees | 2 | Acute upper respiratory infection |
| **Adoxaceae** |  |  |
| *Sambucus nigra* L. | 4 | Otitis. Influenza. Acute respiratory illness |
| **Amaryllidaceae** |  |  |
| *Allium cepa* L. | 1 | C-section scar |
| *Allium sativum* L. | 6 | Stomatitis. Periodontitis. Upper respiratory tract infections. HIV infections |
| **Anacardiaceae** |  |  |
| *Mangifera indica* L. | 2 | Inflammatory bowel disease. Gut microbiota |
| *Schinus terebinthifolia* Raddi | 2 | Gingivitis. *Helicobacter pylori* eradication. |
| **Annonaceae** |  |  |
| *Annona muricata* L. | 1 | HPV infection |
| **Apiaceae** |  |  |
| *Petroselinum crispum* (Mill.) Fuss | 1 | Common cold symptoms |
| *Pimpinella anisum* L*.* | 1 | Oral mucositis |
| **Apocynaceae** |  |  |
| *Holarrhena pubescens* Wall. ex G.Don | 1 | Chronic diarrhea |
| **Asphodelaceae** |  |  |
| *Aloe vera* (L.) Burm.f. | 19 | Traumatic wounds. Chronic periodontitis. Ulcerative colitis. Rhinitis. Bacterial vaginosis. Cervical lesions caused by HPV |
| **Asteraceae** |  |  |
| *Baccharis dracunculifolia* DC. | 1 | Plaque and gingivitis |
| *Chamaemelum nobile* (L.) All. | 1 | Oral Lichen Planus |
| *Echinacea purpurea* (L.) Moench | 22 | Respiratory tract infections. Recurrent otitis. Common cold. Gingivitis. |
| *Matricaria chamomilla* L. | 7 | Oral mucositis. Phlebitis. Oral Lichen Planus. Oral apthae. Chronic periodontitis. Acute otitis |
| *Silybum marianum* (L.) Gaertn. | 9 | Chronic hepatitis C. Acute hepatitis. Small intestinal bacterial overgrowth |
| *Stevia rebaudiana* (Bertoni) Bertoni | 3 | Caries prevention |
| *Taraxacum campylodes* G.E.Haglund | 2 | Eczema. Acute upper respiratory infection |
| **Berberidaceae** |  |  |
| *Berberis aristata* DC. | 1 | Chronic diarrhea |
| **Burseraceae** |  |  |
| *Commiphora myrrha* (Nees) Engl. | 1 | Schistosomiasis |
| **Caricaceae** |  |  |
| *Carica papaya* L. | 4 | Impetigo. Wound inflammation. Common cold symptoms |
| **Convolvulaceae** |  |  |
| *Ipomoea batatas* (L.) Lam. | 2 | Microbiome |
| **Ericaceae** |  |  |
| *Vaccinium macrocarpon* Aiton | 40 | Urinary tract infections. Burn. Vaginitis. Common cold |
| **Euphorbiaceae** |  |  |
| *Ricinus communis* L. | 4 | Solution for cleaning dentures |
| **Fabaceae** |  |  |
| *Ceratonia siliqua* L. | 2 | Acute diarrhea. Aphtostomatitis. Mucositis |
| *Glycyrrhiza glabra* L. | 17 | Caries prevention. Postoperative Sore Throat and Postextubation Coughing. Oral Lichen. Antiplaque. Gingivitis. Chronic Rhinitis and Sinusitis. Ulcerative Colitis. Tuberculosis. Allergic rhinitis |
| **Geraniaceae** |  |  |
| *Pelargonium graveolens* L'Hér. | 1 | Allergic rhinitis |
| **Hypericaceae** |  |  |
| *Hypericum perforatum* L. | 2 | Skin damage |
| **Lamiaceae** |  |  |
| *Lavandula angustifolia* Mill. | 1 | Oral malodor |
| *Mentha piperita* L. | 5 | Acute Tracheitis and Laryngitis. Acute Rhino-Sinusitis. Plaque disclosing |
| *Mentha spicata* L. | 1 | Plaque disclosing |
| *Origanum vulgare* L. | 2 | Atopic dermatitis. Microbiome |
| *Rosmarinus officinalis* L. | 6 | Urinary Tract infections. Allergic rhinitis. Acute Tracheitis and Laryngitis. Acute Pharyngo-Tonsillitis |
| *Salvia miltiorrhiza* Bunge. | 2 | Ulcerative colitis |
| *Thymus vulgaris* L. | 6 | Cough. Common cold. Oral mucositis. Psoriasis. Acute sore throat |
| **Lauraceae** |  |  |
| *Cinnamomum cassia* (L.) J.Presl | 1 | Rhinitis |
| *Cinnamomum verum* J.Presl | 3 | Gingivitis. Rhinitis |
| **Lythraceae** |  |  |
| *Punica granatum* L. | 12 | Gingivitis. Oral Lichen Planus. Rhinovirus infection. Chronic rhinosinusitis. Inflammatory Bowel disease |
| **Malvaceae** |  |  |
| *Theobroma cacao* L. | 1 | Gut permeability |
| **Meliaceae** |  |  |
| *Azadirachta indica* A.Juss. | 9 | Acne vulgaris. Oral mucositis. Cervical lesions caused by HPV. Herpes Simplex Labialis |
| **Moringaceae** |  |  |
| *Moringa oleifera* Lam. | 2 | HIV infection |
| **Myrtaceae** |  |  |
| *Eucalyptus globulus* Labill. | 5 | Rhinitis. Acute Tracheitis and Laryngitis. Acute Pharyngo-Tonsillitis. Plaque disclosing. |
| *Leptospermum scoparium* J.R.Forst. & G.Forst. | 3 | Wound care |
| *Melaleuca alternifolia* (Maiden & Betche) Cheel | 6 | Facial Acne. Chronic blepharitis. Plaque formation |
| *Psidium guajava* L. | 1 | Caries prevention |
| *Syzygium aromaticum* (L.) Merr. & L.M.Perry | 4 | Oral mucositis. Caries prevention. Plaque disclosing |
| **Paeoniaceae** |  |  |
| *Paeonia lactiflora* Pall. | 1 | Allergic rhinitis |
| **Piperaceae** |  |  |
| *Piper betle* L. | 1 | Plaque disclosing |
| **Ranunculaceae** |  |  |
| *Nigella sativa* L. | 1 | Chronic periodontitis |
| **Rutaceae** |  |  |
| *Citrus x aurantiifolia* (Christm.) Swingle | 1 | Dental mouthwash |
| *Citrus x limon* (L.) Osbeck | 1 | Acute bronchitis |
| *Citrus sinensis* (L.) Osbeck | 2 | Hepatitis C |
| *Murraya koenigii* (L.) Spreng. | 1 | Irritable Bowel Syndrome |
| **Salvadoraceae** |  |  |
| *Salvadora persica* L. | 6 | Chronic periodontitis. Antiplaque |
| **Solanaceae** |  |  |
| *Capsicum annuum* L. | 1 | Microbiome |
| **Theaceae** |  |  |
| *Camellia sinensis* (L.) Kuntze | 2 | Oral malodor. Oral Lichen Planus |
| **Zingiberaceae** |  |  |
| *Boesenbergia rotunda* (L.) Mansf. | 1 | Mucositis |
| *Curcuma longa* L. | 10 | Chronic periodontitis. Oral Lichen Planus. Ulcerative colitis. HIV infection |
| *Zingiber montanum* (J.Koenig) Link ex A.Dietr. | 1 | Allergic rhinitis |

**Supplementary Material S2.3** Descriptive statistics for MIC data against all bacteria for crude plant extracts by botanical family**.** This represents a total of 3,498 MIC values (µg/mL) reported for extracts from species in 131 plant families.

| Botanical Family | Nº of MIC Values | Min. MIC | Max. MIC | Range | Mean | Std. Dev. | Std. Error of Mean |
| --- | --- | --- | --- | --- | --- | --- | --- |
| **Top 15 Botanical Families with Reported Antibacterial Activity** | | | | | | | |
| Anacardiaceae | 139 | 3.125 | 500 | 496.9 | 239.6 | 149.1 | 12.65 |
| Annonaceae | 72 | 7.5 | 500 | 492.5 | 200.5 | 121.5 | 14.32 |
| Apiaceae | 159 | 2.5 | 500 | 497.5 | 187.4 | 127.3 | 10.1 |
| Apocynaceae | 51 | 31 | 500 | 469 | 242.5 | 139 | 19.46 |
| Asteraceae | 165 | 3.12 | 500 | 496.9 | 211 | 154.8 | 12.05 |
| Combretaceae | 92 | 5 | 500 | 495 | 210.4 | 175.3 | 18.28 |
| Euphorbiaceae | 203 | 8 | 500 | 492 | 225 | 139.9 | 9.82 |
| Fabaceae | 391 | 3.9 | 500 | 496.1 | 210.3 | 127.9 | 6.47 |
| Lamiaceae | 165 | 0.5 | 500 | 499.5 | 216.4 | 123.6 | 9.622 |
| Lauraceae | 46 | 16 | 500 | 484 | 182.3 | 114.4 | 16.87 |
| Malvaceae | 68 | 9.18 | 500 | 490.8 | 231.5 | 126 | 15.28 |
| Myrtaceae | 112 | 4 | 500 | 496 | 180.3 | 152.3 | 14.39 |
| Rubiaceae | 131 | 12 | 500 | 488 | 185 | 125.7 | 10.98 |
| Rutaceae | 74 | 8 | 500 | 492 | 165.6 | 130.1 | 15.12 |
| Zingiberaceae | 120 | 5.92 | 500 | 494.1 | 91.79 | 123.8 | 11.3 |
| **All other Botanical Families with Reported Antibacterial Activity** | | | | | | | |
| Acanthaceae | 8 | 62.5 | 500 | 437.5 | 368 | 161.1 | 56.96 |
| Adoxaceae | 9 | 1.9 | 250 | 248.1 | 113.1 | 129.9 | 43.29 |
| Aizoaceae | 2 | 50 | 50 | 0 | 50 | 0 | 0 |
| Amaranthaceae | 26 | 31 | 500 | 469 | 235.1 | 144.8 | 28.39 |
| Amaryllidaceae | 12 | 42 | 500 | 458 | 267.8 | 141.2 | 40.77 |
| Araceae | 8 | 256 | 500 | 244 | 324.1 | 110.3 | 39.01 |
| Araliaceae | 10 | 31 | 256 | 225 | 130.5 | 75.06 | 23.74 |
| Arecaceae | 11 | 156.3 | 426.6 | 270.4 | 256.4 | 118 | 35.59 |
| Aristolochiaceae | 5 | 250 | 500 | 250 | 450 | 111.8 | 50 |
| Asparagaceae | 20 | 39 | 312.5 | 273.5 | 198.9 | 110.9 | 24.8 |
| Asphodelaceae | 42 | 10 | 500 | 490 | 113.1 | 120.4 | 18.58 |
| Balanophoraceae | 2 | 250 | 310 | 60 | 280 | 42.43 | 30 |
| Berberidaceae | 14 | 25 | 333 | 308 | 171.4 | 96.93 | 25.91 |
| Betulaceae | 1 | 256 | 256 | 0 | 256 | 0 | 0 |
| Bignoniaceae | 20 | 125 | 500 | 375 | 208.1 | 116 | 25.95 |
| Boraginaceae | 7 | 19.2 | 250 | 230.8 | 74.11 | 81.17 | 30.68 |
| Brassicaceae | 6 | 250 | 400 | 150 | 325 | 82.16 | 33.54 |
| Burseraceae | 23 | 50 | 256 | 206 | 166.4 | 86.68 | 18.08 |
| Calophyllaceae | 4 | 15.6 | 500 | 484.4 | 207 | 220 | 110 |
| Canellaceae | 7 | 100 | 500 | 400 | 401.4 | 151.7 | 57.34 |
| Cannabaceae | 2 | 31 | 62 | 31 | 46.5 | 21.92 | 15.5 |
| Capparaceae | 75 | 110.9 | 390 | 279.1 | 214.3 | 59.53 | 6.874 |
| Caricaceae | 12 | 6.25 | 500 | 493.8 | 213.9 | 197.3 | 56.97 |
| Caryocaraceae | 5 | 270 | 270 | 0 | 270 | 0 | 0 |
| Celastraceae | 15 | 100 | 500 | 400 | 302.7 | 136.4 | 35.22 |
| Chrysobalanaceae | 6 | 32 | 500 | 468 | 194.3 | 179.3 | 73.18 |
| Cistaceae | 1 | 260 | 260 | 0 | 260 | 0 | 0 |
| Cleomaceae | 9 | 90 | 500 | 410 | 292.2 | 176.1 | 58.71 |
| Clusiaceae | 63 | 14 | 500 | 486 | 176.6 | 145.1 | 18.29 |
| Connaraceae | 4 | 15.6 | 250 | 234.4 | 132.8 | 135.3 | 67.67 |
| Convolvulaceae | 18 | 40 | 500 | 460 | 268.2 | 143.4 | 33.79 |
| Crassulaceae | 18 | 62 | 500 | 438 | 205.2 | 141.6 | 33.39 |
| Cucurbitaceae | 38 | 3 | 256 | 253 | 133.8 | 94.58 | 15.34 |
| Cupressaceae | 3 | 31.3 | 256 | 224.7 | 116.6 | 121.7 | 70.28 |
| Curtisiaceae | 18 | 19.5 | 312.5 | 293 | 151.1 | 116.9 | 27.55 |
| Cyperaceae | 1 | 256 | 256 | 0 | 256 | 0 | 0 |
| Dilleniaceae | 4 | 62.5 | 500 | 437.5 | 187.5 | 210.4 | 105.2 |
| Dioscoreaceae | 7 | 64 | 250 | 186 | 99.71 | 70.43 | 26.62 |
| Dipterocarpaceae | 2 | 256 | 256 | 0 | 256 | 0 | 0 |
| Droseraceae | 3 | 300 | 450 | 150 | 366.7 | 76.38 | 44.1 |
| Ebenaceae | 24 | 3.13 | 500 | 496.9 | 105 | 120.4 | 24.57 |
| Elaeocarpaceae | 2 | 62.5 | 250 | 187.5 | 156.3 | 132.6 | 93.75 |
| Ephedraceae | 6 | 230 | 500 | 270 | 330 | 131.9 | 53.85 |
| Ericaceae | 9 | 3.9 | 500 | 496.1 | 304.5 | 235.2 | 78.4 |
| Fagaceae | 25 | 0.25 | 310 | 309.8 | 74.51 | 104.1 | 20.82 |
| Gentianaceae | 4 | 64 | 128 | 64 | 112 | 32 | 16 |
| Geraniaceae | 3 | 156 | 312 | 156 | 260 | 90.07 | 52 |
| Gunneraceae | 12 | 30 | 500 | 470 | 303.3 | 148.6 | 42.89 |
| Hernandiaceae | 2 | 125 | 250 | 125 | 187.5 | 88.39 | 62.5 |
| Hydnoraceae | 4 | 62 | 312 | 250 | 191 | 139.9 | 69.94 |
| Hypericaceae | 47 | 0.625 | 500 | 499.4 | 230.5 | 152.3 | 22.21 |
| Hypoxidaceae | 10 | 49 | 500 | 451 | 305.4 | 126.8 | 40.09 |
| Icacinaceae | 3 | 100 | 390 | 290 | 226.7 | 148.4 | 85.7 |
| Iridaceae | 16 | 10 | 250 | 240 | 107.8 | 78.87 | 19.72 |
| Juglandaceae | 2 | 310 | 310 | 0 | 310 | 0 | 0 |
| Lecythidaceae | 2 | 250 | 250 | 0 | 250 | 0 | 0 |
| Liliaceae | 4 | 128 | 256 | 128 | 192 | 73.9 | 36.95 |
| Loganiaceae | 4 | 160 | 320 | 160 | 240 | 92.38 | 46.19 |
| Loranthaceae | 4 | 125 | 500 | 375 | 359.5 | 179.5 | 89.74 |
| Lygodiaceae | 2 | 125 | 250 | 125 | 187.5 | 88.39 | 62.5 |
| Lythraceae | 26 | 4.4 | 313 | 308.6 | 141 | 101.2 | 19.85 |
| Magnoliaceae | 4 | 4 | 32 | 28 | 12.23 | 13.29 | 6.647 |
| Melastomataceae | 2 | 250 | 250 | 0 | 250 | 0 | 0 |
| Meliaceae | 98 | 15 | 500 | 485 | 126 | 127.7 | 12.9 |
| Melianthaceae | 12 | 100 | 500 | 400 | 279.2 | 128.7 | 37.16 |
| Menispermaceae | 17 | 7.5 | 500 | 492.5 | 191.1 | 190.9 | 46.29 |
| Moraceae | 33 | 19 | 500 | 481 | 171 | 129.8 | 22.6 |
| Moringaceae | 5 | 50 | 312 | 262 | 165.2 | 113.7 | 50.85 |
| Myricaceae | 24 | 31.25 | 500 | 468.8 | 260.6 | 159.9 | 32.63 |
| Myristicaceae | 6 | 32 | 64 | 32 | 58.67 | 13.06 | 5.333 |
| Nymphaeaceae | 9 | 30 | 310 | 280 | 154.4 | 125.6 | 41.87 |
| Olacaceae | 7 | 32 | 500 | 468 | 179.4 | 169.4 | 64.02 |
| Oleaceae | 33 | 156 | 470 | 314 | 286.2 | 78.06 | 13.59 |
| Onagraceae | 9 | 64 | 256 | 192 | 120.9 | 59.39 | 19.8 |
| Paeoniaceae | 3 | 80 | 80 | 0 | 80 | 0 | 0 |
| Pandanaceae | 2 | 125 | 250 | 125 | 187.5 | 88.39 | 62.5 |
| Papaveraceae | 1 | 256 | 256 | 0 | 256 | 0 | 0 |
| Passifloraceae | 5 | 128 | 256 | 128 | 230.4 | 57.24 | 25.6 |
| Paulowniaceae | 2 | 32 | 64 | 32 | 48 | 22.63 | 16 |
| Pedaliaceae | 12 | 0.39 | 256 | 255.6 | 93.16 | 107.9 | 31.15 |
| Peraceae | 1 | 256 | 256 | 0 | 256 | 0 | 0 |
| Phyllanthaceae | 60 | 7.3 | 500 | 492.7 | 170.1 | 147.5 | 19.04 |
| Piperaceae | 26 | 7.8 | 500 | 492.2 | 172.6 | 123.5 | 24.23 |
| Pittosporaceae | 7 | 62.5 | 250 | 187.5 | 133.9 | 56.23 | 21.25 |
| Plantaginaceae | 1 | 75 | 75 | 0 | 75 | 0 | 0 |
| Plumbaginaceae | 12 | 7.81 | 250 | 242.2 | 91.74 | 85.9 | 24.8 |
| Poaceae | 11 | 60 | 500 | 440 | 275.7 | 138.7 | 41.82 |
| Polygonaceae | 11 | 128 | 500 | 372 | 231.6 | 108.9 | 32.83 |
| Primulaceae | 22 | 64 | 380 | 316 | 213.3 | 94 | 20.04 |
| Pteridaceae | 4 | 20 | 500 | 480 | 317.5 | 230.7 | 115.4 |
| Putranjivaceae | 4 | 64 | 128 | 64 | 96 | 36.95 | 18.48 |
| Ranunculaceae | 7 | 20 | 500 | 480 | 298.4 | 166.1 | 62.77 |
| Rhamnaceae | 23 | 156 | 500 | 344 | 287.3 | 114.5 | 23.87 |
| Rhizophoraceae | 1 | 63 | 63 | 0 | 63 | 0 | 0 |
| Rosaceae | 66 | 15.6 | 390 | 374.4 | 227 | 101.5 | 12.49 |
| Sabiaceae | 2 | 32 | 64 | 32 | 48 | 22.63 | 16 |
| Salicaceae | 2 | 125 | 125 | 0 | 125 | 0 | 0 |
| Salvadoraceae | 14 | 31 | 250 | 219 | 115.1 | 93.05 | 24.87 |
| Salviniaceae | 1 | 300 | 300 | 0 | 300 | 0 | 0 |
| Santalaceae | 11 | 50 | 256 | 206 | 197.3 | 75.36 | 22.72 |
| Sapindaceae | 32 | 31.25 | 500 | 468.8 | 174.5 | 117.9 | 20.85 |
| Sapotaceae | 6 | 45 | 390 | 345 | 213.7 | 124.2 | 50.7 |
| Saxifragaceae | 8 | 4 | 64 | 60 | 22.5 | 19.82 | 7.008 |
| Schisandraceae | 2 | 64 | 256 | 192 | 160 | 135.8 | 96 |
| Scrophulariaceae | 20 | 64 | 500 | 436 | 287.4 | 125.8 | 28.13 |
| Solanaceae | 25 | 6.25 | 500 | 493.8 | 234.7 | 176.2 | 35.23 |
| Talinaceae | 1 | 250 | 250 | 0 | 250 | 0 | 0 |
| Tamaricaceae | 24 | 146 | 146 | 0 | 146 | 0 | 0 |
| Theaceae | 12 | 16 | 500 | 484 | 210.3 | 160.7 | 46.4 |
| Typhaceae | 1 | 312.5 | 312.5 | 0 | 312.5 | 0 | 0 |
| Ulmaceae | 1 | 62.5 | 62.5 | 0 | 62.5 | 0 | 0 |
| Urticaceae | 19 | 7.81 | 256 | 248.2 | 165.6 | 95.73 | 21.96 |
| Verbenaceae | 26 | 8 | 500 | 492 | 309.3 | 178.4 | 35 |
| Vitaceae | 3 | 210 | 500 | 290 | 376.7 | 149.8 | 86.47 |
| Winteraceae | 6 | 62.5 | 500 | 437.5 | 343.8 | 184.3 | 75.26 |
| Xanthorrhoeaceae | 2 | 30 | 40 | 10 | 35 | 7.071 | 5 |

**Supplementary Material S2.4** Descriptive statistics for MIC data on extract types against all bacteria**.** MIC values are in µg/mL.

| **Extraction Type** | **Nº of MIC Values** | **Min. MIC** | **Max. MIC** | **Range** | **Mean** | **Std. Deviation** | **Std. Error of Mean** |
| --- | --- | --- | --- | --- | --- | --- | --- |
| **Top 10 Extraction Types with Reported Antibacterial Activity** | | | | | | | |
| Acetone | 341 | 3.48 | 470 | 466.5 | 180.5 | 121.1 | 6.557 |
| Aqueous | 267 | 0.5 | 500 | 499.5 | 226.8 | 173.8 | 10.64 |
| Chloroform | 103 | 8 | 500 | 492 | 218.4 | 146.9 | 14.48 |
| Dichloromethane | 250 | 7.5 | 500 | 492.5 | 263.9 | 172.1 | 10.88 |
| Essential Oil | 881 | 0.001 | 500 | 500 | 160.1 | 154 | 5.189 |
| Ethanol | 719 | 0.25 | 500 | 499.8 | 182.7 | 128.7 | 4.8 |
| Ethyl Acetate | 199 | 1 | 500 | 499 | 162.7 | 141.4 | 10.02 |
| Fraction | 218 | 2 | 500 | 498 | 131.4 | 129.6 | 8.778 |
| Hexane | 154 | 3.13 | 500 | 496.9 | 174.4 | 139.7 | 11.26 |
| Methanol | 1227 | 0.5 | 500 | 499.5 | 191.3 | 124.9 | 3.565 |
| Other | 480 | 0.39 | 500 | 499.6 | 220.8 | 149.7 | 6.833 |
| **All other Extraction Types with Reported Antibacterial Activity** | | | | | | | |
| CA/E | 3 | 200 | 500 | 300 | 300 | 173.2 | 100 |
| CAcet/M/A (2:2:1) | 1 | 130 | 130 | 0 | 130 | 0 | 0 |
| CBenz | 1 | 63 | 63 | 0 | 63 | 0 | 0 |
| CBuOH | 19 | 64 | 390 | 326 | 209.3 | 81.33 | 18.66 |
| CCyclo | 7 | 58 | 390 | 332 | 141.9 | 118.3 | 44.71 |
| CDichl/M | 15 | 30 | 500 | 470 | 279.3 | 171.5 | 44.28 |
| CDichl/M (1:1) | 4 | 40 | 500 | 460 | 250 | 213.1 | 106.5 |
| CDiethEth | 2 | 310 | 500 | 190 | 405 | 134.4 | 95 |
| CE (ethanolic phase) | 2 | 62.5 | 500 | 437.5 | 281.3 | 309.4 | 218.8 |
| CE (oily phase) | 2 | 15.6 | 250 | 234.4 | 132.8 | 165.7 | 117.2 |
| CEtOAc/E (1:1) | 3 | 200 | 400 | 200 | 333.3 | 115.5 | 66.67 |
| CHex/EtOAc (6:4) | 4 | 80 | 310 | 230 | 157.5 | 108.4 | 54.22 |
| CHex/EtOAc (7:3) | 4 | 80 | 310 | 230 | 177.5 | 96.05 | 48.02 |
| CHex/EtOAc (8:2) | 6 | 40 | 310 | 270 | 163.3 | 120.1 | 49.04 |
| CM/Acet | 17 | 290 | 390 | 100 | 331.2 | 50.73 | 12.3 |
| CM/Chl (1:1) | 11 | 30 | 500 | 470 | 262.7 | 179 | 53.97 |
| CM/Dichl | 27 | 23 | 500 | 477 | 284.5 | 140.4 | 27.02 |
| CM/Dichl (1:1) | 66 | 20 | 500 | 480 | 288.9 | 140 | 17.24 |
| CPEt | 44 | 0.39 | 400 | 399.6 | 161.4 | 128.6 | 19.38 |
| None | 61 | 6.25 | 500 | 493.8 | 111.7 | 135.8 | 17.39 |
| NS | 21 | 7.81 | 500 | 492.2 | 254.1 | 192.2 | 41.95 |
| Pa. | 54 | 7.8 | 500 | 492.2 | 219.7 | 119.4 | 16.25 |
| Pyrolyzer | 2 | 250 | 500 | 250 | 375 | 176.8 | 125 |
| Ultrasound-Extraction | 1 | 125 | 125 | 0 | 125 | 0 | 0 |

**Legend:** A: Aqueous, Acet: Acetone, Benz: Benzene, BuOH: Butanol, C: Crude extract, Chl: Chloroform, Cyclo: Cyclohexane, Dichl: Dichloromethane, DiethEth: Diethylether extract, E: Ethanol, EtOAc: Ethyl acetate, Hex: Hexane, M: Methanol, Pa.: Partition, PEt: Petroleum ether.

**Supplementary Material S2.5** Descriptive statistics for the MIC values (**µg/mL)** against all bacteria, sorted by plant tissue.

| Plant tissue | Nº of MIC Values | Min. MIC | Max. MIC | Range | Mean | Std. Deviation | Std. Error of Mean | |
| --- | --- | --- | --- | --- | --- | --- | --- | --- |
| **Top 10 Plant Tissues with Reported Antibacterial Activity** | | | | | | | |  |
| Aerial part | 601 | 0.312 | 500 | 499.7 | 142.7 | 134.7 | 5.493 | |
| Bark | 316 | 3.125 | 500 | 496.9 | 235.1 | 149.9 | 8.434 | |
| Fruit | 239 | 1.9 | 500 | 498.1 | 161.8 | 135.8 | 8.785 | |
| Leaf | 1601 | 0.001 | 500 | 500 | 208.3 | 145.2 | 3.628 | |
| Rhizome | 115 | 5.92 | 500 | 494.1 | 125.3 | 162.9 | 15.19 | |
| Root | 370 | 2.8 | 500 | 497.2 | 210.5 | 136.1 | 7.073 | |
| Seed | 218 | 5 | 500 | 495 | 189.1 | 115.4 | 7.818 | |
| Stem | 176 | 0.25 | 500 | 499.8 | 170 | 143.4 | 10.81 | |
| Stem bark | 116 | 3.13 | 460 | 456.9 | 156 | 101 | 9.378 | |
| Whole plant | 123 | 0.39 | 500 | 499.6 | 206.9 | 140.3 | 12.65 | |
| Other | 861 | 0.03 | 500 | 500 | 174.2 | 143.8 | 4.901 | |
| **All Other Plant Tissues with Reported Antibacterial Activity** | | | | | | | |  |
| Aerial part, root | 3 | 10 | 70 | 60 | 36.67 | 30.55 | 17.64 | |
| Bark, leaf | 4 | 260 | 420 | 160 | 340 | 69.76 | 34.88 | |
| Bark, root, leaf | 17 | 62.5 | 500 | 437.5 | 308.8 | 176 | 42.7 | |
| Bean | 10 | 64 | 256 | 192 | 179.2 | 84.26 | 26.65 | |
| Berry | 1 | 23 | 23 | 0 | 23 | 0 | 0 | |
| Blooming herb | 3 | 50 | 400 | 350 | 183.3 | 189.3 | 109.3 | |
| Branch | 3 | 50 | 500 | 450 | 200 | 259.8 | 150 | |
| Branch, leaf | 25 | 8 | 500 | 492 | 104.2 | 124.4 | 24.87 | |
| Branchlet | 5 | 70 | 250 | 180 | 148 | 67.23 | 30.07 | |
| Bud | 4 | 100 | 500 | 400 | 375 | 189.3 | 94.65 | |
| Bulb | 63 | 4 | 390 | 386 | 148.9 | 111.5 | 14.05 | |
| Calyx | 7 | 0.038 | 9.18 | 9.142 | 1.391 | 3.435 | 1.298 | |
| Capitula | 1 | 500 | 500 | 0 | 500 | 0 | 0 | |
| Clove | 1 | 64 | 64 | 0 | 64 | 0 | 0 | |
| Corm | 7 | 156 | 500 | 344 | 330 | 110.6 | 41.81 | |
| Corn | 1 | 30 | 30 | 0 | 30 | 0 | 0 | |
| Flower | 83 | 6 | 450 | 444 | 142.8 | 114.3 | 12.54 | |
| Flower head | 7 | 125 | 500 | 375 | 321.4 | 174.7 | 66.02 | |
| Flowering aerial part | 4 | 100 | 400 | 300 | 275 | 150 | 75 | |
| Flowering shoot | 1 | 500 | 500 | 0 | 500 | 0 | 0 | |
| Fruit (juice) | 1 | 313 | 313 | 0 | 313 | 0 | 0 | |
| Fruit (peel) | 4 | 100 | 200 | 100 | 175 | 50 | 25 | |
| Fruit (pulp) | 2 | 100 | 190 | 90 | 145 | 63.64 | 45 | |
| Fruit (ripe) | 6 | 50 | 150 | 100 | 91.67 | 37.64 | 15.37 | |
| Fruit (syrup) | 5 | 313 | 313 | 0 | 313 | 0 | 0 | |
| Fruit (unripe) | 6 | 100 | 200 | 100 | 133.3 | 40.82 | 16.67 | |
| Fruit husk | 1 | 256 | 256 | 0 | 256 | 0 | 0 | |
| Fruit peel | 16 | 4.4 | 256 | 251.6 | 114.3 | 88.24 | 22.06 | |
| Gall | 4 | 160 | 310 | 150 | 235 | 86.6 | 43.3 | |
| Gel | 6 | 31.25 | 500 | 468.8 | 140.6 | 178.7 | 72.95 | |
| Heartwood | 18 | 31.3 | 500 | 468.7 | 161.5 | 119.5 | 28.16 | |
| Herb | 5 | 50 | 400 | 350 | 150 | 154.1 | 68.92 | |
| Inflorescence | 24 | 7.81 | 500 | 492.2 | 175 | 143 | 29.19 | |
| Inner bark | 1 | 62.5 | 62.5 | 0 | 62.5 | 0 | 0 | |
| Juice | 2 | 20 | 25 | 5 | 22.5 | 3.536 | 2.5 | |
| Latex | 7 | 31.25 | 500 | 468.8 | 185.5 | 217.6 | 82.23 | |
| Latex (unripe fruit) | 7 | 19 | 312 | 293 | 116.9 | 134.8 | 50.93 | |
| Leaf (fermented) | 1 | 500 | 500 | 0 | 500 | 0 | 0 | |
| Leaf (bud) | 1 | 295.5 | 295.5 | 0 | 295.5 | 0 | 0 | |
| Leaf (gel) | 1 | 200 | 200 | 0 | 200 | 0 | 0 | |
| Leaf (latex) | 11 | 10 | 50 | 40 | 25.45 | 13.87 | 4.181 | |
| Leaf (non fermented) | 1 | 200 | 200 | 0 | 200 | 0 | 0 | |
| Leaf (semi fermented) | 1 | 200 | 200 | 0 | 200 | 0 | 0 | |
| Leaf, stem | 19 | 50 | 312.5 | 262.5 | 202.4 | 106.3 | 24.38 | |
| Leaf, flower | 3 | 50 | 200 | 150 | 150 | 86.6 | 50 | |
| Leaf, fruit peel | 1 | 500 | 500 | 0 | 500 | 0 | 0 | |
| Leaf, herbaceous branch | 1 | 12.5 | 12.5 | 0 | 12.5 | 0 | 0 | |
| Leaf, seed | 7 | 128 | 256 | 128 | 164.6 | 62.46 | 23.61 | |
| Leaf, seed, bark | 1 | 49 | 49 | 0 | 49 | 0 | 0 | |
| Leaf, stem | 1 | 130 | 130 | 0 | 130 | 0 | 0 | |
| Leaf, twig | 14 | 64 | 256 | 192 | 190.5 | 83.45 | 22.3 | |
| Leaf, whole plant | 7 | 128 | 256 | 128 | 201.1 | 68.42 | 25.86 | |
| Ligulate flower | 6 | 9.75 | 156.3 | 146.5 | 60.24 | 51.82 | 21.16 | |
| Needletip | 1 | 400 | 400 | 0 | 400 | 0 | 0 | |
| NS | 180 | 0.03 | 500 | 500 | 209.1 | 149.4 | 11.13 | |
| Nut gall | 2 | 100 | 500 | 400 | 300 | 282.8 | 200 | |
| Oleoresin | 36 | 6.25 | 400 | 393.8 | 107.1 | 123.5 | 20.59 | |
| Peel | 9 | 10 | 313 | 303 | 125.9 | 104.7 | 34.89 | |
| Pericarp | 6 | 14 | 128 | 114 | 43.33 | 45.58 | 18.61 | |
| Pomace | 17 | 290 | 390 | 100 | 331.2 | 50.73 | 12.3 | |
| Resin | 20 | 12.5 | 500 | 487.5 | 116.2 | 152.2 | 34.04 | |
| Ripe fruit | 5 | 31 | 500 | 469 | 306.2 | 198.2 | 88.65 | |
| Root bark | 18 | 1.56 | 256 | 254.4 | 93.06 | 85.06 | 20.05 | |
| Root, rhizome | 6 | 50 | 500 | 450 | 425 | 183.7 | 75 | |
| Root, stolon | 2 | 16 | 32 | 16 | 24 | 11.31 | 8 | |
| Sap | 2 | 62.5 | 125 | 62.5 | 93.75 | 44.19 | 31.25 | |
| Seed coat | 6 | 50 | 312.5 | 262.5 | 177.9 | 112.4 | 45.87 | |
| Seed husk | 4 | 160 | 160 | 0 | 160 | 0 | 0 | |
| Seed, fruit | 3 | 128 | 256 | 128 | 170.7 | 73.9 | 42.67 | |
| Shoot | 1 | 125 | 125 | 0 | 125 | 0 | 0 | |
| Stem, bark | 28 | 20 | 468.2 | 448.2 | 112.6 | 112.5 | 21.27 | |
| Trunk | 1 | 250 | 250 | 0 | 250 | 0 | 0 | |
| Trunk bark | 1 | 156 | 156 | 0 | 156 | 0 | 0 | |
| Tuber | 23 | 10 | 500 | 490 | 176 | 186.8 | 38.95 | |
| Twig | 24 | 15 | 500 | 485 | 222.1 | 136.4 | 27.85 | |
| Underground part | 1 | 250 | 250 | 0 | 250 | 0 | 0 | |
| Wood | 22 | 2 | 500 | 498 | 133.9 | 147.2 | 31.38 | |
| Wood (no bark) | 2 | 250 | 500 | 250 | 375 | 176.8 | 125 | |

# Supplementary Material S2.6. Continuation of the discussion of top performing plant families with antibacterial activity (from Section 4.4 of the main text).

### Euphorbiaceae

Members of the Euphorbiaceae frequently contain latices used in traditional medicine (Salatino et al., 2007). A total of 28 species from Euphorbiaceae were found to have antibacterial activity in the study range, with the lowest MICs being exhibited by extracts of *Croton* spp. and *Euphorbia* spp.

*Croton* is a large genus found in tropical and subtropical regions across the globe (Salatino et al., 2007). Many species of *Croton* have a red latex known as dragon’s blood that is used for wound healing; experiments with an *in vivo* rat model have shown that dragon’s blood stimulates wound contraction, crust formation and regeneration (Pieters et al., 1995). *C. lechleri* has the distinction of being the source of crofelemer, one of two FDA-approved botanical drugs (Sasisekharan et al., 2019). Crofelemer (Mytesi) is an oligomeric proanthocyanidin used to treat secretory diarrhea, acting by blocking chloride channels in the gut (Tradtrantip et al., 2010).

The root bark of *Croton campestris* A.St.-Hil. is used to treat syphilis in Brazil, and an EO of this material exhibited MIC of 1.56 μg/mL against *Staphylococcus aureus* (El Babili et al., 2012). *C. gratissimus* Burch. is a shrub native to Africa used for a variety of medicinal applications, especially relating to sexually transmitted infections and tetanus (van Vuuren and Naidoo, 2010;Cheikhyoussef et al., 2011). The aerial parts are used to treat malaria, cough and constipation; *in vitro* experiments with a monoterpene-dominated EO of the aerial parts showed MICs of 16µg/mL against *S. aureus*, *Bacillus subtilis* and *Escherichia coli* (Yagi et al., 2016). An earlier study of *C. gratissimus* found that synergy between compounds in bark, leaf and root extracts may be responsible for some of the plant’s antimicrobial activity (van Vuuren and Viljoen, 2008).

*Euphorbia* is the largest genus from the Euphorbiaceae family, containing approximately 2,000 species (Horn et al., 2012). Of the *Euphorbia* species reviewed here, only *E. macrorhiza* exhibited crude extract MICs < 100 μg/mL. *E. macrorhiza* is an herb native to central and northern Asia that has not been widely studied; the EO of its roots and aerial parts both showed MICs of 2.8 and 5.6 µg/mL against *S. aureus,* respectively (Lin et al., 2012). The root EO main constituents were acorenone B, (+)-cycloisosativene, β-cedrene and 3a-hydroxy-5b-androstane and the main constituents of the aerial part EO were acorenone B , (+)-cycloisosativene , 3α-hydroxy-5β-androstane and β-cedrene (Lin et al., 2012). Other more well-known antibacterial *Euphorbia* species include *E. prostrata*, an herb widespread in Africa and South Asia, used traditionally to treat gastrointestinal disorders (Tala et al., 2015). A methanol extract of *E. prostrata* exhibited MICs of 128-256 µg/mL against multidrug-resistant strains of *E. coli*, *Enterobacter aerogenes,* *Klebsiella pneumoniae* and *S. aureus*. Quercetin, quercetin derivatives, kaempferol, β-sitosterol and stigmasterol have been reported from this plant species and might be involved in the activity (Voukeng et al., 2017a). Several other antibacterial constituents have been isolated from *Euphorbia* species. For instance, a bioguided-fractionation of *Euphorbia drupifera* Thonn. for antibacterial activity led to the isolation of various compounds acting synergistically with each other, such as ellagic acid derivatives (including 3,3′,4′-tri-*O-*methylellagic acid, the most active one), flavonoid glycosides (including the mixture (1:1) afzelin and quercetin-3-*O-β*-_D_-xylopyranoside) and steroids (mixture of stigmasterol and *β*-sitosterol) (Voukeng et al., 2017b). An *in vivo* test of an aqueous *E. prostrata* extract for salmonellosis in rats found a dose-dependent reduction of *Salmonella typhimurium* loads (Tala et al., 2015).

### Lauraceae

Of the 26 species studied from Lauraceae, 15 were from the genus *Cinnamomum*, including the two species with the most active extracts, *Cinnamomum verum* and *Cinnamomum longipaniculatum* (Gamble) N.Chao ex H.W.Li. The spice cinnamon comes from the inner bark of *Cinnamomum* trees. Cinnamon is widely used as a seasoning and also as a treatment for conditions including respiratory, digestive and gynecological disorders.(Ranasinghe et al., 2013)

*Cinnamomum verum* (cinnamon) is native to Sri Lanka and southern India (Ranasinghe et al., 2013). The EO of *C. verum* bark has exhibited MICs of 0.63-103 μL/mL both in gram-positive bacteria such as *Staphylococcus aureus*, *Streptococcus* spp. and *Bacillus* sp. as well as gram-negative bacteria such as *Escherichia coli,* *Shigella* spp. and *Haemophilus ducreyi* (Lindeman et al., 2014;Nasir et al., 2015;Vaillancourt et al., 2018). Mechanistic studies of *C. verum* EO have found that it acts by altering the selective permeability of the bacterial membrane, destroying membrane potential and inhibiting respiration with the end result of cell death (Bouhdid et al., 2010). Given cinnamon’s common use as a spice, *in vivo* use of cinnamon as an antibacterial may have a relatively low risk; for example, a pediatric journal reported the resolution of an infant’s chronic *Salmonella enteritidis* infection after the infant was fed cinnamon (Rosti and Gastaldi, 2005). *C. verum* is named in a total of 67 clinical trials for conditions ranging from diabetes to cardiovascular disorders (NLM, 2020). A clinical trial of cinnamon extract (20% v./v.) as a twice-daily mouthwash found significant reduction in plaque and gingivitis compared to placebo at 15 and 30 day timepoints and no significant difference between cinnamon mouthwash and chlorhexidine mouthwash, the positive control (Gupta and Jain, 2015). The main constituents of *C. verum* bark EO are cinnamaldehyde, eugenol and linalool, all of which have reported antibacterial activity, including synergy between cinnamaldehyde and other natural products (Ranasinghe et al., 2013;Ye et al., 2013;Yu et al., 2015).

*Cinnamomum longipaniculatum* is endemic to China and, in contrast to *C. verum*, is generally studied for the antibacterial activity of its leaf EO (Li et al., 2014). This EO was found to inhibit growth of *S. aureus, Escherichia coli* and *Salmonella enteritidis* with a MIC of 1.25 μL/mL; antibacterial constituents were identified as eucalyptol, safrole, terpinene-4-ol, α-terpineol and γ-terpinene (Li et al., 2014).

###

### Malvaceae

Of the 22 species studied in the Malvaceae family, *Hibiscus surattensis* L., *H. sabdariffa* L., *Cienfuegosia digitata* Cav., *Dombeya rotundifolia* (Hochst.) Planch. and *Grewia flava* DC. had the strongest antibacterial activity.

*Hibiscus* is a genus of flowering plants containing hundreds of perennial herbs, trees and shrubs species. It can be found in tropical, subtropical and warm-temperate regions around the world. Several folk remedies make use of the hibiscus flower, and these medicinal effects may be a result of its polyphenolic content (Salib, 2014). *Hibiscus surattensis* (bush sorrel) is a climbing annual herb found in tropical Africa and Asia. The calyx of *H. surattensis* is used traditionally to treat malaria, hypertension, venereal diseases and ureteritis in Africa (Koudouvo et al., 2011;Gbolade, 2012). Its EO demonstrated MICs of 0.038-0.124 µg/mL against *Escherichia coli*, *S. aureus*, *Enterobacter aerogenes*, *Shigella flexneri*, *Salmonella typhimurium* and *Listeria monocytogenes*; the main EO constituents were β-caryophyllene, menthol, camphor and methyl salicylate (Akarca, 2019).

*Hibiscus sabdariffa* (roselle) is a perennial herb native to West Africa. Its calyx is used in folk medicine for the treatment of hypertension, sore throat, coughs, wounds and as a diuretic (Alarcón-Alonso et al., 2012;Da-Costa-Rocha et al., 2014). It is also used traditionally to make hibiscus herbal tea, which has been shown to lower hypertension (Haji Faraji and Haji Tarkhani, 1999). *H. sabdariffa* exhibits antibacterial activity against *Helicobacter pylori* and *Enterobacter cloacae* with MICs of 9.18 and 25 µg/mL, respectively (Djeussi et al., 2013;Hassan et al., 2016).

###

### Combretaceae

Of the 20 species studied in the Combretaceae family, ten were from the genus *Combretum*, nine from *Terminalia* and one from *Anogeissus*. The five most bioactive were *Combretum album* Pers., *Terminalia chebula* Retz., *T. phanerophlebia* Engl. & Diels, *C. micranthum* G.Don and *T. fagifolia* Mart. *C. album* and *T. chebula*; the two most bioactive are discussed below.

*Combretum album* is a small climber shrub found in South Asia and Southeast Asia. It is traditionally used for diarrhea, inflammation, digestion, diuretic problems and malarial fever (Burman et al., 2018). An ethanol extract of *C. album* leaves showed MICs of 5-20 µg/mL against *Pseudomonas aeruginosa*, *Pseudomonas putida*, *Escherichia coli*, *Bacillus licheniformis*, *B. subtilis* and *B. mycoides*; it is contains tannins, flavonoids and alkaloids (Burman et al., 2018).

*Terminalia chebula* (myrobalan) is a deciduous tree native to South Asia and parts of the Himalayas (Suguna et al., 2002). Ayurvedic medicine commonly uses *T. chebula* for asthma, vomiting, sore throat, ulcers and gout (Bag et al., 2013). *T. chebula* is also used to reduce swelling and for wounds (Bai et al., 2015). The unripe fruit is used for dysentery and diarrhea (Suguna et al., 2002). Crude extracts exhibited MICs of 7.3-31.2 µg/mL against *Pseudomonas aeruginosa*, *Proteus vulgaris*, *Klebsiella pneumoniae* and *Staphylococcus aureus* (Bai et al., 2015). Phenolics such as ellagic acid found in *T. chebula* may be responsible for this antibacterial activity (Kim et al., 2006). An *in vivo* clinical trial investigating the efficacy of using *T. chebula* for wound healing in rats showed that alcoholic extracts reduced the epithelialization period, showed faster contraction of the wound and increased tensile strength of the wound tissue. It exhibits antibacterial activity against *S. aureus* and *K. pneumoniae*, thus making it a potential candidate for treatment of infected wounds (Suguna et al., 2002).

###

### Zingiberaceae

Members of the Zingiberaceae family are mainly found in South and Southeast Asia. They are rich sources of phytochemical substances such as curcuminoids, gingerols and kava pyrones which have several biological activities (Chen et al., 2008). Of the 19 species in the Zingiberaceae family studied as antibacterials, the five most active species were *Curcuma longa* L. (**Figure 14F**), *Boesenbergia rotunda* (L.) Mansf., *Zingiber officinale* Roscoe (**Figure 14B**), *Etlingera coccinea* (Blume) S.Sakai & Nagam. and *Curcuma aromatica* Salisb. The three most active species, *C. longa*, *B. rotunda* and *Z. officinale*, are discussed below.

*Curcuma longa* (turmeric) is a perennial herb native to Southwest India. Its rhizomes have been used both as a culinary spice and in traditional Chinese and Ayurvedic medicine to treat inflammatory diseases and ailments. The rhizomes are also used traditionally to treat coughs, fever, jaundice, wounds, itching, gastrointestinal problems, asthma and throat irritation (Gilani et al., 2005). The rhizomes of *C. longa* showed MICs of 5.92-7.27 µg/mL against *Proteus mirabilis*, *Bacillus subtilis*, *Staphylococcus aureus*, *Klebsiella pneumoniae* and *Pseudomonas aeruginosa* (Chakraborty et al., 2014). Curcumin may be responsible for this antibacterial activity, along with also demethoxycurcumin, turmerone and 2,3-dihydrobenzofuran (Lim, 2016). *C. longa* is named in a total of 152 clinical trials for conditions such as chronic periodontitis, ulcerative colitis and skin inflammations (NLM, 2020). In a clinical trial evaluating 35 *Helicobacter pylori*-infected patients treated with turmeric, 77% of them tested negative for *H. pylori* after 8 weeks of treatment (Lim, 2016). In another clinical trial examining 45 patients with peptic ulcer symptoms and treated with turmeric powder, 48%, 72% and 76% did not exhibit ulcers after 4, 8 and 12 weeks respectively (Prucksunand et al., 2001). However, the effect of *C. longa* on *H. pylori* is not clear, as another clinical study did not find bactericidal effects of turmeric in patients infected by *H. pylori* (Koosirirat et al., 2010).

Within the *Curcuma* genus, several other species such as *Curcuma aromatica* also possess antibacterial activity. *C. aromatic* (wild turmeric) has been used traditionally as an anti-inflammatory agent, to promote blood circulation and to treat cancer and gynecological diseases in China (Zheng and Xing, 2009;Revathi and Malathy, 2013). The rhizomes are also used for bruises, coughs, headaches, intestinal helminths, to treat flatulence and skin infection in India (Rao, 1981;Revathi and Malathy, 2013;Daimei and Kumar, 2014). Curcumin can also be found in the ethyl acetate extracts of *C. aromatica* (Pant et al., 2013).

*Boesenbergia rotunda* (fingerroot) is a common herb native to Southeast Asia. It is traditionally used to treat rheumatism, muscle pain, gout and gastrointestinal disorders. The rhizomes are also used for dental caries, dermatitis, dry coughs, colds and wounds (Eng-Chong et al., 2012). Crude ethanol extracts of *B. rotunda* exhibited MICs of 7.81-64 µg/mL against *Streptococcus pyogenes*, *S. mutans*, *Staphylococcus epidermidis* and *S. aureus* (Limsuwan and Voravuthikunchai, 2013;Sukandar et al., 2016;Teethaisong et al., 2018). Flavonoid components of *B. rotunda* such as pinostrobin may contribute to this antibacterial activity (Eng-Chong et al., 2012). An *in vivo* study of *B. rotunda* investigating peptic ulcers in Mongolian gerbils showed decreased *H. pylori* bacterial load and decreased acute and chronic inflammation upon introduction of oral *B. rotunda* treatment (Mahady et al., 2006).

*Zingiber officinale* (ginger) is used around the world as a spice. It is also widely used as a medicine for gastrointestinal ailments, headaches, nausea, colds, inflammation and pain relief (Al-Daihan et al., 2013). *Z. officinale* exhibited MICs of 10-19.25 µg/mL against *B. subtilis*, *P. aeruginosa*, *S. aureus*, *Escherichia coli*, *K. pneumoniae* and *Proteus mirabilis* (Chakraborty et al., 2014;Chakotiya et al., 2016). Phenolic compounds such as [10]-gingerol and [12]-gingerol may be responsible for the antibacterial activity of *Z. officinale* (Park et al., 2008). An *in vivo* study of *Z. officinale* for dental caries in rats found a significant reduction in *Streptococcus mutans* and an overall reduction in smooth dental caries as well as a lack of acute oral toxicity (Hasan et al., 2015). A standardized ginger extract (100 mg/kg) administered three weeks prior to *H. pylori* challenge reduced the bacterial load as well as the acute and chronic inflammation in Mongolian gerbils (Gaus et al., 2009). *Z. officinale* is named in 158 clinical trials, particularly for its role in the treatment of nausea associated with chemotherapy and general anaesthesia (NLM, 2020). Although it has been shown to induce only minor adverse effects and can generally be considered safe, further studies are needed to confirm its efficacy as an antibacterial agent (Ali et al., 2008).

###

### Rutaceae

Of the 18 species from the Rutaceae family studied for antibacterial activity, the top five species for antibacterial activity are *Murraya koenigii* (L.) Spreng. (**Figure 14A**), *Zanthoxylum gilletii* (De Wild.) P.G.Waterman, *Skimmia arborescens* T. Anderson ex Gamble, *Citrus aurantiifolia* (Christm.) Swingle and *Ruta graveolens* L. Among these top bioactive species, *Murraya koenigii*, *Zanthoxylum gilletii* and *Citrus aurantiifolia* were selected to further discuss.

*Murraya koenigii* (curry tree) is a tree native to the Indian subcontinent. It has been used traditionally as a stomachic, stimulant, analgesic and for the treatment of dysentery, nausea and diarrhea. Its leaves and roots are also used to alleviate kidney pain and for blood disorders, inflammation and itching (Rahman and Gray, 2005). An ethyl acetate extract of *M. koenigii* leaves showed MICs of 8-16 µg/mL against *Bacillus megaterium*, *Bacillus subtilis*, *Staphylococcus aureus*, *E. coli*, *Salmonella typhi* and *Shigella dysenteriae* (Ghosh et al., 2018). Compounds such as carbazole alkaloids as well as the steroid β-stigmasterol may be responsible for the antibacterial properties of *M. koenigii* (Rahman and Gray, 2005). Quercetin, which is also found in several other species of the family Rutaceae, may also contribute to the growth inhibitory activities seen in these species (Ghasemzadeh et al., 2014). An ointment prepared with *M. koenigii* leaf ethanol extract was reported to facilitate and accelerate wound healing in rats and this was correlated with its antibacterial activity (Nagappan et al., 2012). A polyherbal mouthwash consisting of *M. koenigii*, *Acacia nilotica* (L.) Delile (Fabaceae), *Psidium guajava* (Myrtaceae) and a *Eucalyptus* (Myrtaceae) hybrid was tested in a clinical trial for dental caries and plaque-induced dental diseases; the results showed less plaque build-up and bacterial count in the human subjects that used the polyherbal mouthwash compared to the saline group and statistical similarity between the polyherbal mouthwash and the chlorhexidine positive control (Shekar et al., 2019).

*Zanthoxylum gilletii* (East Africa satinwood) is a tree native to Africa. The leaf, root and bark of *Z. gilletii* are traditionally used as antimalarial and anti-bacterial agents and for stomachaches, fever, joint pain and inflammation (Gaya et al., 2013). Crude methanol extracts showed MICs of 8-64 µg/mL against *E. coli*, *Klebsiella pneumoniae*, *Providencia stuartii*, *Pseudomonas aeruginosa* and *Enterobacter aerogenes* (Seukep et al., 2015;Tankeo et al., 2015). Terpenes such as α-pinene, borneol, linalool, δ-guaiene and camphor may be responsible for this antibacterial activity (Sinan et al., 2019).

*Citrus x aurantiifolia* (lime) is a common evergreen tree native to tropical Asia. It is traditionally used for headache, cold and diarrhea and to speed up contractions during birth (Ticktin and Dalle, 2005;Muthu et al., 2006). EO extracts of *C. aurantiifolia* showed MICs of 20-157 µg/mL against *Streptococcus mutans*, *S. mitis*, *Lactobacillus casei*, *Streptococcus salivarius*, *S. mitis* and methanol extracts showed MIC of 157 µg/mL against *B. subtilis* (Faboro et al., 2016;Lemes et al., 2018). Flavonoids also extracted from *Zanthoxylum gilletii* and *Skimmia arborescens* such as quercetin, as well as bioactive compounds isolated from the EO such as limonene, β-pinene and γ-terpinene, may be responsible for the antibacterial activity (Narang and Jiraungkoorskul, 2016). Extracts of *C. aurantiifolia* were shown to inhibit urease—a major enzyme in *Helicobacter pylori*—*in vitro*, supporting the use of *C. aurantiifolia* for gastrointestinal diseases (Nabati et al., 2012).

###

### Annonaceae

Of the 15 species studied in the Annonaceae family, five species belonged to the Annona genus. *Cleistochlamys kirkii* (Benth.) Oliv., *Uvaria caffra* E. Mey. ex Sond., *Greenwayodendron suaveolens* (Engl. & Diels) Verdc., *Annona reticulata* L. and *Annona senegalensis* Pers. were the five most bioactive species. *Annona* spp. and *Cleistochlamys kirkii* are discussed in more detail.

*Annona* is a genus of tropical fruit trees. Species from this genus have been used as traditional herbal remedies for parasites, infectious diseases, cancer and diabetes (Quílez et al., 2018). Acetogenins represent the main specific class of compounds from *Annona* species. These compounds have wide pharmacological activities, but are also involved in neurotoxicity (Liaw et al., 2016). In the genus, *Annona squamosa* L. (sweetsop) is the most studied species for its antibacterial activity. A leaf ethanol extract showed MICs of 62.5-250 µg/mL against *Staphylococcus aureus*, *Listeria monocytogenes*, *Bacillus cereus* and *Campylobacter jejuni* (Dholvitayakhun et al., 2012).

*Annona reticulata* (custard apple) is a tree native to Central America and distributed throughout the tropics. It is used to treat bacterial infections, diarrhea, fever, ulcers, worm infections and wounds (Jamkhande and Wattamwar, 2015). Ethanol extracts of its fruits and roots showed MICs of 31-250 µg/mL against *Mycobacterium smegmatis* and *M. ulcerans* (Tsouh Fokou et al., 2016).

*Annona senegalensis* (African custard apple) is a tree native to Senegal. It is used traditionally for diarrhea, headaches, eye swelling, respiratory infections and malaria. *A. senegalensis* showed MICs of 0.25-250 µg/mL against *Staphylococcus epidermidis*, *Mycobacterium smegmatis*, *Brevibacterium agri*, *Brevibacterium linens*, *Cutibacterium acnes* and *Mycobacterium ulcerans* (Nciki et al., 2016;Tsouh Fokou et al., 2016). For all *Annona* species, few data are available regarding their *in vivo* activity. Additionally, the presence of neurotoxic acetogenins in *Annona* species could lead to toxicity. For instance, a recent study suggests that a long-term consumption of *A. squamosa* could be a potential risk factor for neurodegenerative disorders (Bonneau et al., 2017).

*Cleistochlamys kirkii* (purple cluster pear) is a shrub occurring in tropical Africa. It is used traditionally to treat wound infections, tuberculosis and rheumatism (Pereira et al., 2016). Crude methanol extracts of *C. kirkii* exhibited MICs of 7.5 and 30 μg/mL against *P. aeruginosa* and *Enterococcus faecalis,* respectively. Flavone constituents such as dichamanetin have exhibited strong antibacterial activity against several gram-positive bacteria (Pereira et al., 2016).

###

### Apocynaceae

Out of the 13 species of the family Apocynaceae studied for antibacterial activity, the top five most bioactive were *Gomphocarpus fruticosus* (L.) W.T.Aiton, *Strophanthus hispidus* DC., *Tabernaemontana elegans* Stapf., *Alstonia boonei* De Wild and *Catharanthus roseus* (L.) G.Don., *Gomphocarpus fruticosus* and *Tabernaemontana elegans* showed the best antibacterial activity and are discussed in more detail.

*Gomphocarpus fruticosus* (swan milkweed) is a shrub native to South Africa. It is traditionally used to treat headaches, colds stomach pain and tuberculosis (Moteetee and van Wyk, 2011;Nyila et al., 2012). Hexane extracts of *G. fruticosus* showed MICs of 31-250 µg/mL against *Pseudomonas aeruginosa*, *Enterococcus faecalis* and *Staphylococcus aureus* (Madureira et al., 2012). Flavonoids such as quercetin may be responsible for this antibacterial activity (Heneidak et al., 2006). More research needs to be done to test the *in vivo* use of *G. fruticosus* against bacterial diseases.

*Catharanthus roseus* (Madagascar periwinkle) is an herbaceous perennial native to Madagascar. *C. roseus* is used traditionally for headaches and diabetes (Nayak and Pinto Pereira, 2006), and is famously the source of the vinca alkaloids, one of the most used of classes of anti-cancer drugs (Moudi et al., 2013). A leaf methanol extract exhibited MICs of 128-250 µg/mL against MRSA, *Escherichia coli*, *Enterobacter aerogenes* and *Pseudomonas aeruginosa* (Voukeng et al., 2016). Another study found apigenin, kaempferol and gallic acid in fractions of *C. roseus* stem methanol extract, which might be responsible for the antibacterial activity (Pham et al., 2018). An *in vivo* test of a *C. roseus* flower extract for wound healing in rats found a significant improvement relative to placebo, attributed to triterpenoids, tannins and alkaloids present in the extract (Nayak and Pinto Pereira, 2006). Although *C. roseus* is well-known for its vinca-alkaloids, other compounds seem to be involved in the antibacterial activity. The consumption of *C. roseus* extracts can be dangerous as demonstrated by case reports of adverse events (Wu et al., 2004;Nejat et al., 2015).

*Tabernaemontana elegans* (toad tree) is a shrub native to eastern Africa. It is traditionally used for chest complaints, tuberculosis and wounds (Luo et al., 2011). *T. elegans* exhibited MICs of 62.5-250 μg/mL against *Enterococcus faecalis*, *Bacillus subtilis*, *S. aureus*, *Mycobacterium tuberculosis* and *P. aeruginosa* (Madureira et al., 2012;Pallant et al., 2012). Dregamine, an indole alkaloid isolated from the roots, stems and leaves of *T. elegans*, may contribute to this antibacterial activity (van der Heijden et al., 1986). While several *in vitro* studies have confirmed *T. elegans* traditional use for tuberculosis by demonstrating activity of extracts against *Mycobacterium tuberculosis*, future studies should investigate their use *in vivo* (Luo et al., 2011).

###

### Other Families

*Vaccinium macrocarpon* Aiton (Ericaceae), or cranberry, is a plant native to the eastern part of North America. It is widely known for its use in the prevention of urinary tract infections (UTIs), especially among Native American people (Borchers et al., 2000). Its fruit showed MICs of 20-80 µg/mL against *Staphylococcus epidermidis*, *S. aureus* and MRSA (LaPlante et al., 2012). However, in the same study, it had no effect on the growth of *Escherichia coli* (MIC > 10 mg/mL), the main causative agent of UTIs. Indeed, *V. macrocarpon* does not act as a bactericidal or bacteriostatic agent in UTIs, but its MOA is rather the inhibition of P-fimbriated *E. coli* attachment to uroepithelial cells (Foo et al., 2000). The main compound responsible for this activity is a condensed tannin called proanthocyanidin (PAC) (Howell, 2007). Although PAC does not have growth inhibitory activity on *E. coli*, it has been reported to inhibit the growth of *S. aureus* (Hui et al., 2004). Several clinical trials have investigated the role of *V. macrocarpon* in the prevention of UTIs. One metanalysis of 13 randomized controlled trials concluded that cranberry-containing products may protect against UTIs in certain populations (i.e., women with recurrent UTIs, female populations, children) (Wang et al., 2012). Another metanalysis of 7 randomized trials conducted in healthy women indicated a beneficial effect of *V. macrocarpon* in the prevention of uncomplicated recurrent UTI (Fu et al., 2017). However, it was also noted that the lack of standardization for cranberry-containing products could lead to inefficacy and thus contribute to conflicting results in some metanalyses (Sánchez-Patán et al., 2012;Zhao et al., 2018).

**References Cited**

Akarca, G. (2019). Composition and Antibacterial Effect on Food Borne Pathogens of *Hibiscus surrattensis* L. Calyces Essential Oil. *Ind. Crop Prod.* 137**,** 285-289.

Al-Daihan, S., Al-Faham, M., Al-Shawi, N., Almayman, R., Brnawi, A., Zargar, S., and Bhat, R.S. (2013). Antibacterial Activity and Phytochemical Screening of Some Medicinal Plants Commonly used in Saudi Arabia against Selected Pathogenic Microorganisms. *J. King Saud. Univ. Sci.* 25**,** 115-120.

Alarcón-Alonso, J., Zamilpa, A., Aguilar, F.A., Herrera-Ruiz, M., Tortoriello, J., and Jimenez-Ferrer, E. (2012). Pharmacological Characterization of the Diuretic Effect of *Hibiscus sabdariffa* Linn (Malvaceae) Extract. *J. Ethnopharmacol.* 139**,** 751-756.

Ali, B.H., Blunden, G., Tanira, M.O., and Nemmar, A. (2008). Some Phytochemical, Pharmacological and Toxicological Properties of Ginger (*Zingiber officinale* Roscoe): A Review of Recent Research. *Food Chem. Toxicol.* 46**,** 409-420.

Bag, A., Bhattacharyya, S.K., and Chattopadhyay, R.R. (2013). The Development of *Terminalia chebula* Retz. (Combretaceae) in Clinical Research. *Asian Pac. J. Trop. Med.* 3**,** 244-252.

Bai, S., Bharti, P., Seasotiya, L., Malik, A., and Dalal, S. (2015). *In Vitro* Screening and Evaluation of some Indian Medicinal Plants for their Potential to Inhibit Jack Bean and Bacterial Ureases Causing Urinary Infections. *Pharm. Biol.* 53**,** 326-333.

Bonneau, N., Baloul, L., Bajin Ba Ndob, I., Sénéjoux, F., and Champy, P. (2017). The Fruit of *Annona squamosa* L. as a Source of Environmental Neurotoxins: From Quantification of Squamocin to Annotation of Annonaceous Acetogenins by LC–MS/MS analysis. *Food Chem.* 226**,** 32-40.

Borchers, A.T., Keen, C.L., Stern, J.S., and Gershwin, M.E. (2000). Inflammation and Native American Medicine: the Role of Botanicals. *Am. J. Clin. Nutr.* 72**,** 339-347.

Bouhdid, S., Abrini, J., Amensour, M., Zhiri, A., Espuny, M.J., and Manresa, A. (2010). Functional and Ultrastructural Changes in *Pseudomonas aeruginosa* and *Staphylococcus aureus* Cells Induced by *Cinnamomum verum* Essential Oil. *J. Appl. Microbiol.* 109**,** 1139-1149.

Burman, S., Bhattacharya, K., Mukherjee, D., and Chandra, G. (2018). Antibacterial Efficacy of Leaf Extracts of *Combretum album* Pers. against some Pathogenic Bacteria. *BMC Complement. Altern. Med.* 18**,** 213.

Chakotiya, A.S., Chawla, R., Thakur, P., Tanwar, A., Narula, A., Grover, S.S., Goel, R., Arora, R., and Sharma, R.K. (2016). *In Vitro* Bactericidal Activity of Promising Nutraceuticals for Targeting Multidrug Resistant *Pseudomonas aeruginosa*. *Nutrition* 32**,** 890-897.

Chakraborty, B., Nath, A., Saikia, H., and Sengupta, M. (2014). Bactericidal Activity of Selected Medicinal Plants against Multidrug Resistant Bacterial Strains from Clinical Isolates. *Asian Pac. J. Trop. Med.* 7**,** S435-S441.

Cheikhyoussef, A., Shapi, M., Matengu, K., and Mu Ashekele, H. (2011). Ethnobotanical Study of Indigenous Knowledge on Medicinal Plant use by Traditional Healers in Oshikoto Region, Namibia. *J. Ethnobiology Ethnomedicine* 7**,** 10.

Chen, I.N., Chang, C.-C., Ng, C.-C., Wang, C.-Y., Shyu, Y.-T., and Chang, T.-L. (2008). Antioxidant and Antimicrobial Activity of Zingiberaceae Plants in Taiwan. *Plant Foods Hum. Nutr.* 63**,** 15-20.

Da-Costa-Rocha, I., Bonnlaender, B., Sievers, H., Pischel, I., and Heinrich, M. (2014). *Hibiscus sabdariffa* L. – A Phytochemical and Pharmacological Review. *Food Chem.* 165**,** 424-443.

Daimei, P., and Kumar, Y. (2014). Ethnobotanical Uses of Gingers in Tamenglong District, Manipur, Northeast India. *Genet. Resour. Crop Evol.* 61**,** 273-285.

Dholvitayakhun, A., Cushnie, T.P.T., and Trachoo, N. (2012). Antibacterial Activity of Three Medicinal Thai Plants against *Campylobacter jejuni* and other Foodborne Pathogens. *Nat. Prod. Res.* 26**,** 356-363.

Djeussi, D.E., Noumedem, J.a.K., Seukep, J.A., Fankam, A.G., Voukeng, I.K., Tankeo, S.B., Nkuete, A.H.L., and Kuete, V. (2013). Antibacterial Activities of Selected Edible Plants Extracts against Multidrug-Resistant Gram-negative Bacteria. *BMC Complement. Altern. Med.* 13**,** 164.

El Babili, F., Roques, C., Haddioui, L., Bellvert, F., Bertrand, C., and Chatelain, C. (2012). Velamo do Campo: Its Volatile Constituents, Secretory Elements, and Biological Activity. *J. Med. Food* 15**,** 671-676.

Eng-Chong, T., Yean-Kee, L., Chin-Fei, C., Choon-Han, H., Sher-Ming, W., Li-Ping, C.T., Gen-Teck, F., Khalid, N., Abd Rahman, N., Karsani, S.A., Othman, S., Othman, R., and Yusof, R. (2012). *Boesenbergia rotunda*: From Ethnomedicine to Drug Discovery. *Evid. Based Complement. Alternat. Med.* **,** ID473637.

Faboro, E.O., Wichitnithad, W., Fadare, O.A., Akinpelu, D.A., and Obafemi, C.A. (2016). Antibacterial and Antioxidant Activities and Phytochemical Screening of Aqueous Methanol Extracts of Eight Nigerian Medicinal and Aromatic Plants. *J. Pharm. Res.* 10**,** 523-532.

Foo, L.Y., Lu, Y., Howell, A.B., and Vorsa, N. (2000). A-Type Proanthocyanidin Trimers from Cranberry that Inhibit Adherence of Uropathogenic P-Fimbriated *Escherichia coli*. *J. Nat. Prod.* 63**,** 1225-1228.

Fu, Z., Liska, D., Talan, D., and Chung, M. (2017). Cranberry Reduces the Risk of Urinary Tract Infection Recurrence in Otherwise Healthy Women: A Systematic Review and Meta-Analysis. *J. Nutr.* 147**,** 2282-2288.

Gaus, K., Huang, Y., Israel, D.A., Pendland, S.L., Adeniyi, B.A., and Mahady, G.B. (2009). Standardized Ginger (*Zingiber officinale*) Extract Reduces Bacterial Load and Suppresses Acute and Chronic Inflammation in Mongolian Gerbils Infected with cagA+ *Helicobacter pylori*. *Pharm. Biol.* 47**,** 92-98.

Gaya, C.H., Kawaka, J.F., Muchugi, A., and Ngeranwa, J.J. (2013). Variation of Alkaloids in the Kenyan *Zanthoxylum gilletii* (De Wild Waterman). *Afr. J. Plant Sci.* 7**,** 438-444.

Gbolade, A. (2012). Ethnobotanical Study of Plants used in Treating Hypertension in Edo State of Nigeria. *J. Ethnopharmacol.* 144**,** 1-10.

Ghasemzadeh, A., Jaafar, H.Z.E., Karimi, E., and Rahmat, A. (2014). Optimization of Ultrasound-Assisted Extraction of Flavonoid Compounds and their Pharmaceutical Activity from Curry Leaf (*Murraya koenigii* L.) using Response Surface Methodology. *BMC Complement. Altern. Med.* 14**,** 318.

Ghosh, T., Mitra, P., and Mitra, P.K. (2018). Isolation of a Compound from *Murrya koenigii* (Linn.) Spreng Wettst Leaves and Studies on its Antibacterial Activity. *Eur. J. Biomed. Pharm. Sci.* 5**,** 1-6.

Gilani, A.H., Shah, A.J., Ghayur, M.N., and Majeed, K. (2005). Pharmacological Basis for the use of Turmeric in Gastrointestinal and Respiratory Disorders. *Life Sci.* 76**,** 3089-3105.

Gupta, D., and Jain, A. (2015). Effect of Cinnamon Extract and Chlorhexidine Gluconate (0.2%) on the Clinical Level of Dental Plaque and Gingival Health: A 4-Week, Triple-Blind Randomized Controlled Trial. *J. Int. Acad. Periodontol.* 17**,** 91-98.

Haji Faraji, M., and Haji Tarkhani, A.H. (1999). The Effect of Sour Tea (*Hibiscus sabdariffa*) on Essential Hypertension. *J. Ethnopharmacol.* 65**,** 231-236.

Hasan, S., Danishuddin, M., and Khan, A.U. (2015). Inhibitory Effect of *Zingiber officinale* Towards *Streptococcus mutans* Virulence and Caries Development: *In Vitro* and *In Vivo* studies. *BMC Microbiol.* 15**,** 1.

Hassan, S.T.S., Berchová, K., Majerová, M., Pokorná, M., and Švajdlenka, E. (2016). *In Vitro* Synergistic Effect of *Hibiscus sabdariffa* Aqueous Extract in Combination with Standard Antibiotics against *Helicobacter pylori* Clinical Isolates. *Pharm. Biol.* 54**,** 1736-1740.

Heneidak, S., Grayer, R.J., Kite, G.C., and Simmonds, M.S.J. (2006). Flavonoid Glycosides from Egyptian Species of the Tribe Asclepiadeae (Apocynaceae, subfamily Asclepiadoideae). *Biochem. Syst. Ecol.* 34**,** 575-584.

Horn, J.W., Van Ee, B.W., Morawetz, J.J., Riina, R., Steinmann, V.W., Berry, P.E., and Wurdack, K.J. (2012). Phylogenetics and the Evolution of Major Structural Characters in the Giant Genus Euphorbia L. (Euphorbiaceae). *Mol. Phylogenet. Evol.* 63**,** 305-326.

Howell, A.B. (2007). Bioactive Compounds in Cranberries and their Role in Prevention of Urinary Tract Infections. *Mol. Nutr. Food Res.* 51**,** 732-737.

Hui, J., Choy, J., Suwandaratne, S.P., Shervill, J., Gan, B.S., Howard, J.C., and Reid, G. (2004). Antimicrobial Activity of *Vaccinium macrocarpon* (Cranberry) Produced Proanthocyanidin (PAC) on the Growth and Adhesion Properties of *Staphylococcus aureus*. *J. Food Sci. Nutr.* 9**,** 29-33.

Jamkhande, P.G., and Wattamwar, A.S. (2015). *Annona reticulata* Linn. (Bullock's heart): Plant Profile, Phytochemistry and Pharmacological Properties. *J. Tradit. Complement. Med.* 5**,** 144-152.

Kim, H.G., Cho, J.H., Jeong, E.Y., Lim, J.H., Lee, S.H., and Lee, H.S. (2006). Growth-Inhibiting Activity of Active Component Isolated from *Terminalia chebula* Fruits against Intestinal Bacteria. *J. Food Prot.* 69**,** 2205-2209.

Koosirirat, C., Linpisarn, S., Changsom, D., Chawansuntati, K., and Wipasa, J. (2010). Investigation of the anti-inflammatory effect of Curcuma longa in Helicobacter pylori-infected patients. *International Immunopharmacology* 10**,** 815-818.

Koudouvo, K., Karou, D.S., Kokou, K., Essien, K., Aklikokou, K., Glitho, I.A., Simpore, J., Sanogo, R., De Souza, C., and Gbeassor, M. (2011). An Ethnobotanical Study of Antimalarial Plants in Togo Maritime Region. *J. Ethnopharmacol.* 134**,** 183-190.

Laplante, K.L., Sarkisian, S.A., Woodmansee, S., Rowley, D.C., and Seeram, N.P. (2012). Effects of Cranberry Extracts on Growth and Biofilm Production of *Escherichia coli* and *Staphylococcus* species. *Phytother. Res.* 26**,** 1371-1374.

Lemes, R.S., Alves, C.C.F., Estevam, E.B.B., Santiago, M.B., Martins, C.H.G., Santos, T.C.L.D.O.S., Crotti, A.E.M., and Miranda, M.L.D. (2018). Chemical Composition and Antibacterial Activity of Essential Oils from *Citrus aurantifolia* Leaves and Fruit Peel against Oral Pathogenic Bacteria. *An. Acad. Bras. Cienc.* 90**,** 1285-1292.

Li, L., Li, Z.-W., Yin, Z.-Q., Wei, Q., Jia, R.-Y., Zhou, L.-J., Xu, J., Song, X., Zhou, Y., Du, Y.-H., Peng, L.-C., Kang, S., and Yu, W. (2014). Antibacterial Activity of Leaf Essential Oil and its Constituents from *Cinnamomum longepaniculatum*. *Int. J. Clin. Exp. Med.* 7**,** 1721-1727.

Liaw, C.-C., Liou, J.-R., Wu, T.-Y., Chang, F.-R., and Wu, Y.-C. (2016). Acetogenins from Annonaceae. *Progress in the Chemistry of Organic Natural Products 101***,** 113-230.

Lim, T.K. (2016). Curcuma longa. *Edible Medicinal and Non-Medicinal Plants: Volume 12 Modified Stems, Roots, Bulbs***,** 241-362.

Limsuwan, S., and Voravuthikunchai, S.P. (2013). Bactericidal, Bacteriolytic, and Antibacterial Virulence Activities of *Boesenbergia pandurata* (Roxb) Schltr Extract against *Streptococcus pyogenes*. *Trop. J. Pharm. Res.* 12**,** 1023-1028.

Lin, J., Dou, J., Xu, J., and Aisa, H.A. (2012). Chemical Composition, Antimicrobial and Antitumor Activities of the Essential Oils and Crude Extracts of *Euphorbia macrorrhiza*. *Molecules* 17**,** 5030-5039.

Lindeman, Z., Waggoner, M., Batdorff, A., and Humphreys, T.L. (2014). Assessing the Antibiotic Potential of Essential Oils against *Haemophilus ducreyi*. *BMC Complement. Altern. Med.* 14**,** 172.

Luo, X., Pires, D., Aínsa, J.A., Gracia, B., Mulhovo, S., Duarte, A., Anes, E., and Ferreira, M.-J.U. (2011). Antimycobacterial Evaluation and Preliminary Phytochemical Investigation of Selected Medicinal Plants Traditionally used in Mozambique. *J. Ethnopharmacol.* 137**,** 114-120.

Madureira, A.M., Ramalhete, C., Mulhovo, S., Duarte, A., and Ferreira, M.-J.U. (2012). Antibacterial Activity of some African Medicinal Plants used Traditionally Against Infectious Diseases. *Pharm. Biol.* 50**,** 481-489.

Mahady, G.B., Bhamarapravati, S., Adeniyi, B.A., Doyle, B., Locklear, T., Slover, C., and Pendland, S.L. (2006). Traditional Thai Medicines Inhibit *Helicobacter pylori* *In-Vitro* and *In-Vivo*: Support for Ethnomedical use. *Ethnobotany Research and Applications* 4**,** 8.

Moteetee, A., and Van Wyk, B.E. (2011). The Medical Ethnobotany of Lesotho: A Review. *Afr. Biodivers. Conserv.* 41**,** 20.

Moudi, M., Go, R., Yien, C.Y.S., and Nazre, M. (2013). Vinca Alkaloids. *Int. J. Prev. Med.* 4**,** 1231-1235.

Muthu, C., Ayyanar, M., Raja, N., and Ignacimuthu, S. (2006). Medicinal Plants used by Traditional Healers in Kancheepuram District of Tamil Nadu, India. *J. Ethnobiology Ethnomedicine* 2**,** 43.

Nabati, F., Mojab, F., Habibi-Rezaei, M., Bagherzadeh, K., Amanlou, M., and Yousefi, B. (2012). Large Scale Screening of Commonly used Iranian Traditional Medicinal Plants against Urease Activity. *Daru* 20**,** 72.

Nagappan, T., Segaran, T.C., Wahid, M.E.A., Ramasamy, P., and Vairappan, C.S. (2012). Efficacy of Carbazole Alkaloids, Essential Oil and Extract of *Murraya koenigii* in Enhancing Subcutaneous Wound Healing in Rats. *Molecules* 17**,** 14449-14463.

Narang, N., and Jiraungkoorskul, W. (2016). Anticancer Activity of Key Lime, *Citrus aurantifolia*. *Pharmacogn. Rev.* 10**,** 118-122.

Nasir, M., Tafess, K., and Abate, D. (2015). Antimicrobial Potential of the Ethiopian *Thymus schimperi* Essential Oil in Comparison with others against certain Fungal and Bacterial Species. *BMC Complement. Altern. Med.* 15**,** 260.

Nayak, B.S., and Pinto Pereira, L.M. (2006). *Catharanthus roseus* Flower Extract has Wound-Healing Activity in Sprague Dawley Rats. *BMC Complement. Altern. Med.* 6**,** 41.

Nciki, S., Vuuren, S., Van Eyk, A., and De Wet, H. (2016). Plants Used to Treat Skin Diseases in Northern Maputaland, South Africa: Antimicrobial Activity and *In Vitro* Permeability Studies. *Pharm. Biol.* 54**,** 2420-2436.

Nejat, N., Valdiani, A., Cahill, D., Tan, Y.-H., Maziah, M., and Abiri, R. (2015). Ornamental Exterior versus Therapeutic Interior of Madagascar Periwinkle (*Catharanthus roseus*): The Two Faces of a Versatile Herb. *Evid. Based Complement. Alternat. Med.* **,** ID982412.

Nlm (2020). National Library of Medicine (US). [cited 2020 Mar 31]. Available from: <http://clinicaltrials.gov>.

Nyila, M.A., Leonard, C.M., Hussein, A.A., and Lall, N. (2012). Activity of South African Medicinal Plants against *Listeria monocytogenes* Biofilms, and Isolation of Active Compounds from *Acacia karroo*. *S. Afr. J. Bot.* 78**,** 220-227.

Pallant, C.A., Cromarty, A.D., and Steenkamp, V. (2012). Effect of an Alkaloidal Fraction of *Tabernaemontana elegans* (Stapf.) on Selected Micro-organisms. *J. Ethnopharmacol.* 140**,** 398-404.

Pant, N., Misra, H., and Jain, D.C. (2013). Phytochemical Investigation of Ethyl Acetate Extract from *Curcuma aromatica* Salisb. Rhizomes. *Arab. J. Chem.* 6**,** 279-283.

Park, M., Bae, J., and Lee, D.-S. (2008). Antibacterial Activity of [10]-gingerol and [12]-gingerol Isolated from Ginger Rhizome against Periodontal Bacteria. *Phytother. Res.* 22**,** 1446-1449.

Pereira, F., Madureira, A.M., Sancha, S., Mulhovo, S., Luo, X., Duarte, A., and Ferreira, M.-J.U. (2016). *Cleistochlamys kirkii* Chemical Constituents: Antibacterial Activity and Synergistic Effects against Resistant *Staphylococcus aureus* Strains. *J. Ethnopharmacol.* 178**,** 180-187.

Pham, H.N.T., Sakoff, J.A., Vuong, Q.V., Bowyer, M.C., and Scarlett, C.J. (2018). Screening Phytochemical Content, Antioxidant, Antimicrobial and Cytotoxic Activities of *Catharanthus roseus* (L.) G. Don Stem Extract and its Fractions. *Biocatal. Agric. Biotechnol.* 16**,** 405-411.

Pieters, L., De Bruyne, T., Van Poel, B., Vingerhoets, R., Totté, J., Vanden Berghe, D., and Vlietinck, A. (1995). *In Vivo* Wound Healing Activity of Dragon's Blood (*Croton* spp.), a Traditional South American Drug, and its Constituents. *Phytomedicine* 2**,** 17-22.

Prucksunand, C., Indrasukhsri, B., Leethochawalit, M., and Hungspreugs, K. (2001). Phase II Clinical Trial on Effect of the Long Turmeric (*Curcuma longa* Linn) on Healing of Peptic Ulcer. *Southeast Asian J. Trop. Med. Public Health* 32**,** 208-215.

Quílez, A.M., Fernández-Arche, M.A., García-Giménez, M.D., and De La Puerta, R. (2018). Potential Therapeutic Applications of the Genus *Annona*: Local and Traditional Uses and Pharmacology. *J. Ethnopharmacol.* 225**,** 244-270.

Rahman, M.M., and Gray, A.I. (2005). A Benzoisofuranone Derivative and Carbazole Alkaloids from *Murraya koenigii* and their Antimicrobial Activity. *Phytochemistry* 66**,** 1601-1606.

Ranasinghe, P., Pigera, S., Premakumara, G.a.S., Galappaththy, P., Constantine, G.R., and Katulanda, P. (2013). Medicinal Properties of ‘True’ Cinnamon (*Cinnamomum zeylanicum*): A Systematic Review. *BMC Complement. Altern. Med.* 13**,** 275.

Rao, R.R. (1981). Ethnobotany of Meghalaya: Medicinal plants used by Khasi and Garo tribes. *Econ. Bot.* 35**,** 4-9.

Revathi, S., and Malathy, N.S. (2013). Antibacterial Activity of Rhizome of *Curcuma aromatica* and Partial Purification of Active Compounds. *Indian J. Pharm. Sci.* 75**,** 732-735.

Rosti, L., and Gastaldi, G. (2005). Chronic Salmonellosis and Cinnamon. *Pediatrics* 116**,** 1057.

Salatino, A., Salatino, M.L.F., and Negri, G. (2007). Traditional Uses, Chemistry and Pharmacology of *Croton* species (Euphorbiaceae). *J. Brazil Chem. soc.* 18**,** 11-33.

Salib, J.Y. (2014). "Chapter 12 - Polyphenolic Compounds from Flowers of Hibiscus: Characterization and Bioactivity," in *Polyphenols in Plants,* ed. R.R. Watson. (San Diego: Academic Press), 231-239.

Sánchez-Patán, F., Bartolomé, B., Martín-Alvarez, P.J., Anderson, M., Howell, A., and Monagas, M. (2012). Comprehensive Assessment of the Quality of Commercial Cranberry Products. Phenolic Characterization and *In Vitro* Bioactivity. *J. Agric. Food Chem.* 60**,** 3396-3408.

Sasisekharan, R., Lee, S.L., Rosenberg, A., and Walker, L.A. (2019). *The Science and Regulations of Naturally Derived Complex Drugs.* Cham, Switzerland: Springer.

Seukep, J.A., Ngadjui, B., and Kuete, V. (2015). Antibacterial activities of *Fagara macrophylla*, *Canarium schweinfurthii, Myrianthus arboreus, Dischistocalyx grandifolius* and *Tragia benthamii* against Multi-drug Resistant Gram-negative Bacteria. *SpringerPlus* 4**,** 567.

Shekar, B.R.C., Nagarajappa, R., Jain, R., Suma, S., Mruthunjaya, K., and Thakur, R. (2019). Evaluating the Antimicrobial Efficacy of an Innovative, Novel Herbal Formulation on Dental Caries and Plaque Microorganisms - A Clinical Research. *Biomed. Pharm. J.* 12**,** 1633-1645.

Sinan, K.I., Zengin, G., Bene, K., and Mahomoodally, M.F. (2019). Chemistry and Pharmacology of Three Antiplasmodial Traditional Medicinal Plants from Tropical Africa – A Review. *S. Afr. J. Bot.* 126**,** 265-276.

Stevens, P.F. (2001 Onwards). Angiosperm Phylogeny Website. Version 14, July 2017.

Suguna, L., Singh, S., Sivakumar, P., Sampath, P., and Chandrakasan, G. (2002). Influence of *Terminalia chebula* on Dermal Wound Healing in Rats. *Phytother. Res.* 16**,** 227-231.

Sukandar, E.Y., Kurniati, N.F., Wikaningtyas, P., and Agprikani, D. (2016). Antibacterial Interaction of Combination of Ethanolic Extract of *Zingiber officinale* var. *rubrum* rhizome, *Boesenbergia pandurata* Rhizome, and *Stevia rebaudiana* Leaves with Certain Antibiotics against Infectious Mouth Microbial. *Asian J. Pharm. Clin. Res.* 9**,** 332-335.

Tala, D.S., Gatsing, D., Fodouop, S.P.C., Fokunang, C., Kengni, F., and Djimeli, M.N. (2015). *In Vivo* Anti-*Salmonella* Activity of Aqueous Extract of *Euphorbia prostrata* Aiton (Euphorbiaceae) and its Toxicological Evaluation. *Asian Pac. J. Trop. Med.* 5**,** 310-318.

Tankeo, S.B., Damen, F., Awouafack, M.D., Mpetga, J., Tane, P., Eloff, J.N., and Kuete, V. (2015). Antibacterial activities of the methanol extracts, fractions and compounds from Fagara tessmannii. *Journal of Ethnopharmacology* 169**,** 275-279.

Teethaisong, Y., Pimchan, T., Srisawat, R., Hobbs, G., and Eumkeb, G. (2018). *Boesenbergia rotunda* (L.) Mansf. Extract Potentiates the Antibacterial Activity of some β-lactams against β-lactam-resistant Staphylococci. *J. Glob. Antimicrob. Re.* 12**,** 207-213.

Ticktin, T., and Dalle, S.P. (2005). Medicinal Plant Use in the Practice of Midwifery in Rural Honduras. *J. Ethnopharmacol.* 96**,** 233-248.

Tradtrantip, L., Namkung, W., and Verkman, A.S. (2010). Crofelemer, an Antisecretory Antidiarrheal Proanthocyanidin Oligomer Extracted from *Croton lechleri* Targets Two Distinct Intestinal Chloride Channels. *Mol. Pharmacol.* 77**,** 69.

Tsouh Fokou, P.V., Kissi-Twum, A.A., Yeboah-Manu, D., Appiah-Opong, R., Addo, P., Tchokouaha Yamthe, L.R., Ngoutane Mfopa, A., Fekam Boyom, F., and Nyarko, A.K. (2016). *In Vitro* Activity of Selected West African Medicinal Plants against *Mycobacterium ulcerans* Disease. *Molecules* 21**,** 445.

Vaillancourt, K., Lebel, G., Yi, L., and Grenier, D. (2018). *In Vitro* Antibacterial Activity of Plant Essential Oils against *Staphylococcus hyicus* and *Staphylococcus aureus*, the Causative Agents of Exudative Epidermitis in Pigs. *Arch. Microbiol.* 200**,** 1001-1007.

Van Der Heijden, R., Brouwer, R.L., Verpoorte, R., Van Beek, T.A., Harkes, P.a.A., and Svendsen, A.B. (1986). Indole Alkaloids from *Tabernaemontana elegans*. *Planta Med.* 52**,** 144-147.

Van Vuuren, S.F., and Naidoo, D. (2010). An Antimicrobial Investigation of Plants used Traditionally in Southern Africa to Treat Sexually Transmitted Infections. *J. Ethnopharmacol.* 130**,** 552-558.

Van Vuuren, S.F., and Viljoen, A.M. (2008). *In Vitro* Evidence of Phyto-synergy for Plant Part Combinations of *Croton gratissimus* (Euphorbiaceae) used in African Traditional Healing. *J. Ethnopharmacol.* 119**,** 700-704.

Voukeng, I.K., Beng, V.P., and Kuete, V. (2016). Antibacterial Activity of Six Medicinal Cameroonian Plants against Gram-positive and Gram-negative Multidrug Resistant Phenotypes. *BMC Complement. Altern. Med.* 16**,** 388.

Voukeng, I.K., Beng, V.P., and Kuete, V. (2017a). Multidrug Resistant Bacteria are Sensitive to *Euphorbia prostrata* and Six Others Cameroonian Medicinal Plants Extracts. *BMC Res. Notes* 10**,** 321.

Voukeng, I.K., Nganou, B.K., Sandjo, L.P., Celik, I., Beng, V.P., Tane, P., and Kuete, V. (2017b). Antibacterial Activities of the Methanol Extract, Fractions and Compounds from *Elaeophorbia drupifera* (Thonn.) Stapf. (Euphorbiaceae). *BMC Complement. Altern. Med.* 17**,** 28.

Wang, C.-H., Fang, C.-C., Chen, N.-C., Liu, S.S.-H., Yu, P.-H., Wu, T.-Y., Chen, W.-T., Lee, C.-C., and Chen, S.-C. (2012). Cranberry-Containing Products for Prevention of Urinary Tract Infections in Susceptible Populations: A Systematic Review and Meta-analysis of Randomized Controlled Trials. *Arch. Intern. Med.* 172**,** 988-996.

Wu, M.L., Deng, J.F., Wu, J.C., Fan, F.S., and Yang, C.F. (2004). Severe Bone Marrow Depression Induced by an Anticancer Herb *Cantharanthus roseus*. *J. Toxicol. Clin. Toxicol.* 42**,** 667-671.

Yagi, S., Babiker, R., Tzanova, T., and Schohn, H. (2016). Chemical Composition, Antiproliferative, Antioxidant and Antibacterial Activities of Essential Oils from Aromatic Plants Growing in Sudan. *Asian Pac. J. Trop. Med.* 9**,** 763-770.

Ye, H., Shen, S., Xu, J., Lin, S., Yuan, Y., and Jones, G.S. (2013). Synergistic Interactions of Cinnamaldehyde in Combination with Carvacrol against Food-Borne Bacteria. *Food Control* 34**,** 619-623.

Yu, L., Ren, J.X., Nan, H.M., and Liu, B.F. (2015). Identification of Antibacterial and Antioxidant Constituents of the Essential Oils of *Cynanchum chinense* and *Ligustrum compactum*. *Nat. Prod. Res.* 29**,** 1779-1782.

Zhao, S., Liu, H., and Gu, L. (2018). American Cranberries and Health Benefits – An Evolving Story of 25 Years. *J. Sci. Food Agric.* n/a.

Zheng, X.-L., and Xing, F.-W. (2009). Ethnobotanical Study on Medicinal Plants around Mt.Yinggeling, Hainan Island, China. *J. Ethnopharmacol.* 124**,** 197-210.
