# Supplementary figures and images for: A Systematic Review of Plants With Antibacterial Activities: A Taxonomic and Phylogenetic Perspective
Source: Front Pharmacol. 2021 Jan 8;11:586548. doi: 10.3389/fphar.2020.586548 (PMC7821031; doi:10.3389/fphar.2020.586548)

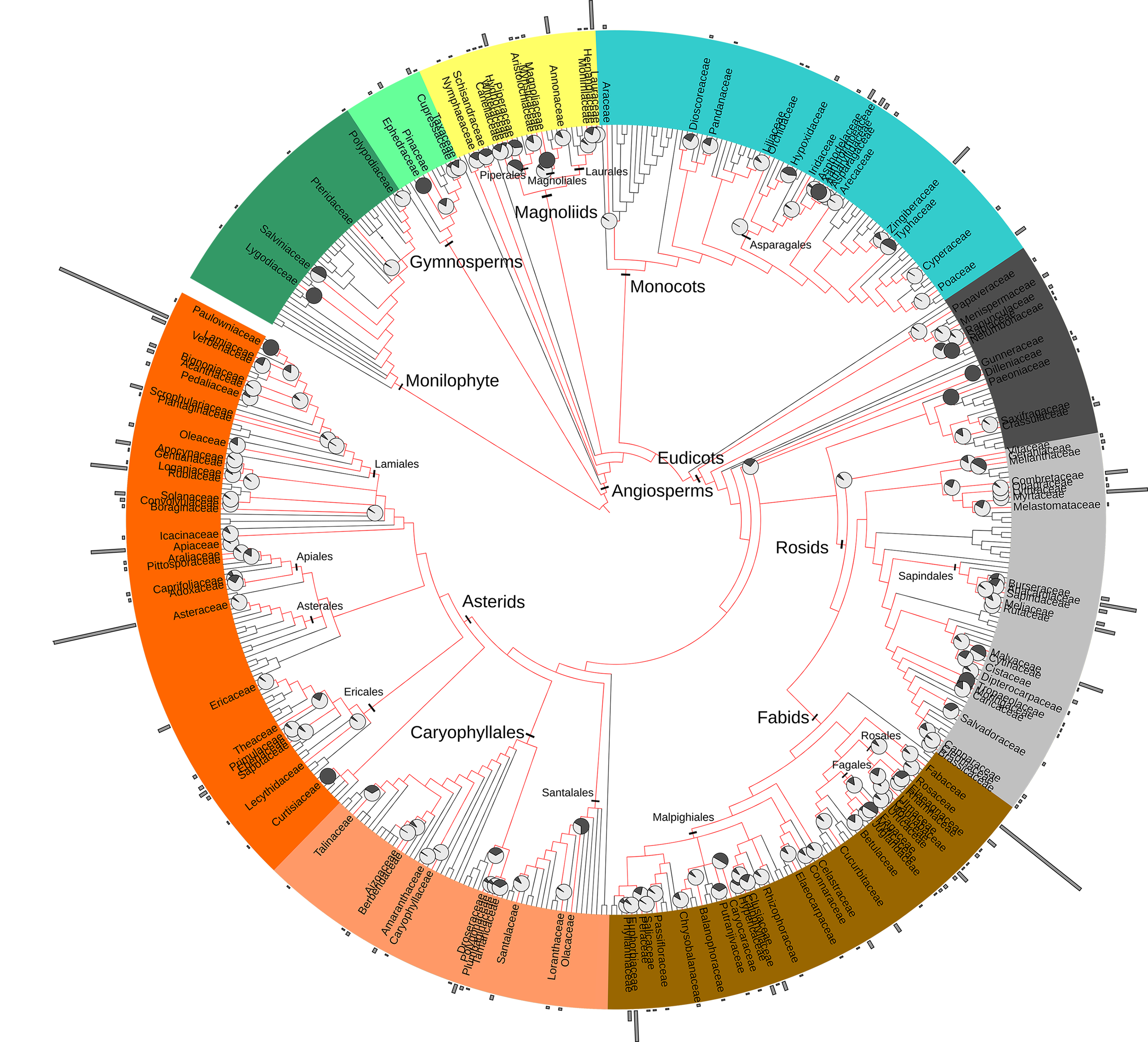

Supplement: Supplementary file 3 [file image1.tif]
